# Understanding the bidirectional relationship between chronic respiratory disease and cardiovascular disease using genetic evidence

**DOI:** 10.1136/thorax-2024-222908

**Published:** 2025-11-15

**Authors:** Naesilla Naesilla, Jennifer K Quint, Verena Zuber

**Affiliations:** 1 School of Public Health, Imperial College London, London, UK; 2 MRC Centre for Environment and Health, Imperial College London, London, UK; 3 UK Dementia Research Institute, Imperial College London, London, UK

**Keywords:** Clinical Epidemiology, Asthma, Pulmonary Disease, Chronic Obstructive, Smoking

## Abstract

**Background:**

Chronic respiratory diseases (CRDs) and cardiovascular diseases (CVDs) are leading global health burdens. Despite being common, CRD and CVD comorbidity is often underestimated due to overlapping symptoms and risk factors. Consequently, their relationship remains unclear.

**Aims and objectives:**

To determine the bidirectional genetic relationship between CRD and CVD and explore smoking and inflammation as potentially shared joint risk factors.

**Methods:**

We conducted bidirectional Mendelian randomisation (MR) to explore CRD–CVD relationships. Summary statistics from genome-wide association studies were retrieved for chronic obstructive pulmonary disease (COPD), asthma, coronary artery disease (CAD), myocardial infarction (MI), heart failure, atrial fibrillation (AF) and ischaemic stroke (IS). We performed additional analysis including univariable MR for smoking, multivariable MR adjusting for smoking and cis-MR to investigate the role of inflammatory markers.

**Results:**

Our MR analysis found limited genetic evidence of relationships between CRD and CVD, and vice versa. However, a nominally significant genetic association was observed between asthma and an increased risk of AF (OR inverse-variance weighted (OR_IVW_) 1.036, 95% CI 1.003 to 1.070), remaining weakly significant after adjusting for smoking (OR_IVW_ 1.040, 95% CI 1.008 to 1.074). Genetically predicted lifetime smoking strongly increased all CRD and CVD risk. Additionally, genetically proxied IL6R concentration associated with increased asthma risk and decreased CAD, MI, AF and IS risk, while IL1RN decreased COPD risk but increased CAD and MI risk.

**Conclusions:**

While we found limited genetic evidence linking CRD and CVD, smoking and inflammatory markers commonly affect both. These findings highlight the complexity of CRD–CVD comorbidities, whose pathophysiology likely does not involve direct causation of each other.

**WHAT IS ALREADY KNOWN ON THIS TOPIC**

- Chronic respiratory diseases (CRDs), such as chronic obstructive pulmonary disease (COPD) and asthma, often coexist with cardiovascular disease (CVD), increasing the clinical burden. Previous studies have suggested that the comorbidities share overlapping risk factors and symptoms, contributing to underdiagnosis. Both asthma and COPD have been studied in relation to CVD comorbidities using Mendelian randomisation (MR) studies, in the hope of understanding their relationships and overcoming observational and clinical trial limitations. However, studies vary in results, the population used and the traits of CVD studied.

**WHAT THIS STUDY ADDS**

- We conducted bidirectional MR using updated genome-wide association studies and uniform analytical pipeline. Our study found no clear genetic evidence of a bidirectional association between CRD and CVD. These findings suggest that the observed associations in clinical studies may not indicate direct causal effects between the diseases.

**HOW THIS STUDY MIGHT AFFECT RESEARCH, PRACTICE OR POLICY**

- As this study highlights the absence of a direct causal relationship between CRD and CVD, we still need to fully understand why comorbidities and clinical associations of these diseases are frequently observed in clinical settings. Therefore, we require further studies to uncover the underlying mechanisms essential for improving prevention and treatment strategies.

## Introduction

 Chronic respiratory diseases (CRDs) are defined as the pathology of the airway and other structures of the lungs. In 2019, CRDs approximately affected 454.6 million people, leading to about 4 million deaths, making them the third leading cause of global mortality.^1^ Among all CRDs, chronic obstructive pulmonary disease (COPD) and asthma are the most prevalent and clinically significant, accounting for the vast majority of CRD cases globally.^2^ Separately, cardiovascular diseases (CVDs) have remained the leading global health burden for nearly three decades, with total prevalent cases doubling from 271 million in 1990 to 523 million in 2019, and mortality increasing by 1.5 times to 18.6 million deaths.^3^

Beyond their individual burden, increasing evidence suggests that CRD and CVD frequently coexist. Observational studies report that the prevalence of CVD in CRD patients ranges from 20% to 70%.^4 5^ Conversely, some studies report the prevalence of CRD in CVD patients ranges from 10% to 40% depending on the underlying CVD.^6 7^ This overlap is responsible for even higher rehospitalisations, emergency room visits, mortality and healthcare costs.^8 9^ Therefore, given the significant public health burden, it is crucial to understand the nature of the relationship between CRD and CVD.

Two key shared risk factors have been consistently implicated in both CRD and CVD: smoking and inflammation. Smoking is a well-established risk factor for both CRD and CVD, making smoking cessation essential in preventive guidelines for both CRD and CVD. Moreover, smoking is known to be a potent inducer of inflammation, contributing to both local airway damage and systemic response. Nevertheless, inflammation itself, whether related to smoking or not, has been known to play a central role in the development and progression of both respiratory and cardiovascular conditions, including asthma, COPD, coronary artery disease (CAD) and stroke. These reports eventually support the ‘spill-over hypothesis’, which proposes that local inflammation in the lungs may spill into the systemic circulation, contributing to broader vascular and cardiac effects.^10 11^ Therefore, it is not surprising that inflammation has become a focus for treatment strategies. Inhaled corticosteroids has become a cornerstone of treatment for CRDs, especially asthma and COPD. However, evidence on their cardiovascular benefit remains mixed, with studies reporting either reduced risk or no effect.^12 13^ These highlight the importance of understanding the shared risk factors not only as causes of the comorbidity, but also as potential therapeutic targets.

Observational studies have struggled to answer the question and demonstrate causality partly because of intrinsic biases and variability in reporting CRD cases. They often require long follow-up to detect chronic diseases and may reveal correlations only, not true causal relationships. Interpreting associations as causal in this context is risky given the potential for unmeasured confounding and selection bias. Here, Mendelian randomisation (MR) offers a promising approach to triangulate evidence.^14^ MR can provide stronger evidence for the potentially causal relationship between CRD and CVD by evaluating genetic liability data. Moreover, MR can address some limitations of observational studies while avoiding the ethical and practical constraints of clinical trials.

In this study, we aimed to address some gaps left by previous MR studies. We used two-sample summary-level MR to assess the bidirectional relationships between major CRDs (COPD and asthma) and common, high-burden CVDs, including CAD, myocardial infarction (MI), ischaemic stroke (IS), heart failure (HF) and atrial fibrillation (AF). We selected these phenotypes not only because of their clinical relevance, but also because they are among the best-powered traits in currently available genome-wide association study (GWAS) datasets. While these diseases are also reported to be influenced by genetic predispositions and environmental exposures, we specifically focus on clarifying the role of smoking and systemic inflammation, given their well-established associations with these diseases and the availability of robust genetic instruments. Our use of updated GWAS sources and a uniform analytical pipeline minimised researchers’ degree of freedom in individual studies, enabling us to provide a comprehensive analysis that agnostically explores the bidirectional relationship between CRD and CVD.

## Methods

MR studies rely on three key assumptions for validity^14^: (1) Relevance: the variant is associated with the exposure/risk factor of interest. (2) Independence assumption: there are no confounders of the association between the instrumental variables (IVs) and the outcome. (3) Exclusion restriction: the variant does not affect the outcome directly, only possibly indirectly via the exposure. Our methodological framework was designed to satisfy these assumptions where possible and minimise potential violations.

### Data source

In brief, we retrieved publicly available summary statistics from larger, more recent and reliable GWAS, if available, from GWAS Catalog (https://www.ebi.ac.uk/gwas), except where stated otherwise. There was no restriction on the ancestry in the GWAS to be included, but in the majority, we included European population, as it represents quite a large proportion in different available biobanks. All GWAS included are listed in table 1.

**Table 1:** Data sources for current two-sample Mendelian randomisation analyses

Trait	Authors	Year	Number of participants	Ancestry	PMID
COPD	Cosentino *et al*^42^	2023	325 027 participants with valid spirometry	European	37069358
Asthma	Han *et al*^43^	2020	64 538 cases and 329 321 controls	Mixed ancestry (mostly European)	32296059
CAD	Aragam *et al*^44^	2022	181 522 cases and 984 168 controls	Mixed ancestry (mostly European)	36474045
MI	Hartiala *et al*^45^	2021	61 505 MI cases and 577 716 controls	Mixed Ancestry (mostly European)	33532862
HF	Shah *et al*^46^	2020	47 309 cases and 930 014 controls	European	31919418
AF	Nielsen *et al*^47^	2018	60 620 cases and 970 216 controls	European	30061737
IS	Mishra *et al*^48^	2022	110 182 cases (86 668 any ischaemia stroke) 1 503 898 controls	Mixed ancestry (mostly European)	36180795
Lifetime smoking	Wootton *et al*^32^	2020	462 690 current, former and never smokers	European	31689377*
Inflammatory markers	Yarmolinsky *et al*^20^	2024	59 969 participants across included studies	European	38301482†

Sex and average age were not consistently reported across all GWAS sources. For CAD (Aragam *et al*) and lifetime smoking (Wootton *et al*^32^), 46% and 54% participants were female, respectively. The average age for the latter was 56.7 years.

All summary statistics are available publicly in https://www.ebi.ac.uk/gwas/ except for lifetime smoking.

* Available in https://doi.org/10.5523/bris.10i96zb8gm0j81yz0q6ztei23d.

† Personal communication.

AF, atrial fibrillation; CAD, coronary artery disease; COPD, chronic obstructive pulmonary disease; GWAS, genome-wide association studies; HF, heart failure; IS, ischaemic stroke; MI, myocardial infarction; PMID, PubMed identifier.

### Univariable MR

#### Instrument selection

To fulfil the first assumption, we selected variants or single nucleotide polymorphisms (SNPs) with genome-wide significance threshold levels of p value <5×10^−8^. To address the probability of non-Mendelian inheritance, we pruned for linkage disequilibrium (LD) between SNPs, with correlation coefficient threshold of r^2^<0.001 and a clumping window width of 10 000 kb, based on European population reference. The use of majority European ancestry GWAS helps to mitigate confounding due to population stratification, supporting the independence assumption (assumption 2). Finally, to avoid bias from weak instruments, we calculated the F-statistic and excluded weak genetic variants with an F-statistic <10.

#### Effect estimation and sensitivity analyses

After data preprocessing, we conducted two univariable MR (UVMR) as parts of the bidirectional MR analysis for our primary objective. These UVMRs were aimed at estimating the total effect of a single exposure on a single outcome, without conditioning on other covariates. This approach allowed us to identify all possible associations in any direction and guided multivariable analysis further.

First, we selected IVs for each CRD trait, as mentioned previously, and examined the effect of their genetically predicted liability on each CVD trait. Our primary method for effect estimation was the inverse-variance weighted (IVW) approach. To address the exclusion restriction assumption (assumption 3), particularly the risk of horizontal pleiotropy, we applied multiple sensitivity analyses using robust MR models. These analyses were included to account for different assumptions of valid IVs and the potential of heterogeneity and pleiotropy, including MR-Egger, median-based methods, MR-pleiotropy residual sum and outlier (MR-PRESSO) and contamination mixture (CM) method. If more than half of the IVs are valid, then the weighted median (WM) method provides a valid MR estimate. MR-Egger is robust to pleiotropy under the Instrument Strength Independent of Direct Effect (InSIDE) assumption. Yet, MR-Egger is also sensitive to outliers and violations of InSIDE assumption. The assumption states that the association between the genetic IV and exposure is not correlated with the (pleiotropic) path from the genetic IV to the outcome that is independent of the exposure of interest.^15 16^ We also employed the MR-PRESSO method, which enables the detection and adjustment for horizontal pleiotropy through outlier removal.^17^ Finally, we also included the CM model which groups instruments with similar MR effect estimates that may represent different mechanisms by which the risk factor impacts the outcome. Consequently, the CM model provides robust inference in the presence of up to 40% invalid IVs.^18^

Once we completed the first leg of the analysis, we conducted a second UVMR analysis, in which we reselected IVs to represent CVDs as the exposure trait and examined the effect of their genetically predicted liability on each CRD trait. The estimation methods were the same, including IVW, MR-Egger, WM, MR-PRESSO and CM methods.

As both CRD and CVD have smoking as a common risk factor, we conducted another MR analysis involving lifetime smoking as the exposure on each of CRD and CVD traits. The statistical methods employed here were the same as previously explained for bidirectional UVMR analysis.

For clarity, we referred to relationships as nominally significant if they met the p<0.05 level without controlling for multiple testing. We did not adjust for multiple testing in UVMR, because the aim of our exploratory study was to detect any potential relationships between CRD and CVD traits, keeping in mind the potential of false positive findings.

### Multivariable MR

Given that UVMR analyses showed an association between CRD and CVD, we applied multivariable MR (MVMR) to examine whether this association persisted after we accounted for smoking. Our MVMR model was not intended to assess mediation, but rather only to account for smoking as a shared risk factor. This approach allowed us to estimate the direct effect of the primary exposure independent of smoking-related pathways, while minimising model complexity and avoiding overfitting. Therefore, MVMR served as a pleiotropy adjustment, capturing potential horizontal pleiotropy by including SNPs from another exposure.^19^

For this MVMR model, genetic instruments were reselected solely from the primary exposure of interest (either CRD or CVD) at genome-wide significance (p<5×10⁻⁸). However, we did not select instruments specifically for lifetime smoking as the secondary exposure, representing a potential pleiotropic pathway. After preprocessing, that is, extracting for all genetic instruments the genetic associations with all exposures and the outcome and harmonising effect directions to the same effect allele, we estimated the effects using multivariable IVW, and then used multivariable MR-Egger (MVMR-Egger) to investigate further the possibility of unmeasured directional pleiotropy.^19^

### Inflammation markers and CVD and CRD traits

We conducted cis-MR to investigate which inflammatory markers might be shared between CRD and CVD. Instrument selection for this analysis focuses on a cis-regulatory region of each biomarker to ensure the instruments selected have biological relevance. The IV selection process has been detailed in Yarmolinsky *et al*.^20^ We summarised their IV selection process below.

The authors have systematically identified 218 inflammatory biomarkers including acute phase proteins, chemokines, growth factors, interferons, interleukins and tumour necrosis factors and mapped them using UniProt ID. They then searched the GWAS Catalog and bioRxiv for GWAS of circulating proteins. A total of six GWAS of circulating inflammatory markers met their inclusion criteria and were incorporated into their meta-analysis. Circulating protein levels in each GWAS were measured by either BioRad Bioplex assays, Olink or SomaScan, as described in the original studies. After quality control and preprocessing, Yarmolinsky *et al* obtained 45 markers from the meta-analysis with genome-wide significant SNPs (p<5×10⁻⁸) with weak LD (r²<0.10) located within or ±250 kb window. They also added 21 markers with at least 1 cis-acting genome-wide significant (p<5.0×10^−8^) variants from a single study, developing genetic instruments for a total of 66 inflammatory markers, which were subsequently used in the cis-MR.^20^

The estimation of effect for cis-MR was as follows: if only a single IV was available, we used the Wald ratio to estimate the causal effect of the inflammatory marker; otherwise, we used the IVW as the main effect estimation method. We conducted sensitivity analyses accordingly, only when multiple independent IVs were available. Sensitivity analyses included MR-Egger, WM, MR-PRESSO and CM.

We took into account multiple testing for the inflammation markers and corrected using false discovery rate (FDR) method, with q (adjusted p value)<0.05 indicating strong evidence and q-values between 0.05 and 0.20 considered suggestive.^20^ Correcting for multiple testing using the FDR provides more discovery power while still controlling the expected proportion of false positives.

### Software and packages

All analyses are conducted in R software V.4.3.2. We used the following packages for the analysis: ‘ieugwasr’ V.0.1.5,^21^ ‘MendelianRandomization’ V.0.9.0^22^ and ‘MR-PRESSO’ V.1.0.^17^

We reported this study according to the Strengthening the Reporting of Observational Studies in Epidemiology using MR guidelines.

## Results

### Instrument variables

For COPD, the number of IVs varied from 279 to 303, while for asthma, the number of IVs ranged from 138 to 146. All SNPs selected for COPD or asthma as exposure showed F-statistics greater than 10, indicating robust genetic instruments. Similarly, for CVD, each IV had an F-statistic higher than 10. The number of SNPs varied across traits, from 23 IVs for IS to 177 for CAD. The number of variables and respective F-statistics was presented in more detail in the forest plot and online supplemental tables 1–6. For cis-MR, the number of IVs varied among 65 inflammation markers is presented in online supplemental tables 7 and 8. Data for one marker (C-type lectin domain family 11 member A) were unavailable.

### How does the genetically predicted liability to COPD affect CVD?

**Figure 1:**
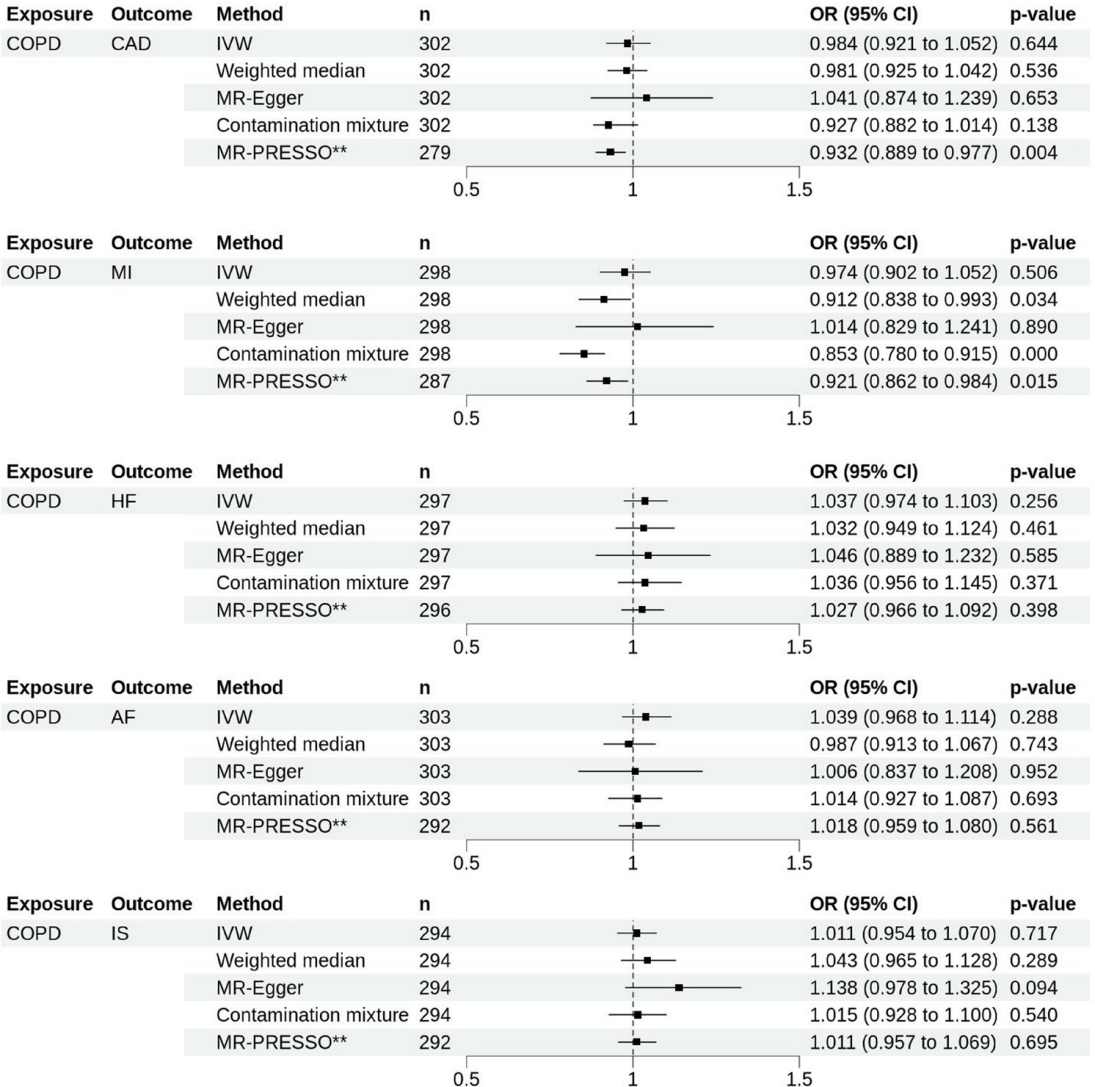
Forest plots showing the results of MR analyses on the effect of genetically predicted liability to COPD on the risk of cardiovascular diseases. Estimates are shown as OR. Horizontal lines represent the 95% CIs. **Indicates corrected MR-PRESSO estimates. AF, atrial fibrillation; CAD, coronary artery disease; COPD, chronic obstructive pulmonary disease; HF, heart failure; IS, ischaemia stroke; IVW, inverse-variance weighted; MI, myocardial infarction; MR, Mendelian randomisation; n, number of SNPs used as instrument variables in each method; PRESSO, pleiotropy residual sum and outlier; SNPs, single-nucleotide polymorphisms.

Using the IVW method, we found no evidence that genetically predicted liability to COPD affects the risk of developing any CVD (CAD: OR_IVW_ 0.984, 95% CI 0.921 to 1.052, MI: OR_IVW_ 0.974, 95% CI 0.902 to 1.052, HF: OR_IVW_ 1.037, 95% CI 0.974 to 1.103, AF: OR_IVW_ 1.039, 95% CI 0.968 to 1.114, IS: OR_IVW_ 1.011, 95% CI 0.954 to 1.070) (figure 1 and online supplemental figure 1). However, there was some inconsistency between genetically predicted liability to COPD and the risk of MI, CAD and AF across sensitivity analyses. Specifically, while the IVW and MR-Egger found no significant association, the WM and CM methods suggested a potential decreased risk of MI associated with genetic liability to COPD. After removing 11 outliers identified by MR-PRESSO, the corrected IVW estimates supported a modest association between genetic liability to COPD and reduced risk of MI and CAD. All pairs were observed to have substantial heterogeneity, as shown by a high Cochrane’s Q test and p-het<0.000 (online supplemental table 1).

### How does the genetically predicted liability to asthma affect CVD?

**Figure 2:**
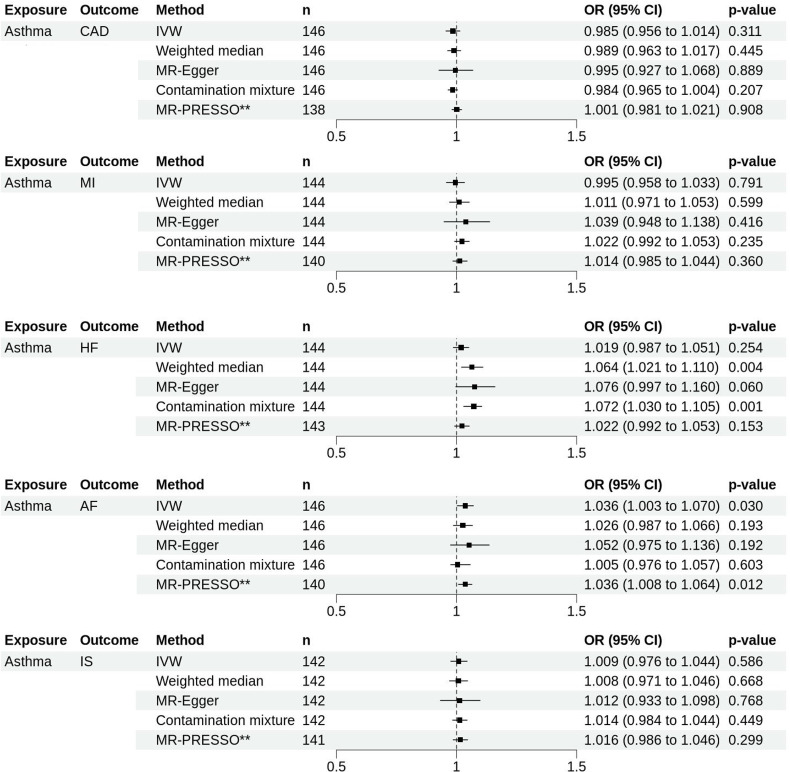
Forest plots showing the results of MR analyses on the effect of genetically predicted liability to asthma on the risk of cardiovascular diseases. Estimates are shown as OR. Horizontal lines represent the 95% CIs. **Indicates corrected MR-PRESSO estimates. AF, atrial fibrillation; CAD, coronary artery disease; HF, heart failure; IS, ischaemic stroke; IVW, inverse-variance weighted; MI, myocardial infarction; MR, Mendelian randomisation; n, number of SNPs used as instrument variables in each method; PRESSO, pleiotropy residual sum and outlier; SNPs, single-nucleotide polymorphisms.

We also examined how genetically predicted liability to asthma related to CVDs (figure 2 and online supplemental figure 2). The IVW analysis suggested a modest association between genetically predicted liability to asthma and increased odds of AF (OR_IVW_ 1.036, 95% CI 1.003 to 1.070). Nevertheless, while WM, MR-Egger and CM did not show any significant association, MR-PRESSO supported the IVW finding after removing six potential outliers (OR_MR-PRESSO_ 1.036, 95% CI 1.008 to 1.064, P_CORRECTED_=0.012). Additionally, WM and CM methods showed suggestive evidence on the association of genetically predicted liability to asthma and increased risk of HF. Only one SNP was identified as an outlier by MR-PRESSO and removing it still did not indicate an association. Across analyses, consistent direction of estimates showed a lack of horizontal pleiotropy genetically predicted liability to asthma and the risk of HF and AF, supported by P_egger_intercept_. However, both noted the significantly high Cochrane’s Q test with p-het<0.000 (online supplemental table 2).

### How does the genetically predicted liability to CVD affect COPD or asthma?

We did not identify any significant evidence supporting an effect of genetically predicted liability to any CVD on the risk of developing COPD or asthma using IVW analysis (online supplemental figures 3–6 and tables 3 and 4). Sensitivity analyses, including WM, MR-Egger and CM methods, consistently reflected these findings.

### How does the genetically predicted lifetime smoking index affect each CRD and CVD traits?

**Figure 3:**
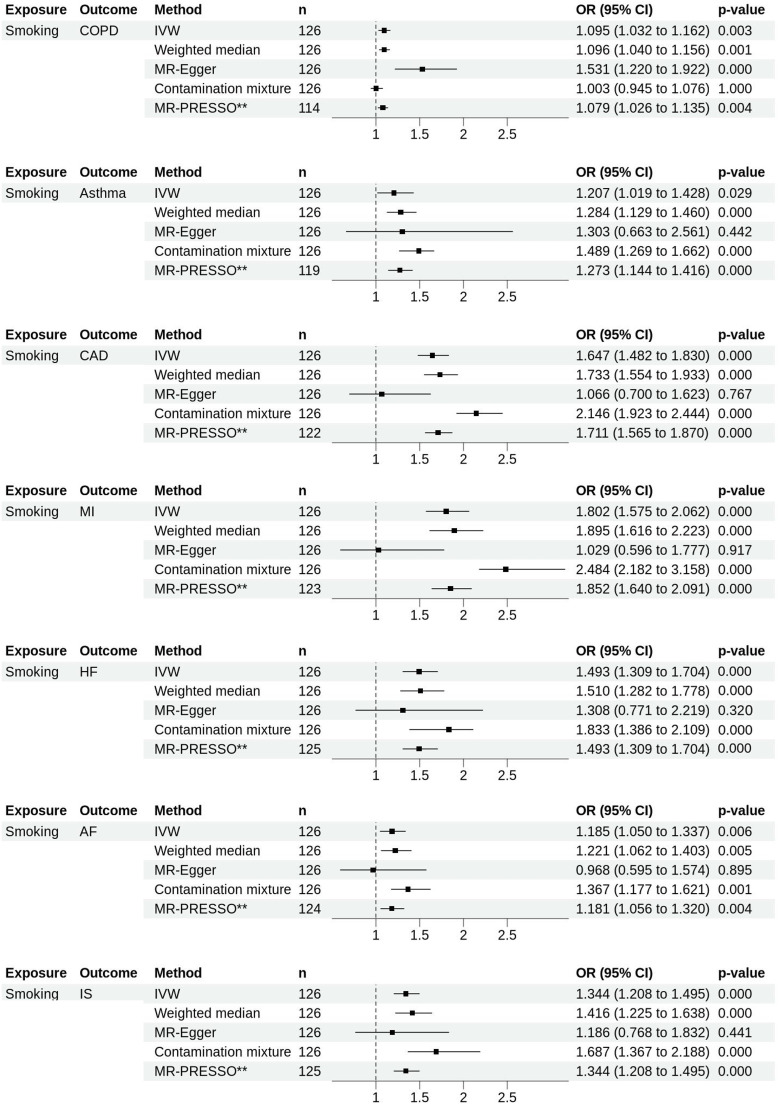
Forest plots showing the results of MR analyses on the effect of genetically predicted lifetime smoking on the risk of developing chronic respiratory diseases and cardiovascular diseases. Estimates are shown as OR. Horizontal lines represent the 95% CIs. **Indicates corrected MR-PRESSO estimates. AF, atrial fibrillation; CAD, coronary artery disease; COPD, chronic obstructive pulmonary disease; HF, heart failure; IS, ischaemic stroke; IVW, inverse-variance weighted; MI, myocardial infarction; MR, Mendelian randomisation; n, number of SNPs used as instrument variables in each method; PRESSO, pleiotropy residual sum and outlier; SNPs, single-nucleotide polymorphisms.

The IVW analysis indicated strong evidence on the association between genetically predicted lifetime smoking index and increased COPD odds by 9.5% per 1 SD (OR_IVW_ 1.095, 95% CI 1.032 to 1.162, P_IVW_=0.003) and moderate evidence for increased asthma odds by 21% (OR_IVW_ 1.207, 95% CI 1.019 to 1.428, P_IVW_=0.029). Our IVW analysis also revealed that genetically predicted lifetime smoking was associated with increased odds of all types of CVD included (figure 3 and online supplemental table 7). Sensitivity analyses showed consistent directions, though P_egger_intercept_ revealed the possibility of horizontal pleiotropy on the effect of lifetime smoking index on COPD, CAD and MI (online supplemental table 5). MR-PRESSO identified several potential outliers, and on those SNPs removal, the result still showed a strong genetic association between smoking and COPD, asthma and all CVDs.

### MVMR analysis

**Figure 4:**
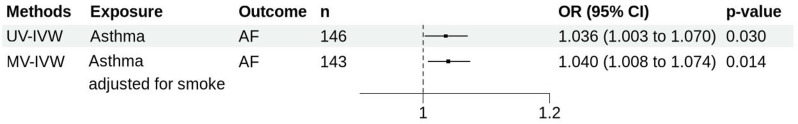
Comparison of univariable and multivariable Mendelian randomisation analysis results of the effect of genetically predicted liability to asthma towards AF adjusted for lifetime smoking. Estimates are shown as OR. Horizontal lines represent the 95% CI. AF, atrial fibrillation; MVIVW, multivariable inverse-variance weighted; n, number of SNPs used as instrument variables in each method; SNPs, single-nucleotide polymorphisms; UVIVW, univariable IVW.

Considering the close relationship between lifetime smoking towards COPD and asthma previously, we extended the analysis to account for lifetime smoking as a common risk factor in MVMR for COPD towards MI, and asthma towards AF and HF. From these MVMR analyses, we found that the effect of asthma on HF and COPD on MI was weakened when adjusted for lifetime smoking (online supplemental table 6). Meanwhile, the effect of asthma on AF was stronger when conditioning on smoking (figure 4). We did not detect significant horizontal pleiotropy, as indicated by the intercept of the MVMR-Egger model. In conclusion, our MVMR analysis provides evidence that when accounting for smoking, there was no genetic evidence that asthma or COPD may affect the risk of developing CVDs, except AF.

### How do the genetically proxied inflammatory markers affect CRD and CVD?

**Figure 5:**
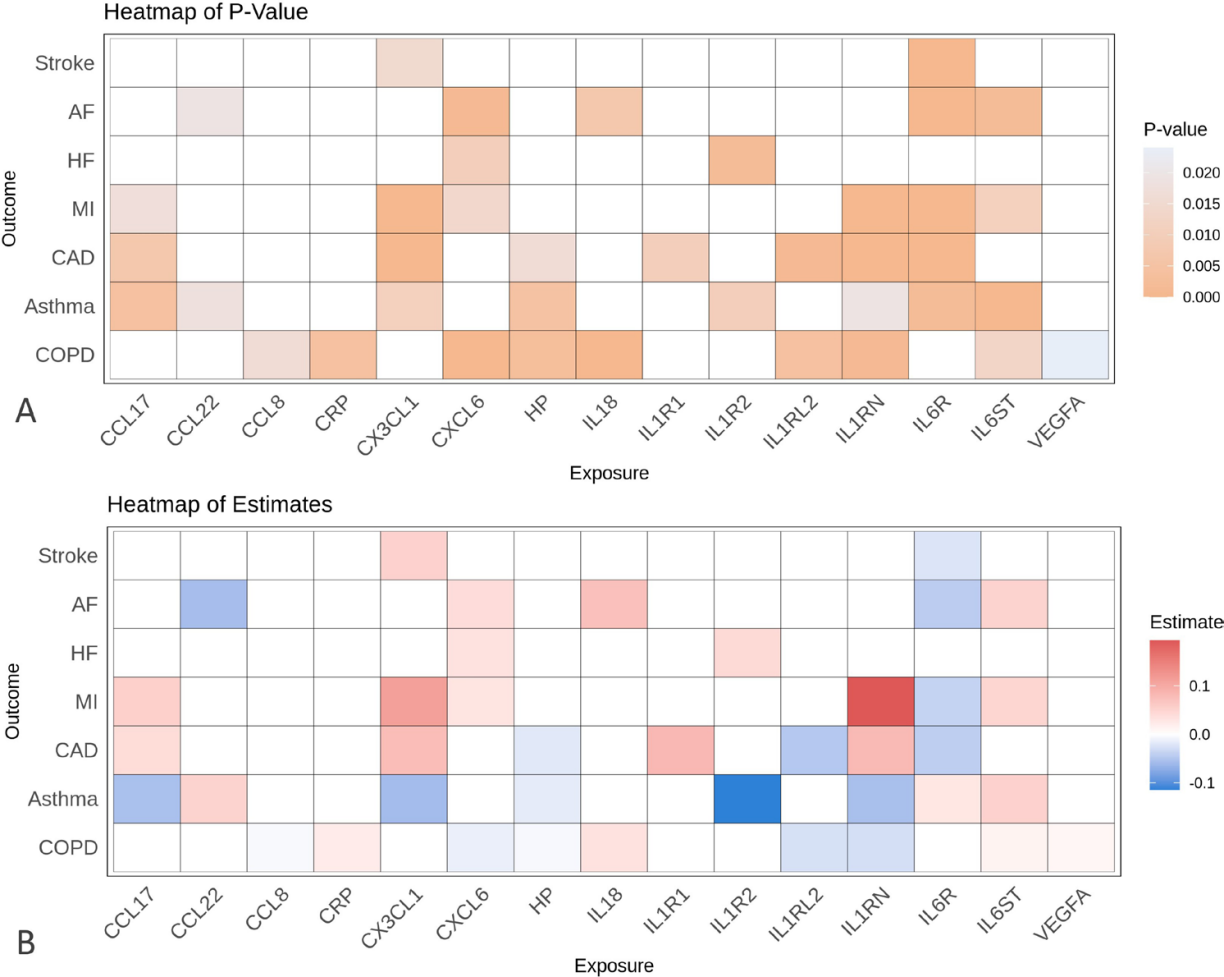
Heatmap of cis-MR analysis results of the effect of genetically predicted inflammatory markers on chronic respiratory disease and cardiovascular disease, based on (**A**) p value from IVW MR analysis and (**B**) the respective effect estimate. AF, atrial fibrillation; CAD, coronary artery disease; CCL, CC chemokine ligand; CXCL, CXC chemokine ligand; HF, heart failure; HP, haptoglobin; IL, interleukin; IL6R, IL6 receptor; IL6ST, IL6 cytokine family signal transducer; IL1RN, IL1 receptor antagonist; IL1RL2, IL1 receptor-like 2; IL1R2, IL1 receptor type 2; IS, ischaemia stroke; IVW, inverse-variance weighted; MI, myocardial infarction; MR, Mendelian randomisation.

Based on IVW results, we found 12 genetically proxied cytokines and CRD pairs and 24 genetically proxied cytokines and CVD pairs with strong evidence of an association after accounting for multiple testing adjustment. Among these, we found several inflammatory markers were shared between CRD and CVD, as presented in figure 5 and online supplemental table 7.

Two markers, IL6 receptor subunit alpha (IL6R) and IL1 receptor antagonist (IL1RN), showed significant results. The results showed strong genetic evidence (q-value<0.05) that IL6R concentration was associated with decreased risk of most CVDs including CAD (OR_IVW_ 0.958, 95% CI 0.947 to 0.968), MI (OR_IVW_ 0.963, 95% CI 0.949 to 0.976), AF (OR_IVW_ 0.957, 95% CI 0.943 to 0.971) and IS (OR_IVW_ 0.977, 95% CI 0.965 to 0.989), but increased odds of asthma (OR_IVW_ 1.029, 95% CI 1.010 to 1.049). Meanwhile, we found genetically predicted IL1RN concentration to be strongly associated with increased odds of CAD (OR_IVW_ 1.087, 95% CI 1.046 to 1.129) and MI (OR_IVW_ 1.214, 95% CI 1.142 to 1.291) and decreased odds of COPD (OR_IVW_ 0.972, 95% CI 0.955 to 0.989) and weakly associated with decreased odds of asthma. Sensitivity methods for all pairs showed consistent direction of estimate, further supported by the MR-Egger p-intercept >0.05, indicating no horizontal pleiotropy.

Other inflammatory markers with strong evidence of association shared between CVD and CRD in IVW analysis included CXCL6 and IL6ST. Moreover, there were some inflammatory markers showing mixed strong and suggestive evidence overlaps between CRD and CVD. These included IL1RL2, CX3CL1, IL1R2, HP and IL18. Additional forest plots for these overlapping inflammatory markers are shown in online supplemental figure 8 and table 8.

## Discussion

Few MR studies have used large GWAS to investigate the bidirectional effects and associations between CRD and CVD. We found no strong genetic evidence to support associations between genetically predicted liability to CRD and the risk of developing CVD. Similarly, we did not find significant genetic evidence indicating that CVD liability increases the risk of COPD or asthma. However, we confirmed that genetic liability to lifetime smoking strongly affects the risk of both CRD and CVD. Furthermore, out of 65 inflammatory markers analysed, we identified several with strong genetic associations related to both CRD and CVD.

We have explicitly not performed a multiple testing correction for the bidirectional analysis of CRD and CVD with the aim to detect as many putative associations as possible. One nominally significant association detected was between genetically predicted liability to asthma and an increased risk of AF. Yet, this effect was weak and certainly would not withstand multiple testing corrections. This finding is consistent with a previous study by Chen *et al*^23^ who also reported diminished genetic associations between asthma and AF, as well as asthma and HF, after correction. In contrast, another study found stronger evidence for a significant relationship between genetically predicted liability to asthma and AF, even after Bonferroni correction.^24^ These conflicting results may potentially be due to differences in the GWAS summary statistics used and IV selection. Both studies used a smaller-scale asthma GWAS, with the latter applying weaker genome-wide significance thresholds for IV selection (p<5×10^−6^ and 5×10^−5^). In contrast, our study used a larger and more recent GWAS with a conventional genome-wide significance threshold (p<5×10⁻⁸). We believe these differences enhance the reliability of our MR estimates.

Our studies on COPD and CVD presented both supporting and conflicting findings depending on the CVD outcome. Contrary to our finding, two separate MR studies found that genetic liability to COPD increases the risk of developing HF. The first study, which used FinnGen Consortium GWAS data for both their exposure and outcome, found evidence of a bidirectional association, where genetically predicted liability to HF affected COPD and vice versa. Notably, they also supported our findings of no genetic evidence of COPD affecting CAD, ischemic heart disease, AF or IS.^25^ The second study also examined COPD and HF, using FinnGen data for COPD, but the same HF summary statistics as our study. Interestingly, they found a significant genetic relationship between COPD and HF without evidence of reverse causation from HF to COPD.^26^ While both studies employed robust sensitivity analyses, their reliance on FinnGen alone may limit generalisability due to fewer COPD cases. Once again, our study benefited from broader GWAS resources based on a meta-analysis of many different cohorts, which should provide better estimation.

Two reasons may explain the lack of strong evidence of genetic association between CRD and CVD in our study. First, the wide range of phenotypes may obscure true associations. Our findings of significant heterogeneity and possibility of pleiotropy suggested that by including COPD and asthma as general diagnoses, we may have overlooked multiple subtypes that could involve genetic heterogeneity. Such heterogeneity could influence disease risk, pathophysiology, or responses to environmental factors. For example, in a study of former smokers, four different COPD subtypes showed variation in symptoms despite lung function severity.^27^ Another example, if onset is considered, a study observed that only childhood-onset asthma showed genetic evidence of increased CVD risk.^28^

Second, there might be no direct pathway for each CRD and CVD pairs. This absence might instead suggest that these conditions share a common underlying mechanism, such as smoking. Our findings support this hypothesis by showing that genetically predicted lifetime smoking increases the risk of various CVDs as well as COPD and asthma, as reported previously.^29^,^31^ Here, we only included lifetime smoking index as it is itself a comprehensive measure incorporating various factors such as smoking status (current, former or never), age at initiation and cessation, number of cigarettes smoked per day, smoking duration and time since cessation.^32^ Therefore, we believe it effectively reflects the impact of other smoking characteristics on disease outcomes. Nevertheless, recent work has shown that the genetic variants for lifetime smoking might be associated not only with phenotypes plausibly caused by smoking, but also with a wide range of other traits that are unlikely to be caused by smoking in the UK Biobank.^33^ Therefore, our findings should be interpreted with this in mind. Including more detailed smoking metrics may provide further insights.

Our analysis of inflammation provides new puzzle pieces into the underlying mechanisms of CRD and CVD comorbidity. As mentioned, a central hypothesis is that systemic inflammation mediates this overlap. Among all inflammatory markers assessed in cis-MR, IL6R, IL6ST and IL1RN showed the strongest, most consistent and statistically robust genetic associations. Interestingly, our finding showed contrasting direction of effect for IL6R and IL1RN on CRD and CVD, highlighting a biologically intriguing pattern that warrants detailed discussion.

We observed that genetically proxied IL6R concentrations were associated with a decreased risk of CVD but an increased risk of asthma. Our findings align with studies showing that higher genetically proxied sIL6R levels are linked to a modestly increased risk of asthma and related phenotypes.^34^ Specifically, the IL6R Asp358Ala variant (rs2228145), which increases sIL6R levels, has been reported to be associated with a higher risk of asthma, but not COPD.^35 36^ Conversely, a different SNP in the same Asp358Ala variant has been reported to reduce the risk of coronary heart disease.^37^ To complicate our findings, our analysis showed that higher genetically predicted IL6ST concentrations were linked to an increased risk of both AF and asthma.

The complexity of IL6 signalling may explain these differing effects. The IL6 receptor is composed of two key subunits: the alpha subunit, encoded by the IL6R gene, and the beta subunit, also known as membrane glycoprotein 130 (gp130) or IL6ST, encoded by the IL6ST gene. In classic signalling, IL6 attaches to the membrane-bound IL6R (mIL6R) and IL6ST on hepatocytes and immune cells, leading to anti-inflammatory and homeostatic effects. Conversely, in trans-signalling, IL6 binds to the soluble form of IL6R (sIL6R) and attaches to IL6ST on the surface of a broader range of cells that typically do not respond to IL6, triggering pro-inflammatory responses.^36 38^ However, IL6ST itself is shared among other cytokines. Therefore, the dual nature of IL6 signalling, both classical and trans-signalling pathways, along with the shared role of gp130, might explain the complex distinct direction of inflammatory markers association with different diseases.

Meanwhile, our analysis also associated genetically predicted IL1RN concentration with increased risk of CAD and MI but also with reduced risk of COPD and asthma. The result is consistent with one study linking an IL1RN variant to higher IL1Ra levels, low density lipoprotein and triglycerides, and eventually, increased CVD risk. Nevertheless, this study itself needs to be interpreted carefully due to counterintuitive results.^39^ To further confirm, another study observed genetically predicted IL1Ra levels were positively associated with CAD and cardioembolic stroke.^40^ While we have not found a previous MR study on IL1RN and COPD, it seems a meta-analysis of observational studies suggested that polymorphism in IL1RN allele 2 may be associated with an increased risk of COPD.^41^

In addition to IL6R and IL1RN, our broader cis-MR analysis revealed a similar pattern in which several inflammatory markers exhibited opposing directions of effect across CRD and CVD outcomes, that is, the same cytokine was associated with increased risk in one disease group while appearing protective in the other. These findings highlight the complexity of the genetic and inflammatory roles underlying CRD-CVD comorbidity. To better confirm whether these findings may reflect true biological complexity or methodological artefacts, future studies are warranted and should extend inflammation profiling beyond blood to include lung and cardiovascular tissues or also include additional colocalisation analysis to support the cis-MR analysis. A deeper understanding of the inflammatory profile would help clarify reports of varying treatment effects, especially regarding the cardiovascular protective benefits of corticosteroid therapy in COPD and asthma patients.

Our study has some strengths. First, MR design offers a unique perspective on the relationship between CRD and CVD comorbidity using genetic evidence minimising unknown confounding factors. Second, our use of various sensitivity analyses, including the CM method, ensures robustness under different IV assumptions. Third, the broader disease definitions used in some GWAS substantially increased participant and case numbers, thereby improving the statistical power of our analyses. Furthermore, our use of many inflammatory markers from a meta-analysis across six studies provided stronger instruments for our analysis.

Apart from the cost of reduced specificity due to broader definition for conditions with multiple and complex phenotypes and our sole use of lifetime smoking we mentioned earlier, we acknowledge other limitations. There is the potential for sample overlap due to the inclusion of summary statistics from different studies that included UK Biobank. Still, the risk has been minimised as we excluded weak instruments with F-statistic <10. Further, while MR mitigates confounding and reverse causation, it remains vulnerable to selection bias, especially due to the possibility of non-random participation in underlying GWAS cohorts. We encourage readers to also bear in mind the possibility of competing risks, as individuals who die early from one disease may not live long enough to develop the other disease.

In addition, some may consider the misclassification bias risk inherited from the use of external GWAS source. To mitigate this, the present study used COPD summary statistics from a GWAS enhanced by machine learning on spirograms to identify COPD status. Employing machine learning on spirograms may provide a more objective diagnosis than relying solely on patient records. Additionally, while the GWAS included were adjusted for sex, the summary statistics were not sex-stratified, which may limit detection of sex-specific effects. Finally, most of the GWAS were conducted in individuals of European ancestry, which may limit the generalisability of our findings to other ancestries.

In conclusion, we did not find genetic evidence to support an association directly between CRD and CVD in either directionality, except for a weakly and nominally significant genetic association between genetic liability to asthma and AF. Our findings confirmed the role of smoking as an established common risk factor contributing to both CRD and CVD. Additionally, we identified several overlapping inflammatory markers that were associated with both CRD and CVD. This suggests that the associations we observed in observational and clinical studies may not be due to one disease causing the other and may instead be partially explained by these shared factors. Further work is necessary to extend our findings and to understand better the overlapping conditions. This includes other epidemiology study designs to study non-inheritable exposures, such as air pollution, lifestyle or sociocultural background.

## Supplementary material

10.1136/thorax-2024-222908 online supplemental file 1

10.1136/thorax-2024-222908 online supplemental file 2

## Data Availability

Data are available in a public, open access repository. Data may be obtained from a third party and are not publicly available.
