# Supplementary material for: Understanding the bidirectional relationship between chronic respiratory disease and cardiovascular disease using genetic evidence
Source: Thorax. 2025 Nov 15;81(5):e222908. doi: 10.1136/thorax-2024-222908 (PMC13151528; doi:10.1136/thorax-2024-222908)
Supplement: online supplemental file 1 [file thorax-81-5-s001.pdf]

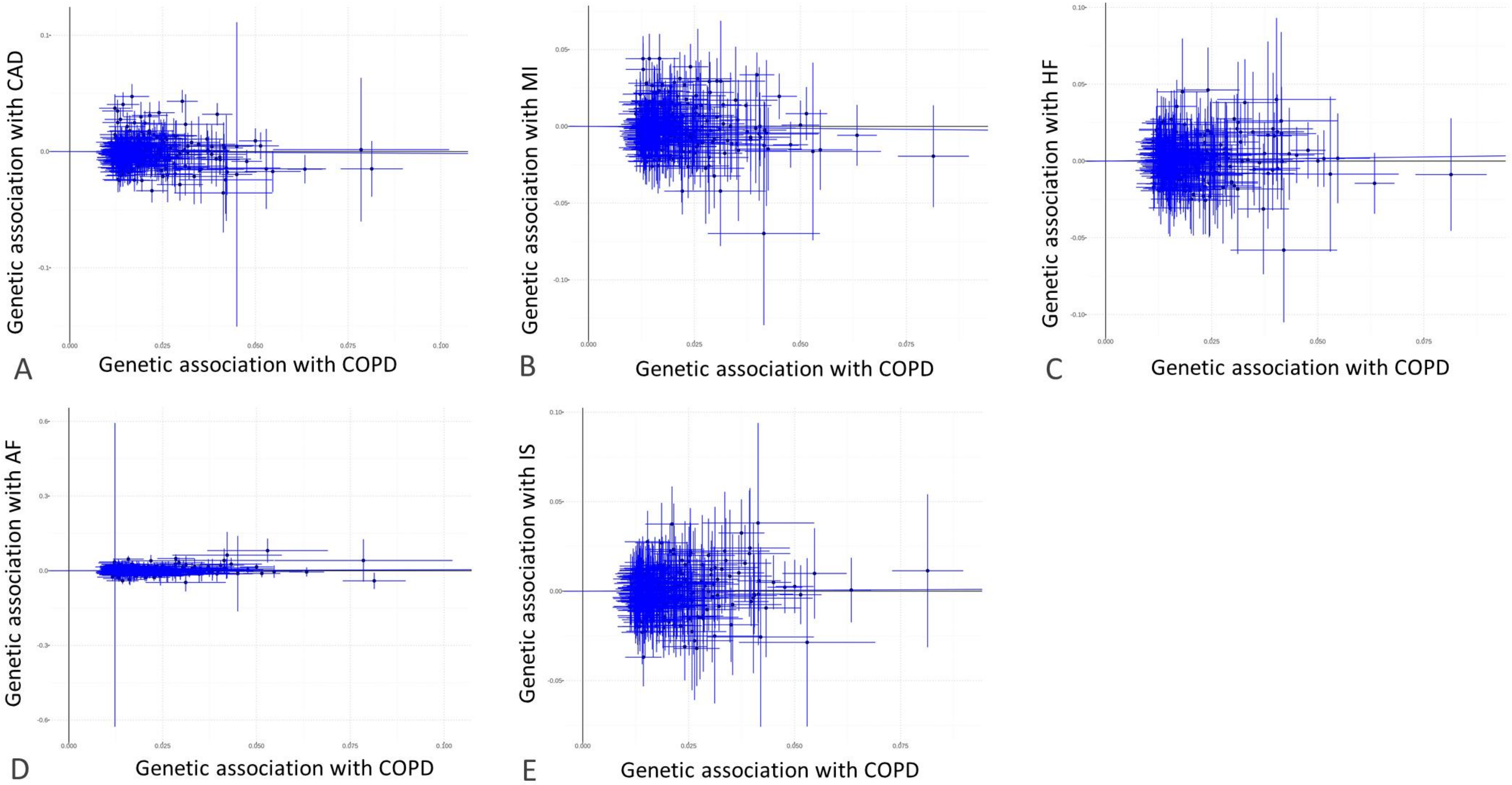

Supplement Figure 1. Scatter plots for MR analyses of the genetic associations between COPD as the exposure (horizontal axis) and (A) CAD, (B) MI (C) HF (D) AF (E) IS as the outcome (vertical axis). Each data point represents a genetic variant used as an instrument variable. Error bars are 95% confidence intervals. The estimate of MR effect from inverse variance weighted (IVW) method is shown as the slope of the regression fit through zero. AF: atrial fibrillation, CAD: coronary artery disease, COPD: chronic obstructive pulmonary diseases, HF: heart failure, IS: ischemia stroke, MI: myocardial infarction

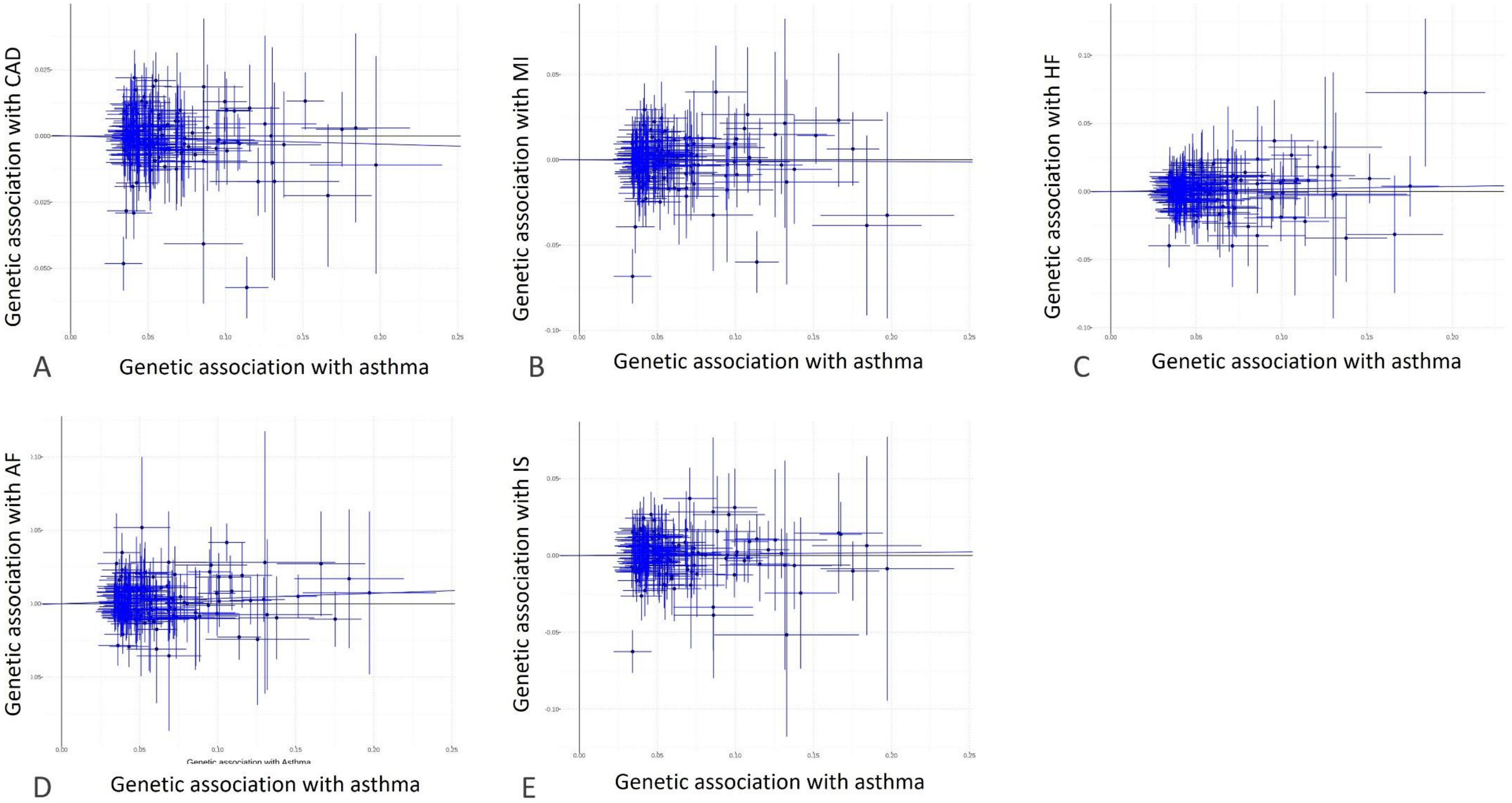

Supplement Figure 2. Scatter plots for MR analyses of the genetic associations between asthma as the exposure (horizontal axis) and (A) CAD (B) MI (C) HF (D) AF (E) IS as the outcome (vertical axis). Each data point represents a genetic variant used as an instrument variable. Error bars are 95% confidence intervals. The estimate of MR effect from inverse variance weighted (IVW) method is shown as the slope of the regression fit through zero. AF: atrial fibrillation CAD: coronary artery disease, HF: heart failure, IS: ischemia stroke, MI: myocardial infarction.

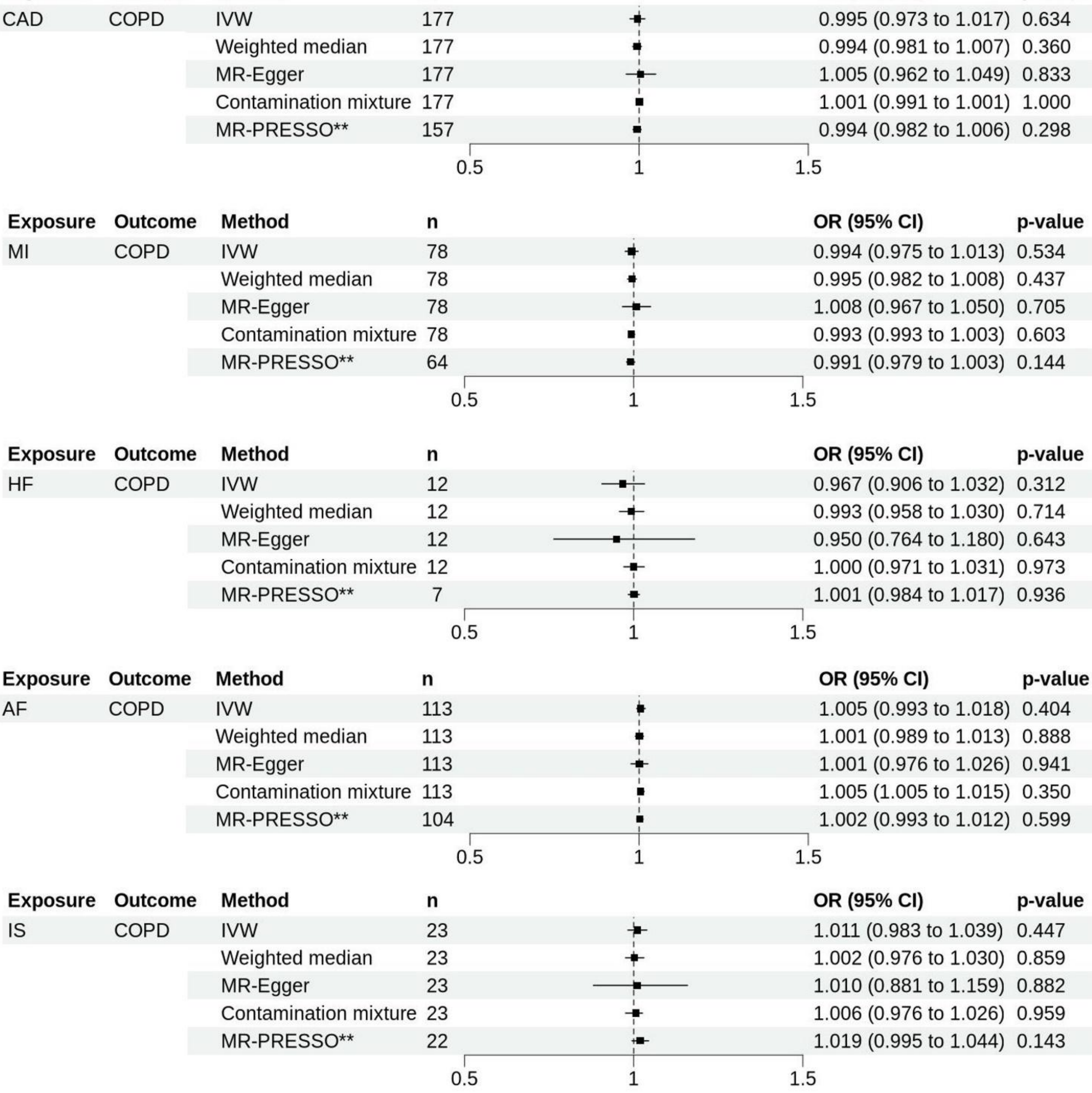

Exposure	Outcome	Method	n	OR (95% CI)	p-value
CAD	COPD	IVW	177	0.995 (0.973 to 1.017)	0.634
		Weighted median	177	0.994 (0.981 to 1.007)	0.360
		MR-Egger	177	1.005 (0.962 to 1.049)	0.833
		Contamination mixture	177	1.001 (0.991 to 1.001)	1.000
		MR-PRESSO**	157	0.994 (0.982 to 1.006)	0.298

Exposure	Outcome	Method	n	OR (95% CI)	p-value
MI	COPD	IVW	78	0.994 (0.975 to 1.013)	0.534
		Weighted median	78	0.995 (0.982 to 1.008)	0.437
		MR-Egger	78	1.008 (0.967 to 1.050)	0.705
		Contamination mixture	78	0.993 (0.993 to 1.003)	0.603
		MR-PRESSO**	64	0.991 (0.979 to 1.003)	0.144

Exposure	Outcome	Method	n	OR (95% CI)	p-value
HF	COPD	IVW	12	0.967 (0.906 to 1.032)	0.312
		Weighted median	12	0.993 (0.958 to 1.030)	0.714
		MR-Egger	12	0.950 (0.764 to 1.180)	0.643
		Contamination mixture	12	1.000 (0.971 to 1.031)	0.973
		MR-PRESSO**	7	1.001 (0.984 to 1.017)	0.936

Exposure	Outcome	Method	n	OR (95% CI)	p-value
AF	COPD	IVW	113	1.005 (0.993 to 1.018)	0.404
		Weighted median	113	1.001 (0.989 to 1.013)	0.888
		MR-Egger	113	1.001 (0.976 to 1.026)	0.941
		Contamination mixture	113	1.005 (1.005 to 1.015)	0.350
		MR-PRESSO**	104	1.002 (0.993 to 1.012)	0.599

Exposure	Outcome	Method	n	OR (95% CI)	p-value
IS	COPD	IVW	23	1.011 (0.983 to 1.039)	0.447
		Weighted median	23	1.002 (0.976 to 1.030)	0.859
		MR-Egger	23	1.010 (0.881 to 1.159)	0.882
		Contamination mixture	23	1.006 (0.976 to 1.026)	0.959
		MR-PRESSO**	22	1.019 (0.995 to 1.044)	0.143

Supplement Figure 3. Forest plots showing the results of MR analyses on the effect of genetically predicted liability to cardiovascular diseases on the risk of developing COPD. Estimates are shown as OR. Horizontal lines represent the 95% CIs. AF: atrial fibrillation, CAD: coronary artery disease, COPD: chronic obstructive pulmonary disease, HF: heart failure, IS: ischemia stroke, MI: myocardial infarction, n: number of SNPs used as instrument variables in each method, SNPs single nucleotide polymorphism, CI confidence intervals, OR odds ratio. ** indicates corrected MR-PRESSO estimates

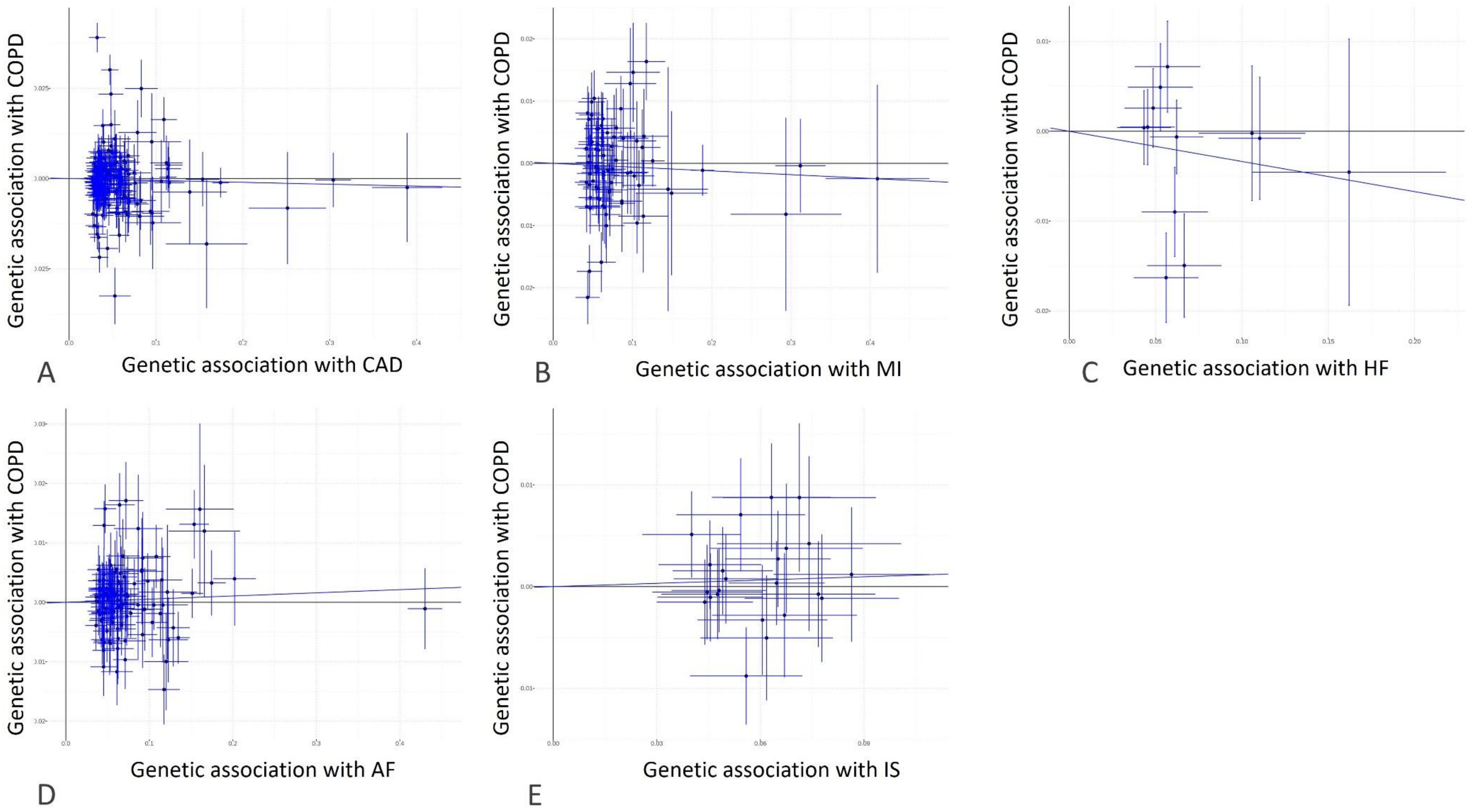

Supplement Figure 4. Scatter plots for MR analyses of the genetic associations between (A) CAD (B) MI (C) HF (D) AF (E) IS as the exposures (horizontal axis) towards COPD as the outcome (vertical axis). Each data point represents a genetic variant used as an instrument variable. Error bars are 95% confidence intervals. The estimate of MR effect from inverse variance weighted (IVW) method is shown as the slope of the regression fit through zero. AF: atrial fibrillation CAD: coronary artery disease, COPD: chronic obstructive pulmonary disease, HF: heart failure, IS: ischemia stroke, MI: myocardial infarction.

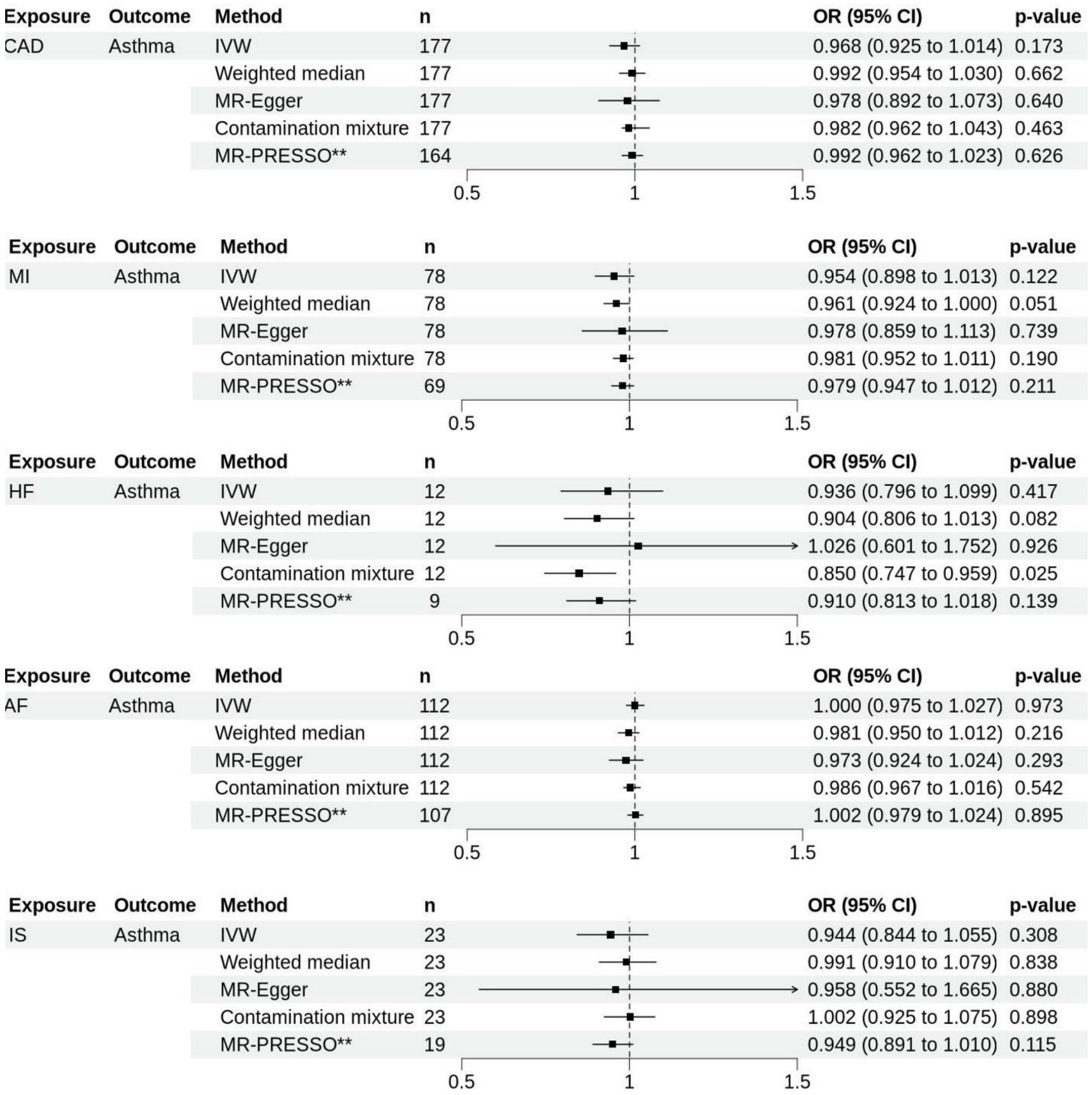

Supplement Figure 5. Forest plots showing the results of MR analyses on the effect of genetically predicted liability to cardiovascular diseases on the risk of asthma. Estimates are shown as OR. Horizontal lines represent the 95% CIs. AF: atrial fibrillation, CAD: coronary artery disease, HF: heart failure, IS: ischemia stroke, MI: myocardial infarction, n: number of SNPs used as instrument variables in each method, SNPs single nucleotide polymorphism, CI confidence intervals, OR odds ratio. ** indicates corrected MR-PRESSO estimates

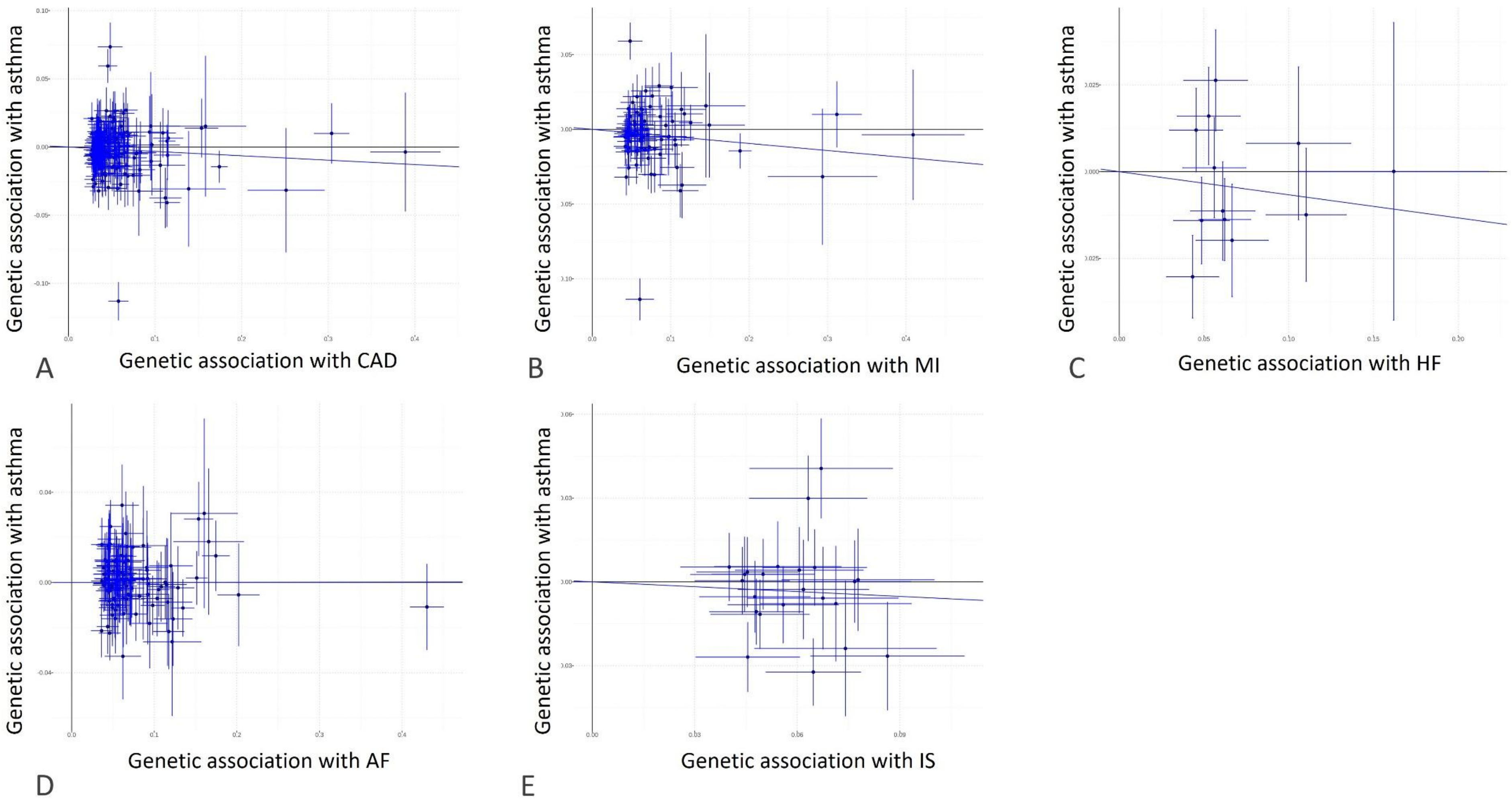

Supplement Figure 6. Scatter plots for MR analyses of the genetic associations between (A) CAD (B) MI (C) HF (D) AF (E) IS as the exposures towards asthma as the outcome (vertical axis). Each data point represents a genetic variant used as an instrument variable. Error bars are 95% confidence intervals. The estimate of MR effect from inverse variance weighted (IVW) method is shown as the slope of the regression fit through zero. AF: atrial fibrillation, CAD: coronary artery disease, HF: heart failure, IS: ischemia stroke, MI: myocardial infarction

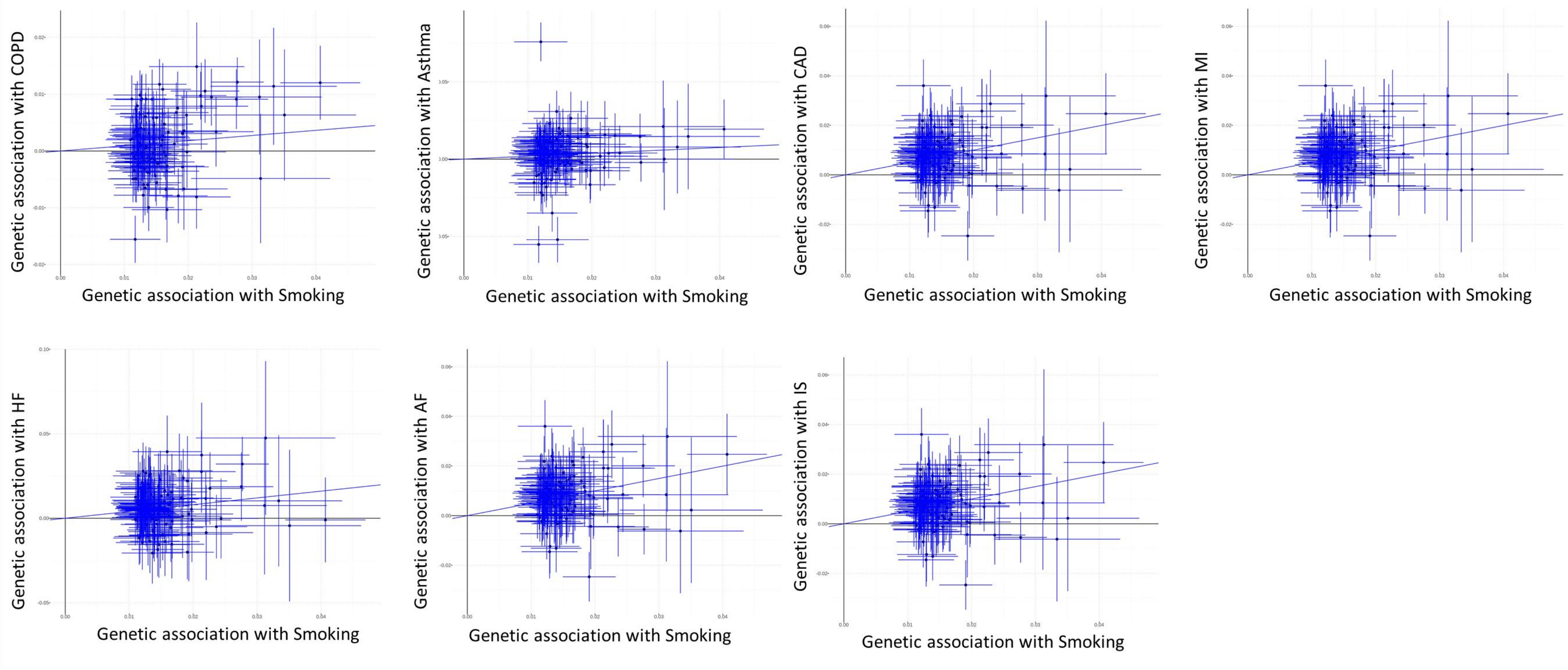

Supplement Figure 7. Scatter plots for MR analyses of the genetic associations between lifetime smoking as the exposure (horizontal axis) and CRD and CVD as the outcome (vertical axis). Each data point represents a genetic variant used as an instrument variable. Error bars are 95% confidence intervals. The estimate of MR effect from inverse variance weighted (IVW) method is shown as the slope of the regression fit through zero. AF: atrial fibrillation, CAD: coronary artery disease, COPD: chronic obstructive pulmonary disease, HF: heart failure, IS: ischemia stroke, MI: myocardial infarction

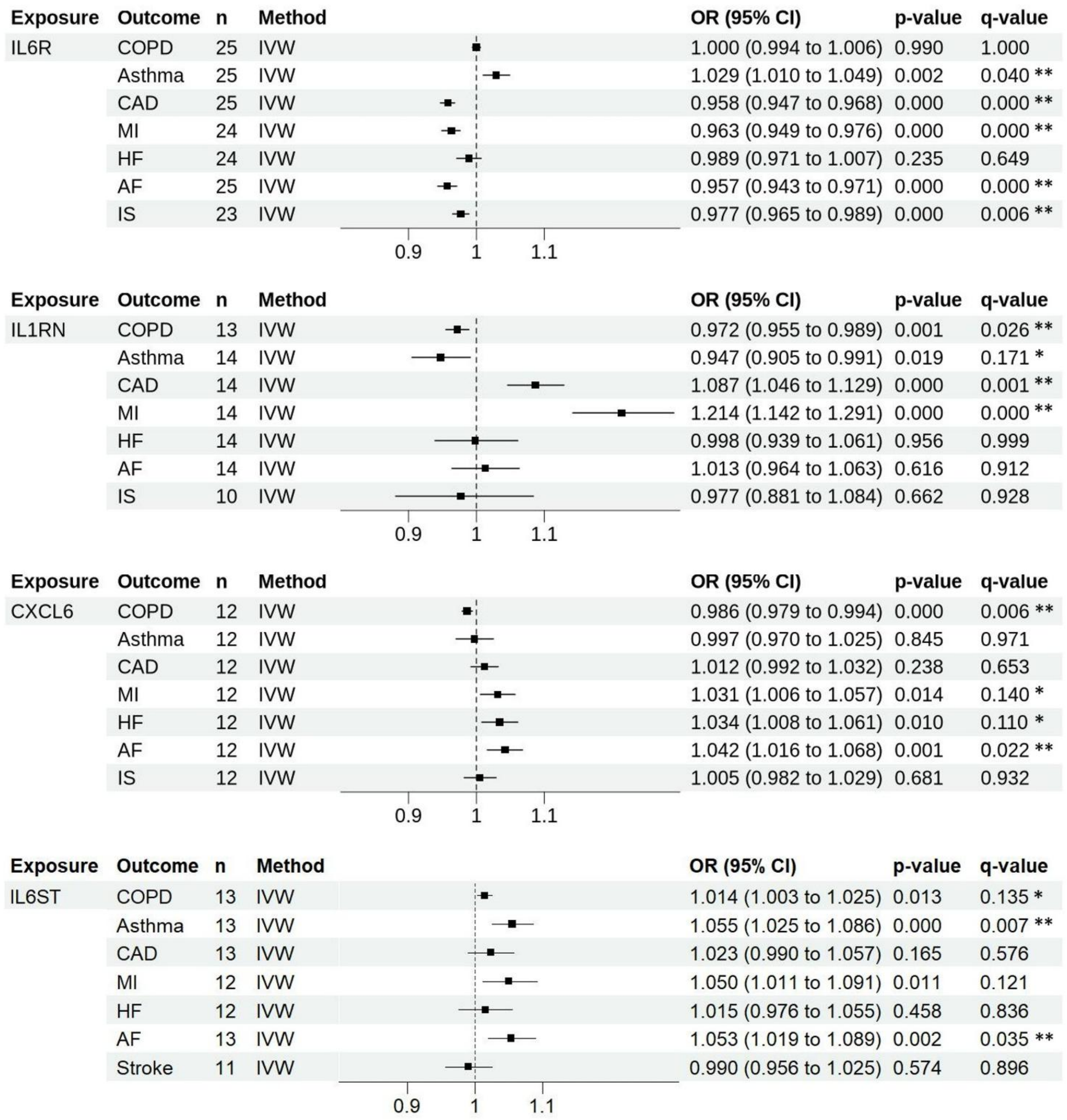

Supplement Figure 8. Forest plot showing the result of cis-MR analysis on the effect of genetically predicted inflammatory markers associations found to be overlapped in both CRD and CVD. * denotes suggestive evidence (0.05 < q-value ≤ 0.2) ** denotes strong evidence (q-value <0.05). Horizontal lines represent the 95% CIs. AF: atrial fibrillation, CAD: coronary artery disease, COPD: chronic obstructive pulmonary disease, HF: heart failure, IL: interleukin, IL6R: IL6 Receptor, IL6ST: IL6 Cytokine Family Signal Transducer, IL1RN: IL1 receptor antagonist, IL1RL2: IL1 receptor-like 2, IL1R2: IL1 Receptor Type 2, IS: ischemia stroke, MI: myocardial infarction, n: number of SNPs used as instrument variables in each method, SNPs single nucleotide polymorphism, CI confidence intervals, OR odds ratio

Exposure	Outcome	n	Method	OR (95% CI)	p-value	q-value
IL1R2	COPD	14	IVW	0.982 (0.965 to 0.998)	0.032	0.233
	Asthma	14	IVW	0.892 (0.818 to 0.973)	0.010	0.110*
	CAD	12	IVW	0.980 (0.962 to 0.999)	0.043	0.284
	MI	11	IVW	0.977 (0.944 to 1.011)	0.184	0.603
	HF	11	IVW	1.045 (1.017 to 1.074)	0.002	0.029**
	AF	12	IVW	0.990 (0.967 to 1.013)	0.401	0.800
	IS	11	IVW	1.016 (0.989 to 1.044)	0.246	0.659

0.9 1 1.1

Exposure	Outcome	n	Method	OR (95% CI)	p-value	q-value
IL1RL2	COPD	15	IVW	0.975 (0.957 to 0.992)	0.004	0.062*
	Asthma	15	IVW	0.932 (0.857 to 1.014)	0.104	0.462
	CAD	15	IVW	0.953 (0.927 to 0.980)	0.001	0.017**
	MI	15	IVW	0.961 (0.924 to 0.998)	0.041	0.276
	HF	15	IVW	1.005 (0.971 to 1.039)	0.794	0.971
	AF	16	IVW	0.996 (0.967 to 1.026)	0.800	0.971
	IS	15	IVW	0.999 (0.969 to 1.029)	0.933	0.993

0.9 1 1.1

Exposure	Outcome	n	Method	OR (95% CI)	p-value	q-value
HP	COPD	13	IVW	0.994 (0.990 to 0.998)	0.003	0.048**
	Asthma	13	IVW	0.984 (0.972 to 0.995)	0.005	0.068*
	CAD	12	IVW	0.981 (0.965 to 0.996)	0.016	0.159*
	MI	12	IVW	0.981 (0.957 to 1.006)	0.134	0.521
	HF	12	IVW	1.004 (0.991 to 1.017)	0.517	0.872
	AF	12	IVW	1.002 (0.991 to 1.013)	0.713	0.943
	IS	12	IVW	0.987 (0.975 to 0.999)	0.031	0.231

0.9 1 1.1

Exposure	Outcome	n	Method	OR (95% CI)	p-value	q-value
IL18	COPD	6	IVW	1.035 (1.019 to 1.051)	0.000	0.001 **
	Asthma	6	IVW	1.010 (0.965 to 1.058)	0.660	0.928
	CAD	6	IVW	0.996 (0.960 to 1.033)	0.833	0.971
	MI	6	IVW	1.011 (0.955 to 1.069)	0.711	0.943
	HF	6	IVW	1.040 (0.982 to 1.102)	0.183	0.603
	AF	6	IVW	1.078 (1.021 to 1.137)	0.007	0.083 *
	IS	5	IVW	0.983 (0.929 to 1.040)	0.552	0.893

0.9 1 1.1

Exposure	Outcome	n	Method	OR (95% CI)	p-value	q-value
CX3CL1	COPD	8	IVW	0.988 (0.976 to 1.000)	0.058	0.333
	Asthma	8	IVW	0.943 (0.901 to 0.987)	0.011	0.118
	CAD	8	IVW	1.081 (1.049 to 1.113)	0.000	0.000 **
	MI	8	IVW	1.120 (1.071 to 1.172)	0.000	0.000 **
	HF	8	IVW	1.032 (0.979 to 1.088)	0.242	0.657
	AF	8	IVW	1.020 (0.975 to 1.067)	0.386	0.786
	IS	8	IVW	1.055 (1.010 to 1.102)	0.015	0.152

0.9 1 1.1

Supplement Figure 8. cont. Forest plot showing the result of cis-MR analysis on the effect of genetically predicted inflammatory markers associations found to be overlapped in both CRD and CVD. * denotes suggestive evidence (0.05 < q-value ≤ 0.2) ** denotes strong evidence (q-value <0.05). Horizontal lines represent the 95% CIs. AF: atrial fibrillation, CAD: coronary artery disease,

COPD: chronic obstructive pulmonary disease, HF: heart failure, IL: interleukin, IL6R: IL6 Receptor, IL6ST: IL6 Cytokine Family Signal Transducer, IL1RN: IL1 receptor antagonist, IL1RL2: IL1 receptor-like 2, IL1R2: IL1 Receptor Type 2, IS: ischemia stroke, MI: myocardial infarction, n: number of SNPs used as instrument variables in each method, SNPs single nucleotide polymorphism, CI confidence intervals, OR odds ratio

Supplement Table 1. Mendelian Randomization analysis result for the effect of chronic obstructive pulmonary disease on cardiovascular diseases

Exposure	Outcome	Methods	n	Estimate	betalow	betahi	OR	ORlow	ORhi	p-value	Heterogeneity test statistic (Cochran's Q)	df	I^2	F-statistic	P-het value	$P_{egger\_intercept}$
COPD	CAD	IVW	302	-0.016	-0.082	0.051	0.984	0.921	1.052	0.644	1283.238	301	76.5%	72.7	0.000	
		Weighted median	302	-0.019	-0.078	0.041	0.981	0.925	1.042	0.536						
		MR-Egger	302	0.040	-0.134	0.214	1.041	0.874	1.239	0.653						0.498
		Contamination mixture	302	-0.076	-0.126	0.014	0.927	0.882	1.014	0.138						
		MR-PRESSO	279	-0.070	-0.117	-0.023	0.932	0.889	0.977	0.004						
COPD	MI	IVW	298	-0.026	-0.103	0.051	0.974	0.902	1.052	0.506	744.5619	297	60.1%	71.8	0.000	
		Weighted median	298	-0.092	-0.177	-0.007	0.912	0.838	0.993	0.034						
		MR-Egger	298	0.014	-0.187	0.216	1.014	0.829	1.241	0.890						0.672
		Contamination mixture	298	-0.159	-0.249	-0.089	0.853	0.780	0.915	0.000						
		MR-PRESSO	287	-0.082	-0.148	-0.016	0.921	0.862	0.984	0.015						
COPD	HF	IVW	297	0.036	-0.026	0.098	1.037	0.974	1.103	0.256	413.055	296	28.3%	71.5	0.000	
		Weighted median	297	0.032	-0.053	0.117	1.032	0.949	1.124	0.461						
		MR-Egger	297	0.045	-0.118	0.209	1.046	0.889	1.232	0.585						0.901
		Contamination mixture	297	0.035	-0.045	0.135	1.036	0.956	1.145	0.371						
		MR-PRESSO	296	0.027	-0.035	0.088	1.027	0.966	1.092	0.398						
COPD	AF	IVW	303	0.038	-0.032	0.108	1.039	0.968	1.114	0.288	751.299	302	59.8%	72.4	0.000	
		Weighted median	303	-0.013	-0.091	0.065	0.987	0.913	1.067	0.743						
		MR-Egger	303	0.006	-0.178	0.189	1.006	0.837	1.208	0.952						0.711
		Contamination mixture	303	0.014	-0.076	0.084	1.014	0.927	1.087	0.693						
		MR-PRESSO	292	0.018	-0.042	0.077	1.018	0.959	1.080	0.561						
COPD	IS	IVW	294	0.011	-0.047	0.068	1.011	0.954	1.070	0.717	439.141	293	33.3%	71.8	0.000	
		Weighted median	294	0.042	-0.036	0.120	1.043	0.965	1.128	0.289						
		MR-Egger	294	0.130	-0.022	0.281	1.138	0.978	1.325	0.094						0.097
		Contamination mixture	294	0.015	-0.075	0.095	1.015	0.928	1.100	0.540						
		MR-PRESSO	292	0.011	-0.044	0.067	1.011	0.957	1.069	0.695						

betalow: 95% lower CI for the estimate, betahi: 95% upper CI for the estimate.

ORlow: 95% lower CI for the OR, ORhi: 95% upper CI for the OR.

AF: atrial fibrillation, CAD: coronary artery disease, COPD: chronic obstructive pulmonary diseases, HF: heart failure, IS: ischemia stroke, MI: myocardial infarction, n: number of SNPs used as instrument variables in each method, SNPs single nucleotide polymorphism, CI confidence intervals, OR odds ratio.

*** indicates corrected MR-PRESSO estimates*

Supplement Table 2. Mendelian Randomization analysis result for the effect of asthma on cardiovascular disease

Exposure	Outcome	Methods	n	Estimate	betalow	betahi	OR	ORlow	ORhi	p-value	Heterogeneity test statistic (Cochran's Q)	df	I^2	F-statistic	P-het value	$P_{egger\_intercept}$
Asthma	CAD	IVW	146	-0.015	-0.045	0.014	0.985	0.956	1.014	0.311	497.874	145	70.9%	74.4	0.000	
		Weighted median	146	-0.011	-0.038	0.017	0.989	0.963	1.017	0.445						
		MR-Egger	146	-0.005	-0.076	0.066	0.995	0.927	1.068	0.889						0.756
		Contamination mixture	146	-0.016	-0.036	0.004	0.984	0.965	1.004	0.207						
		MR-PRESSO	138	0.001	-0.019	0.021	1.001	0.981	1.021	0.908						
Asthma	MI	IVW	144	-0.005	-0.043	0.033	0.995	0.958	1.033	0.791	346.010	143	58.7%	72.8	0.000	
		Weighted median	144	0.011	-0.030	0.051	1.011	0.971	1.053	0.599						
		MR-Egger	144	0.038	-0.053	0.129	1.039	0.948	1.138	0.416						0.310
		Contamination mixture	144	0.022	-0.008	0.052	1.022	0.992	1.053	0.235						
		MR-PRESSO	140	0.014	-0.016	0.043	1.014	0.985	1.044	0.360						
Asthma	HF	IVW	144	0.018	-0.013	0.050	1.019	0.987	1.051	0.254	220.246	143	35.1%	74.7	0.000	
		Weighted median	144	0.062	0.020	0.105	1.064	1.021	1.110	0.004						
		MR-Egger	144	0.073	-0.003	0.149	1.076	0.997	1.160	0.060						0.122
		Contamination mixture	144	0.070	0.030	0.100	1.072	1.030	1.105	0.001						
		MR-PRESSO	143	0.022	-0.008	0.052	1.022	0.992	1.053	0.153						
Asthma	AF	IVW	146	0.035	0.003	0.068	1.036	1.003	1.070	0.030	317.592	145	54.3%	74.3	0.000	
		Weighted median	146	0.025	-0.013	0.064	1.026	0.987	1.066	0.193						
		MR-Egger	146	0.051	-0.026	0.128	1.052	0.975	1.136	0.192						0.661
		Contamination mixture	146	0.005	-0.025	0.055	1.005	0.976	1.057	0.603						
		MR-PRESSO	140	0.035	0.008	0.062	1.036	1.008	1.064	0.012						
Asthma	IS	IVW	142	0.009	-0.025	0.043	1.009	0.976	1.044	0.586	295.668	141	52.3%	75.8	0.000	
		Weighted median	142	0.008	-0.029	0.045	1.008	0.971	1.046	0.668						
		MR-Egger	142	0.012	-0.069	0.094	1.012	0.933	1.098	0.768						0.940
		Contamination mixture	142	0.013	-0.017	0.043	1.014	0.984	1.044	0.449						
		MR-PRESSO	141	0.016	-0.014	0.045	1.016	0.986	1.046	0.299						

betalow: 95% lower CI for the estimate; betahi: 95% upper CI for the estimate.

ORlow: 95% lower CI for the OR; ORhi: 95% upper CI for the OR.

AF: atrial fibrillation, CAD: coronary artery disease, HF: heart failure, IS: ischemia stroke, MI: myocardial infarction, n: number of SNPs used as instrument variables in each method, SNPs single nucleotide polymorphism, CI confidence intervals, OR odds ratio.

** indicates corrected MR-PRESSO estimates

Supplement Table 3. Mendelian Randomization analysis result for the effect of cardiovascular disease on chronic obstructive pulmonary disease

Exposure	Outcome	Method	n	Estimate	betalow	betahi	OR	ORlow	ORhi	p-value	Heterogeneity test statistic (Cochran's Q)	df	I^2	F-statistic	P-het value	$P_{egger\_intercept}$
CAD	COPD	IVW	177	-0.005	-0.027	0.016	0.995	0.973	1.017	0.634	1704.448	176	89.70%	76.9	0	
		Weighted median	177	-0.006	-0.020	0.007	0.994	0.981	1.007	0.360						
		MR-Egger	177	0.005	-0.038	0.048	1.005	0.962	1.049	0.833						0.602
		Contamination mixture	177	0.001	-0.009	0.001	1.001	0.991	1.001	1.000						
		MR-PRESSO**	157	-0.006	-0.018	0.006	0.994	0.982	1.006	0.298						
MI	COPD	IVW	78	-0.006	-0.025	0.013	0.994	0.975	1.013	0.534	512.379	77	85.00%	65.2	0	
		Weighted median	78	-0.005	-0.018	0.008	0.995	0.982	1.008	0.437						
		MR-Egger	78	0.008	-0.033	0.049	1.008	0.967	1.050	0.705						0.449
		Contamination mixture	78	-0.007	-0.007	0.003	0.993	0.993	1.003	0.603						
		MR-PRESSO**	64	-0.009	-0.021	0.003	0.991	0.979	1.003	0.144						
HF	COPD	IVW	12	-0.034	-0.099	0.032	0.967	0.906	1.032	0.312	85.09	11	87.10%	41.5	0	
		Weighted median	12	-0.007	-0.043	0.029	0.993	0.958	1.030	0.714						
		MR-Egger	12	-0.051	-0.269	0.166	0.950	0.764	1.180	0.643						0.866
		Contamination mixture	12	0.000	-0.030	0.030	1.000	0.971	1.031	0.973						
		MR-PRESSO**	7	0.001	-0.016	0.017	1.001	0.984	1.017	0.936						
AF	COPD	IVW	113	0.005	-0.007	0.018	1.005	0.993	1.018	0.404	458.647	112	75.60%	89.6	0	
		Weighted median	113	0.001	-0.011	0.012	1.001	0.989	1.013	0.888						
		MR-Egger	113	0.001	-0.024	0.026	1.001	0.976	1.026	0.941						0.693
		Contamination mixture	113	0.005	0.005	0.015	1.005	1.005	1.015	0.350						
		MR-PRESSO**	104	0.002	-0.007	0.012	1.002	0.993	1.012	0.599						
IS	COPD	IVW	23	0.011	-0.017	0.038	1.011	0.983	1.039	0.447	51.077	22	56.90%	44.7	0	
		Weighted median	23	0.002	-0.025	0.030	1.002	0.976	1.030	0.859						
		MR-Egger	23	0.010	-0.127	0.148	1.010	0.881	1.159	0.882						0.996
		Contamination mixture	23	0.006	-0.024	0.026	1.006	0.976	1.026	0.959						
		MR-PRESSO**	22	0.019	-0.005	0.043	1.019	0.995	1.044	0.143						

betalow: 95% lower CI for the estimate; betahi: 95% upper CI for the estimate.

ORlow: 95% lower CI for the OR; ORhi: 95% upper CI for the OR.

AF: atrial fibrillation, CAD: coronary artery disease, COPD: chronic obstructive pulmonary diseases, HF: heart failure, IS: ischemia stroke, MI: myocardial infarction, n: number of SNPs used as instrument variables in each method, SNPs single nucleotide polymorphism, CI confidence intervals, OR odds ratio.

*** indicates corrected MR-PRESSO estimates*

Supplement Table 4. Mendelian Randomization analysis result for the effect of cardiovascular disease on asthma

Exposure	Outcome	Methods	n	Estimate	betalow	betahi	OR	ORlow	ORhi	p-value	Heterogeneity test statistic (Cochran's Q)	df	I^2	F-statistic	P-het value	$P_{egger\_intercept}$
CAD	Asthma	IVW	177	-0.032	-0.078	0.014	0.968	0.925	1.014	0.173	934.867	176	81.2%	76.9	0.000	
		Weighted median	177	-0.008	-0.047	0.030	0.992	0.954	1.030	0.662						
		MR-Egger	177	-0.022	-0.114	0.070	0.978	0.892	1.073	0.640						0.805
		Contamination mixture	177	-0.018	-0.038	0.042	0.982	0.962	1.043	0.463						
		MR-PRESSO**	164	-0.008	-0.038	0.023	0.992	0.962	1.023	0.626						
MI	Asthma	IVW	78	-0.047	-0.107	0.013	0.954	0.898	1.013	0.122	606.138	77	87.3%	65.2	0.000	
		Weighted median	78	-0.039	-0.079	0.000	0.961	0.924	1.000	0.051						
		MR-Egger	78	-0.022	-0.151	0.107	0.978	0.859	1.113	0.739						0.665
		Contamination mixture	78	-0.019	-0.049	0.011	0.981	0.952	1.011	0.190						
		MR-PRESSO**	69	-0.021	-0.054	0.012	0.979	0.947	1.012	0.211						
HF	Asthma	IVW	12	-0.067	-0.228	0.094	0.936	0.796	1.099	0.417	62.767	11	82.5%	41.5	0.000	
		Weighted median	12	-0.101	-0.216	0.013	0.904	0.806	1.013	0.082						
		MR-Egger	12	0.025	-0.510	0.561	1.026	0.601	1.752	0.926						0.722
		Contamination mixture	12	-0.162	-0.292	-0.042	0.850	0.747	0.959	0.025						
		MR-PRESSO**	9	-0.095	-0.208	0.018	0.910	0.813	1.018	0.139						
AF	Asthma	IVW	112	0.000	-0.025	0.026	1.000	0.975	1.027	0.973	238.337	111	53.4%	90.0	0.000	
		Weighted median	112	-0.020	-0.051	0.012	0.981	0.950	1.012	0.216						
		MR-Egger	112	-0.028	-0.079	0.024	0.973	0.924	1.024	0.293						0.216
		Contamination mixture	112	-0.014	-0.034	0.016	0.986	0.967	1.016	0.542						
		MR-PRESSO**	107	0.002	-0.021	0.024	1.002	0.979	1.024	0.895						
IS	Asthma	IVW	23	-0.058	-0.169	0.053	0.944	0.844	1.055	0.308	99.239	22	77.8%	44.7	0.000	
		Weighted median	23	-0.009	-0.094	0.076	0.991	0.910	1.079	0.838						
		MR-Egger	23	-0.043	-0.595	0.510	0.958	0.552	1.665	0.880						0.956
		Contamination mixture	23	0.002	-0.078	0.072	1.002	0.925	1.075	0.898						
		MR-PRESSO**	19	-0.053	-0.115	0.010	0.949	0.891	1.010	0.115						

betalow: 95% lower CI for the estimate; betahi: 95% upper CI for the estimate.

ORlow: 95% lower CI for the OR; ORhi: 95% upper CI for the OR.

AF: atrial fibrillation, CAD: coronary artery disease, HF: heart failure, IS: ischemia stroke, MI: myocardial infarction, n: number of SNPs used as instrument variables in each method, SNPs single nucleotide polymorphism, CI confidence intervals, OR odds ratio.

** indicates corrected MR-PRESSO estimates

Supplement Table 5. Mendelian Randomization for the effect of lifetime smoking on chronic respiratory diseases and cardiovascular diseases

Exposure	Outcome	Methods	n	Estimate	betalow	betahi	OR	ORlow	ORhi	p-value	Heterogeneity test statistic (Cochran's Q)	df	I^2	F-statistic	P-het value	$P_{egger\_intercept}$
Smoking	COPD	IVW	126	0.091	0.032	0.150	1.095	1.032	1.162	0.003	575.8285	125	78.3%	44.1	0.000	
		Weighted median	126	0.092	0.040	0.145	1.096	1.040	1.156	0.001						
		MR-Egger	126	0.426	0.199	0.653	1.531	1.220	1.922	0.000						0.003
		Contamination mixture	126	0.003	-0.057	0.073	1.003	0.945	1.076	1.000						
		MR-PRESSO	114	0.076	0.026	0.127	1.079	1.026	1.135	0.004						
Smoking	Asthma	IVW	126	0.188	0.019	0.357	1.207	1.019	1.428	0.029	142.1496	125	12.1%	44.1	0.140	
		Weighted median	126	0.250	0.121	0.378	1.284	1.129	1.460	0.000						
		MR-Egger	126	0.265	-0.411	0.940	1.303	0.663	2.561	0.442						0.817
		Contamination mixture	126	0.398	0.238	0.508	1.489	1.269	1.662	0.000						
		MR-PRESSO	119	0.241	0.134	0.348	1.273	1.144	1.416	0.000						
Smoking	CAD	IVW	126	0.499	0.393	0.604	1.647	1.482	1.830	0.000	324.7559	125	61.5%	44.1	0.000	
		Weighted median	126	0.550	0.441	0.659	1.733	1.554	1.933	0.000						
		MR-Egger	126	0.064	-0.357	0.484	1.066	0.700	1.623	0.767						0.037
		Contamination mixture	126	0.764	0.654	0.894	2.146	1.923	2.444	0.000						
		MR-PRESSO	122	0.537	0.448	0.626	1.711	1.565	1.870	0.000						
Smoking	MI	IVW	126	0.589	0.562	0.724	1.802	1.575	2.062	0.000	230.2148	125	45.7%	44.1	0.000	
		Weighted Median	126	0.639	0.562	0.799	1.895	1.616	2.223	0.000						
		MR-Egger	126	0.029	-0.517	0.575	1.029	0.596	1.777	0.917						0.038
		Contamination mixture	126	0.910	0.780	1.150	2.484	2.181	3.158	0.000						
		MR-PRESSO**	123	0.617	0.495	0.738	1.853	1.641	2.091	0.000						
Smoking	HF	IVW	126	0.401	0.269	0.533	1.493	1.309	1.704	0.000	199.6419	125	37.4%	44.1	0.000	
		Weighted Median	126	0.412	0.248	0.576	1.510	1.282	1.778	0.000						
		MR-Egger	126	0.268	-0.260	0.797	1.308	0.771	2.219	0.320						0.611
		Contamination mixture	126	0.610	0.330	0.750	1.840	1.391	2.117	0.000						
		MR-PRESSO	126	0.401	0.269	0.533	1.493	1.309	1.704	0.000						
Smoking	AF	IVW	126	0.170	0.049	0.290	1.185	1.050	1.337	0.006	232.7816	125	46.3%	44.1	0.000	
		Weighted Median	126	0.200	0.060	0.339	1.221	1.062	1.403	0.005						
		MR-Egger	126	-0.033	-0.519	0.454	0.968	0.595	1.574	0.895						0.400
		Contamination mixture	126	0.310	0.160	0.480	1.363	1.174	1.616	0.001						
		MR-PRESSO**	125	0.153	0.038	0.268	1.165	1.039	1.307	0.010						
Smoking	IS	IVW	126	0.296	0.189	0.402	1.344	1.208	1.495	0.000	157.4129	125	20.6%	44.1	0.026	
		Weighted Median	126	0.348	0.203	0.493	1.416	1.225	1.638	0.000						
		MR-Egger	126	0.171	-0.264	0.605	1.186	0.768	1.832	0.441						0.561
		Contamination mixture	126	0.520	0.310	0.780	1.682	1.363	2.181	0.000						
		MR-PRESSO	126	0.296	0.189	0.402	1.344	1.208	1.495	0.000						

betalow: 95% lower CI for the estimate; betahi: 95% upper CI for the estimate.

ORlow: 95% lower CI for the OR; ORhi: 95% upper CI for the OR.

AF: atrial fibrillation, CAD: coronary artery disease, HF: heart failure, IS: ischemia stroke, MI: myocardial infarction, n: number of SNPs used as instrument variables in each method, SNPs single nucleotide polymorphism, CI confidence intervals, OR odds ratio.

** indicates corrected MR-PRESSO estimates

Supplement Table 6. Multivariable Mendelian Randomization adjusted for lifetime smoking.

Exposure	Outcome	Methods	n	Estimate	Std Error	95%CI		p-value	Cochran's Q	df	p-het
						lower	upper				
COPD	MI	MVIVW	293	-0.014	0.040	-0.092	0.064	0.725	718.287	291	0.000
Smoking			293	0.640	0.213	0.223	1.058	0.003			
COPD		MVEgger	293	0.019	0.103	-0.183	0.221	0.856			
Smoking			293	0.643	0.213	0.225	1.062	0.003			
(intercept)			293	-0.001	0.002	-0.005	0.003	0.730			
Asthma	AF	MVIVW	143	0.039	0.016	0.008	0.071	0.014	298.118	141	0.000
Smoking			143	0.491	0.267	-0.032	1.014	0.066			
Asthma		MVEgger	143	0.054	0.039	-0.022	0.129	0.163			
Smoking			143	0.488	0.268	-0.037	1.013	0.069			
(intercept)			143	-0.001	0.002	-0.006	0.004	0.684	297.758	140	0.000
Asthma	HF	MVIVW	143	0.025	0.016	-0.006	0.057	0.113	211.067	141	0.000
Smoking			143	0.279	0.263	-0.236	0.794	0.288			
Asthma		MVEgger	143	0.084	0.038	0.010	0.159	0.027			
Smoking			143	0.266	0.261	-0.245	0.778	0.308			
(intercept)		MVIVW	143	-0.004	0.002	-0.009	0.001	0.088	297.758	140	0.000

AF: atrial fibrillation, COPD: chronic obstructive pulmonary diseases, HF: heart failure, IS: ischemia stroke, MI: myocardial infarction, MVIVW: multivariable inverse-variance weighted, MVEgger: multivariable Egger analysis, n: number of SNPs used as instrument variables in each method, SNPs single nucleotide polymorphism, CI confidence intervals, OR odds ratio.

Supplement Table 7. Cis-Mendelian Randomization for the effect of inflammatory markers on chronic respiratory diseases and cardiovascular diseases based on Wald test/IVW method.

Exposure	Outcome	n	Estimate	StdError	CI95Lower	CI95Upper	p-value	OR	ORlow	ORhi	q-value
ADM	COPD	3	-0.019	0.015	-0.049	0.011	0.213	0.981	0.952	1.011	0.623
ADM	Asthma	3	0.000	0.044	-0.086	0.086	0.997	1.000	0.917	1.090	1.000
ADM	CAD	3	-0.005	0.037	-0.077	0.068	0.903	0.995	0.926	1.071	0.980
ADM	MI	3	0.004	0.058	-0.109	0.117	0.949	1.004	0.896	1.124	0.997
ADM	HF	3	-0.004	0.059	-0.120	0.111	0.940	0.996	0.887	1.117	0.995
ADM	AF	3	0.044	0.051	-0.055	0.143	0.384	1.045	0.946	1.154	0.786
ADM	Stroke	3	-0.010	0.054	-0.117	0.096	0.849	0.990	0.890	1.101	0.971
APCS	COPD	3	-0.007	0.007	-0.020	0.006	0.299	0.993	0.980	1.006	0.722
APCS	Asthma	3	-0.038	0.020	-0.077	0.000	0.051	0.962	0.926	1.000	0.309
APCS	CAD	3	-0.009	0.016	-0.040	0.022	0.561	0.991	0.961	1.022	0.896
APCS	MI	3	0.009	0.024	-0.037	0.055	0.706	1.009	0.963	1.057	0.943
APCS	HF	3	-0.037	0.024	-0.085	0.011	0.127	0.964	0.919	1.011	0.516
APCS	AF	3	-0.029	0.021	-0.071	0.013	0.177	0.972	0.932	1.013	0.594
APCS	Stroke	3	-0.006	0.022	-0.050	0.038	0.794	0.994	0.952	1.039	0.971
CCL11	COPD	1	0.004	0.028	-0.051	0.058	0.890	1.004	0.951	1.060	0.976
CCL11	Asthma	1	0.073	0.080	-0.083	0.229	0.360	1.076	0.920	1.257	0.767
CCL11	CAD	1	-0.011	0.065	-0.138	0.116	0.868	0.989	0.871	1.123	0.971
CCL11	MI	1	-0.009	0.099	-0.203	0.186	0.932	0.992	0.816	1.204	0.993
CCL11	HF	1	-0.080	0.105	-0.285	0.125	0.443	0.923	0.752	1.133	0.824
CCL11	AF	1	-0.045	0.087	-0.216	0.127	0.609	0.956	0.806	1.135	0.908
CCL11	Stroke	1	-0.013	0.092	-0.194	0.168	0.886	0.987	0.823	1.183	0.975
CCL14	COPD	6	0.000	0.003	-0.007	0.007	0.974	1.000	0.993	1.007	1.000
CCL14	Asthma	6	0.024	0.011	0.001	0.046	0.038	1.024	1.001	1.047	0.264
CCL14	CAD	6	0.000	0.008	-0.016	0.015	0.958	1.000	0.984	1.015	0.999
CCL14	MI	6	-0.001	0.012	-0.024	0.022	0.948	0.999	0.976	1.023	0.997
CCL14	HF	6	0.000	0.014	-0.026	0.027	0.977	1.000	0.974	1.027	1.000
CCL14	AF	6	0.005	0.014	-0.023	0.032	0.729	1.005	0.978	1.033	0.948
CCL14	Stroke	6	0.008	0.014	-0.021	0.036	0.595	1.008	0.980	1.036	0.900
CCL15	COPD	2	0.003	0.003	-0.003	0.008	0.365	1.003	0.997	1.008	0.767
CCL15	Asthma	2	-0.020	0.008	-0.036	-0.004	0.014	0.980	0.965	0.996	0.144
CCL15	CAD	2	-0.001	0.007	-0.014	0.013	0.926	0.999	0.986	1.013	0.991

Exposure	Outcome	n	Estimate	StdError	CI95Lower	CI95Upper	p-value	OR	ORlow	ORhi	q-value
CCL15	MI	2	-0.007	0.009	-0.026	0.011	0.430	0.993	0.974	1.011	0.818
CCL15	HF	2	-0.019	0.012	-0.043	0.006	0.137	0.982	0.958	1.006	0.528
CCL15	AF	2	0.015	0.010	-0.004	0.034	0.126	1.015	0.996	1.035	0.512
CCL15	Stroke	2	0.016	0.010	-0.004	0.036	0.118	1.016	0.996	1.037	0.490
CCL16	COPD	4	0.001	0.002	-0.003	0.006	0.511	1.001	0.997	1.006	0.870
CCL16	Asthma	4	0.005	0.014	-0.022	0.033	0.703	1.005	0.978	1.033	0.942
CCL16	CAD	4	-0.002	0.005	-0.012	0.008	0.712	0.998	0.988	1.008	0.943
CCL16	MI	4	-0.001	0.008	-0.017	0.014	0.858	0.999	0.983	1.014	0.971
CCL16	HF	4	0.006	0.009	-0.012	0.024	0.522	1.006	0.988	1.025	0.877
CCL16	AF	4	-0.007	0.007	-0.021	0.008	0.371	0.993	0.979	1.008	0.772
CCL16	Stroke	4	-0.002	0.008	-0.017	0.013	0.770	0.998	0.983	1.013	0.965
CCL17	COPD	3	-0.008	0.007	-0.021	0.005	0.217	0.992	0.979	1.005	0.627
CCL17	Asthma	3	-0.054	0.019	-0.091	-0.017	0.004	0.947	0.913	0.983	0.062
CCL17	CAD	3	0.042	0.015	0.012	0.072	0.007	1.043	1.012	1.075	0.081
CCL17	MI	3	0.056	0.023	0.010	0.102	0.017	1.057	1.010	1.107	0.163
CCL17	HF	3	0.016	0.025	-0.033	0.065	0.524	1.016	0.967	1.068	0.879
CCL17	AF	3	0.023	0.021	-0.018	0.064	0.281	1.023	0.982	1.066	0.704
CCL17	Stroke	3	0.030	0.023	-0.015	0.076	0.191	1.031	0.985	1.079	0.605
CCL2	COPD	2	-0.036	0.033	-0.099	0.028	0.274	0.965	0.905	1.029	0.695
CCL2	Asthma	2	-0.022	0.093	-0.204	0.160	0.813	0.978	0.815	1.174	0.971
CCL2	CAD	2	-0.034	0.074	-0.179	0.112	0.650	0.967	0.836	1.119	0.925
CCL2	MI	2	-0.020	0.113	-0.242	0.202	0.859	0.980	0.785	1.224	0.971
CCL2	HF	2	-0.152	0.120	-0.388	0.084	0.206	0.859	0.678	1.087	0.615
CCL2	AF	2	-0.051	0.102	-0.250	0.149	0.620	0.951	0.779	1.161	0.912
CCL2	Stroke	2	0.219	0.109	0.006	0.433	0.044	1.245	1.006	1.543	0.287
CCL22	COPD	3	0.017	0.008	0.001	0.032	0.032	1.017	1.001	1.033	0.233
CCL22	Asthma	3	0.052	0.022	0.009	0.095	0.018	1.053	1.009	1.100	0.167
CCL22	CAD	3	-0.031	0.018	-0.067	0.004	0.080	0.969	0.936	1.004	0.400
CCL22	MI	3	-0.040	0.026	-0.092	0.012	0.128	0.961	0.912	1.012	0.516
CCL22	HF	3	-0.012	0.029	-0.069	0.046	0.689	0.988	0.933	1.047	0.935
CCL22	AF	3	-0.057	0.025	-0.106	-0.009	0.019	0.944	0.900	0.991	0.171
CCL22	Stroke	3	-0.005	0.027	-0.059	0.048	0.851	0.995	0.943	1.050	0.971
CCL25	COPD	4	-0.005	0.003	-0.010	0.000	0.039	0.995	0.990	1.000	0.268
CCL25	Asthma	4	-0.004	0.007	-0.018	0.010	0.576	0.996	0.982	1.010	0.896

Exposure	Outcome	n	Estimate	StdError	CI95Lower	CI95Upper	p-value	OR	ORlow	ORhi	q-value
CCL25	CAD	4	0.022	0.009	0.004	0.040	0.019	1.022	1.004	1.041	0.171
CCL25	MI	4	0.034	0.009	0.015	0.052	0.000	1.034	1.015	1.054	0.010
CCL25	HF	4	0.003	0.012	-0.020	0.025	0.828	1.003	0.980	1.025	0.971
CCL25	AF	4	-0.004	0.008	-0.020	0.012	0.666	0.996	0.981	1.013	0.930
CCL25	Stroke	4	0.007	0.009	-0.010	0.024	0.430	1.007	0.990	1.024	0.818
CCL27	COPD	2	0.002	0.009	-0.016	0.020	0.799	1.002	0.985	1.020	0.971
CCL27	Asthma	2	0.003	0.026	-0.048	0.054	0.908	1.003	0.953	1.055	0.982
CCL27	CAD	2	-0.003	0.021	-0.044	0.039	0.904	0.997	0.957	1.040	0.981
CCL27	MI	2	-0.017	0.032	-0.079	0.044	0.581	0.983	0.924	1.045	0.896
CCL27	HF	2	0.019	0.034	-0.048	0.086	0.581	1.019	0.953	1.089	0.896
CCL27	AF	2	-0.033	0.029	-0.091	0.024	0.251	0.967	0.913	1.024	0.668
CCL27	Stroke	2	-0.065	0.031	-0.126	-0.005	0.035	0.937	0.882	0.995	0.245
CCL3	COPD	9	0.000	0.005	-0.011	0.010	0.963	1.000	0.989	1.011	0.999
CCL3	Asthma	10	0.019	0.026	-0.032	0.070	0.461	1.019	0.969	1.073	0.838
CCL3	CAD	10	-0.002	0.012	-0.026	0.022	0.889	0.998	0.975	1.023	0.976
CCL3	MI	10	0.009	0.019	-0.028	0.046	0.636	1.009	0.972	1.047	0.919
CCL3	HF	12	0.029	0.021	-0.013	0.071	0.179	1.029	0.987	1.073	0.597
CCL3	AF	15	0.033	0.015	0.003	0.064	0.031	1.034	1.003	1.066	0.230
CCL3	Stroke	10	0.023	0.021	-0.018	0.064	0.264	1.024	0.983	1.066	0.684
CCL4	COPD	7	-0.007	0.010	-0.026	0.012	0.457	0.993	0.975	1.012	0.836
CCL4	Asthma	9	-0.013	0.020	-0.053	0.027	0.516	0.987	0.948	1.027	0.872
CCL4	CAD	9	0.010	0.019	-0.027	0.047	0.594	1.010	0.973	1.049	0.900
CCL4	MI	7	0.038	0.042	-0.045	0.122	0.366	1.039	0.956	1.129	0.769
CCL4	HF	8	-0.018	0.061	-0.138	0.101	0.766	0.982	0.871	1.107	0.965
CCL4	AF	12	0.034	0.029	-0.023	0.092	0.245	1.035	0.977	1.096	0.658
CCL4	Stroke	9	-0.008	0.025	-0.058	0.042	0.763	0.992	0.944	1.043	0.965
CCL7	COPD	3	-0.004	0.004	-0.013	0.004	0.320	0.996	0.987	1.004	0.731
CCL7	Asthma	3	0.011	0.013	-0.014	0.036	0.395	1.011	0.986	1.037	0.796
CCL7	CAD	3	-0.002	0.011	-0.024	0.020	0.848	0.998	0.976	1.020	0.971
CCL7	MI	1	-0.007	0.019	-0.044	0.029	0.700	0.993	0.957	1.030	0.942
CCL7	HF	1	0.010	0.020	-0.030	0.050	0.631	1.010	0.970	1.051	0.916
CCL7	AF	4	0.031	0.016	-0.001	0.062	0.057	1.031	0.999	1.064	0.326
CCL7	Stroke	2	0.002	0.022	-0.041	0.046	0.927	1.002	0.959	1.047	0.992
CCL8	COPD	5	-0.005	0.002	-0.009	-0.001	0.016	0.995	0.991	0.999	0.157

Exposure	Outcome	n	Estimate	StdError	CI95Lower	CI95Upper	p-value	OR	ORlow	ORhi	q-value
CCL8	Asthma	5	0.001	0.005	-0.009	0.012	0.826	1.001	0.991	1.012	0.971
CCL8	CAD	5	0.002	0.008	-0.014	0.019	0.782	1.002	0.986	1.019	0.971
CCL8	MI	5	-0.002	0.006	-0.014	0.011	0.802	0.998	0.986	1.011	0.971
CCL8	HF	5	0.011	0.007	-0.003	0.025	0.122	1.011	0.997	1.025	0.504
CCL8	AF	5	0.009	0.006	-0.003	0.021	0.139	1.009	0.997	1.021	0.529
CCL8	Stroke	5	0.009	0.007	-0.004	0.022	0.190	1.009	0.996	1.022	0.605
CRP	COPD	5	0.024	0.008	0.008	0.041	0.004	1.025	1.008	1.042	0.055
CRP	Asthma	5	0.001	0.024	-0.046	0.048	0.974	1.001	0.955	1.049	1.000
CRP	CAD	5	-0.012	0.023	-0.058	0.033	0.597	0.988	0.944	1.034	0.901
CRP	MI	5	0.009	0.031	-0.051	0.069	0.769	1.009	0.950	1.071	0.965
CRP	HF	5	0.005	0.032	-0.057	0.068	0.868	1.005	0.944	1.070	0.971
CRP	AF	5	0.027	0.035	-0.042	0.095	0.445	1.027	0.959	1.100	0.824
CRP	Stroke	4	-0.013	0.037	-0.086	0.060	0.727	0.987	0.918	1.062	0.948
CSF1	COPD	3	0.009	0.013	-0.016	0.034	0.480	1.009	0.984	1.034	0.848
CSF1	Asthma	3	-0.016	0.036	-0.087	0.055	0.664	0.984	0.917	1.057	0.929
CSF1	CAD	3	0.176	0.030	0.117	0.234	0.000	1.192	1.125	1.263	0.000
CSF1	MI	3	0.207	0.045	0.119	0.295	0.000	1.230	1.126	1.343	0.000
CSF1	HF	3	0.129	0.047	0.036	0.222	0.007	1.137	1.036	1.248	0.083
CSF1	AF	3	0.042	0.040	-0.037	0.120	0.297	1.043	0.964	1.128	0.719
CSF1	Stroke	3	0.068	0.043	-0.017	0.152	0.115	1.070	0.984	1.165	0.487
CX3CL1	COPD	8	-0.012	0.006	-0.025	0.000	0.058	0.988	0.976	1.000	0.333
CX3CL1	Asthma	8	-0.059	0.023	-0.104	-0.014	0.011	0.943	0.901	0.987	0.118
CX3CL1	CAD	8	0.078	0.015	0.048	0.107	0.000	1.081	1.049	1.113	0.000
CX3CL1	MI	8	0.113	0.023	0.068	0.158	0.000	1.120	1.071	1.172	0.000
CX3CL1	HF	8	0.032	0.027	-0.021	0.085	0.242	1.032	0.979	1.088	0.657
CX3CL1	AF	8	0.020	0.023	-0.025	0.065	0.386	1.020	0.975	1.067	0.786
CX3CL1	Stroke	8	0.054	0.022	0.010	0.097	0.015	1.055	1.010	1.102	0.152
CXCL1	COPD	6	-0.017	0.009	-0.034	0.000	0.052	0.983	0.967	1.000	0.314
CXCL1	Asthma	6	-0.008	0.020	-0.048	0.031	0.677	0.992	0.954	1.031	0.932
CXCL1	CAD	6	-0.025	0.014	-0.053	0.003	0.080	0.975	0.948	1.003	0.399
CXCL1	MI	6	-0.002	0.022	-0.046	0.041	0.911	0.998	0.955	1.042	0.984
CXCL1	HF	6	0.032	0.024	-0.014	0.079	0.172	1.033	0.986	1.082	0.587
CXCL1	AF	6	-0.026	0.032	-0.089	0.038	0.429	0.975	0.915	1.039	0.818
CXCL1	Stroke	6	-0.012	0.021	-0.053	0.030	0.591	0.989	0.948	1.031	0.899

Exposure	Outcome	n	Estimate	StdError	CI95Lower	CI95Upper	p-value	OR	ORlow	ORhi	q-value
CXCL10	COPD	1	-0.009	0.028	-0.063	0.045	0.744	0.991	0.939	1.046	0.955
CXCL10	Asthma	1	0.041	0.080	-0.115	0.198	0.604	1.042	0.891	1.219	0.906
CXCL10	CAD	1	0.020	0.069	-0.115	0.154	0.775	1.020	0.891	1.167	0.965
CXCL10	MI	1	0.125	0.102	-0.074	0.325	0.218	1.134	0.929	1.384	0.627
CXCL10	HF	1	0.105	0.106	-0.103	0.313	0.323	1.110	0.902	1.367	0.733
CXCL10	AF	1	-0.200	0.089	-0.374	-0.026	0.024	0.819	0.688	0.974	0.198
CXCL10	Stroke	1	-0.099	0.093	-0.281	0.082	0.284	0.905	0.755	1.086	0.708
CXCL11	COPD	1	0.003	0.009	-0.015	0.021	0.739	1.003	0.985	1.022	0.953
CXCL11	Asthma	1	-0.019	0.027	-0.072	0.033	0.477	0.981	0.931	1.034	0.847
CXCL11	CAD	1	0.001	0.029	-0.055	0.057	0.967	1.001	0.946	1.059	1.000
CXCL11	AF	1	0.021	0.058	-0.093	0.136	0.714	1.022	0.911	1.145	0.944
CXCL16	COPD	9	0.006	0.008	-0.008	0.021	0.397	1.006	0.992	1.021	0.798
CXCL16	Asthma	9	-0.042	0.022	-0.084	0.001	0.055	0.959	0.919	1.001	0.321
CXCL16	CAD	9	0.108	0.021	0.067	0.150	0.000	1.114	1.069	1.161	0.000
CXCL16	MI	9	0.102	0.029	0.046	0.158	0.000	1.107	1.047	1.171	0.010
CXCL16	HF	9	0.054	0.042	-0.027	0.136	0.192	1.056	0.973	1.145	0.605
CXCL16	AF	9	0.096	0.035	0.027	0.166	0.007	1.101	1.027	1.180	0.081
CXCL16	Stroke	9	0.029	0.035	-0.041	0.098	0.419	1.029	0.960	1.103	0.812
CXCL6	COPD	12	-0.014	0.004	-0.021	-0.007	0.000	0.986	0.979	0.994	0.006
CXCL6	Asthma	12	-0.003	0.014	-0.030	0.025	0.845	0.997	0.970	1.025	0.971
CXCL6	CAD	12	0.012	0.010	-0.008	0.032	0.238	1.012	0.992	1.032	0.653
CXCL6	MI	12	0.031	0.013	0.006	0.055	0.014	1.031	1.006	1.057	0.140
CXCL6	HF	12	0.034	0.013	0.008	0.059	0.010	1.034	1.008	1.061	0.110
CXCL6	AF	12	0.041	0.013	0.016	0.065	0.001	1.042	1.016	1.068	0.022
CXCL6	Stroke	12	0.005	0.012	-0.018	0.028	0.681	1.005	0.982	1.029	0.932
CXCL8	COPD	1	0.018	0.028	-0.036	0.072	0.508	1.018	0.965	1.075	0.870
CXCL8	Asthma	1	0.141	0.077	-0.010	0.291	0.067	1.151	0.990	1.337	0.359
CXCL8	CAD	1	0.045	0.061	-0.074	0.164	0.456	1.046	0.929	1.178	0.835
CXCL8	MI	1	0.180	0.087	0.009	0.350	0.039	1.197	1.009	1.419	0.265
CXCL8	HF	1	-0.213	0.112	-0.431	0.006	0.056	0.808	0.650	1.006	0.324
CXCL8	AF	1	-0.168	0.092	-0.348	0.012	0.067	0.845	0.706	1.012	0.359
CXCL8	Stroke	1	0.013	0.100	-0.182	0.208	0.899	1.013	0.833	1.231	0.980
CXCL9	COPD	1	0.005	0.016	-0.026	0.035	0.759	1.005	0.975	1.036	0.964
CXCL9	Asthma	1	0.006	0.045	-0.082	0.094	0.898	1.006	0.921	1.099	0.980

Exposure	Outcome	n	Estimate	StdError	CI95Lower	CI95Upper	p-value	OR	ORlow	ORhi	q-value
CXCL9	CAD	1	0.071	0.038	-0.003	0.145	0.060	1.074	0.997	1.156	0.337
CXCL9	MI	1	0.007	0.055	-0.102	0.115	0.900	1.007	0.903	1.122	0.980
CXCL9	HF	1	-0.049	0.058	-0.164	0.065	0.397	0.952	0.849	1.067	0.798
CXCL9	AF	1	0.059	0.049	-0.038	0.156	0.232	1.061	0.963	1.169	0.645
CXCL9	Stroke	1	0.080	0.051	-0.020	0.181	0.118	1.084	0.980	1.198	0.491
F2	COPD	1	0.025	0.021	-0.016	0.066	0.236	1.025	0.984	1.069	0.650
F2	Asthma	1	0.071	0.061	-0.048	0.191	0.240	1.074	0.953	1.210	0.655
F2	CAD	1	0.152	0.050	0.054	0.251	0.002	1.165	1.056	1.285	0.040
F2	MI	1	0.293	0.077	0.142	0.443	0.000	1.340	1.153	1.558	0.005
F2	HF	1	0.069	0.079	-0.086	0.225	0.382	1.072	0.917	1.252	0.783
F2	AF	1	-0.002	0.067	-0.134	0.130	0.976	0.998	0.875	1.139	1.000
F2	Stroke	1	0.297	0.073	0.154	0.441	0.000	1.346	1.166	1.555	0.002
FGF23	COPD	2	0.033	0.022	-0.010	0.077	0.134	1.034	0.990	1.080	0.521
FGF23	Asthma	2	-0.015	0.064	-0.141	0.111	0.820	0.985	0.869	1.118	0.971
FGF23	CAD	2	0.071	0.055	-0.036	0.179	0.191	1.074	0.965	1.196	0.605
FGF23	MI	2	0.082	0.080	-0.076	0.240	0.309	1.085	0.927	1.271	0.727
FGF23	HF	2	-0.146	0.085	-0.312	0.020	0.084	0.864	0.732	1.020	0.409
FGF23	AF	2	-0.121	0.072	-0.263	0.020	0.093	0.886	0.769	1.020	0.439
FGF23	Stroke	2	-0.075	0.086	-0.243	0.093	0.381	0.928	0.785	1.097	0.782
FGF7	Asthma	1	-0.049	0.034	-0.117	0.018	0.151	0.952	0.890	1.018	0.550
FGF7	CAD	1	0.032	0.028	-0.023	0.087	0.252	1.033	0.977	1.091	0.669
FGF7	MI	1	0.064	0.043	-0.019	0.148	0.132	1.066	0.981	1.159	0.521
FGF7	HF	1	0.079	0.045	-0.008	0.167	0.075	1.083	0.992	1.181	0.382
FGF7	AF	1	0.126	0.038	0.051	0.201	0.001	1.135	1.053	1.223	0.020
FGF7	Stroke	1	0.036	0.040	-0.042	0.114	0.368	1.037	0.959	1.121	0.769
FGG	COPD	2	-0.013	0.013	-0.038	0.013	0.344	0.988	0.962	1.014	0.752
FGG	Asthma	2	0.020	0.038	-0.055	0.095	0.603	1.020	0.947	1.099	0.906
FGG	CAD	2	-0.061	0.031	-0.121	0.000	0.049	0.941	0.886	1.000	0.302
FGG	MI	2	-0.020	0.046	-0.111	0.071	0.665	0.980	0.895	1.073	0.930
FGG	HF	2	0.009	0.049	-0.086	0.105	0.851	1.009	0.917	1.110	0.971
FGG	AF	2	-0.023	0.042	-0.106	0.059	0.576	0.977	0.900	1.060	0.896
FGG	Stroke	2	0.014	0.043	-0.071	0.099	0.740	1.014	0.932	1.104	0.953
HGF	COPD	3	0.004	0.016	-0.027	0.034	0.812	1.004	0.974	1.035	0.971
HGF	Asthma	3	-0.050	0.045	-0.139	0.038	0.264	0.951	0.870	1.039	0.684

Exposure	Outcome	n	Estimate	StdError	CI95Lower	CI95Upper	p-value	OR	ORlow	ORhi	q-value
HGF	CAD	3	-0.011	0.038	-0.087	0.064	0.771	0.989	0.917	1.066	0.965
HGF	MI	3	0.012	0.058	-0.102	0.127	0.830	1.013	0.903	1.135	0.971
HGF	HF	3	0.089	0.059	-0.026	0.205	0.130	1.093	0.974	1.227	0.518
HGF	AF	3	-0.061	0.050	-0.160	0.038	0.225	0.941	0.852	1.038	0.637
HGF	Stroke	3	0.057	0.054	-0.049	0.163	0.291	1.059	0.952	1.177	0.718
HP	COPD	13	-0.006	0.002	-0.010	-0.002	0.003	0.994	0.990	0.998	0.048
HP	Asthma	13	-0.017	0.006	-0.028	-0.005	0.005	0.984	0.972	0.995	0.068
HP	CAD	12	-0.020	0.008	-0.035	-0.004	0.016	0.981	0.965	0.996	0.159
HP	MI	12	-0.019	0.013	-0.043	0.006	0.134	0.981	0.957	1.006	0.521
HP	HF	12	0.004	0.007	-0.009	0.017	0.517	1.004	0.991	1.017	0.872
HP	AF	12	0.002	0.006	-0.009	0.013	0.713	1.002	0.991	1.013	0.943
HP	Stroke	12	-0.013	0.006	-0.025	-0.001	0.031	0.987	0.975	0.999	0.231
ICAM1	COPD	6	-0.014	0.010	-0.033	0.006	0.165	0.987	0.968	1.006	0.576
ICAM1	Asthma	6	0.020	0.029	-0.036	0.076	0.480	1.020	0.965	1.079	0.848
ICAM1	CAD	6	0.009	0.015	-0.021	0.039	0.546	1.009	0.979	1.040	0.891
ICAM1	MI	6	-0.018	0.024	-0.065	0.028	0.433	0.982	0.937	1.028	0.819
ICAM1	HF	7	-0.030	0.023	-0.076	0.015	0.188	0.970	0.927	1.015	0.605
ICAM1	AF	7	-0.030	0.020	-0.069	0.009	0.136	0.971	0.934	1.009	0.527
ICAM1	Stroke	6	0.010	0.023	-0.034	0.055	0.647	1.010	0.966	1.057	0.924
IL12RB2	COPD	1	0.017	0.009	0.000	0.035	0.047	1.018	1.000	1.035	0.298
IL12RB2	Asthma	1	-0.031	0.025	-0.080	0.018	0.213	0.969	0.923	1.018	0.623
IL12RB2	CAD	1	-0.012	0.021	-0.052	0.029	0.571	0.988	0.949	1.029	0.896
IL12RB2	MI	1	-0.021	0.031	-0.081	0.040	0.507	0.980	0.922	1.041	0.870
IL12RB2	HF	1	0.007	0.032	-0.056	0.071	0.819	1.007	0.946	1.073	0.971
IL12RB2	AF	1	0.027	0.028	-0.027	0.081	0.324	1.028	0.973	1.085	0.733
IL12RB2	Stroke	1	-0.058	0.030	-0.116	0.001	0.055	0.944	0.890	1.001	0.321
IL16	COPD	9	-0.006	0.005	-0.015	0.003	0.168	0.994	0.985	1.003	0.576
IL16	Asthma	9	-0.004	0.015	-0.033	0.026	0.811	0.996	0.967	1.026	0.971
IL16	CAD	9	-0.008	0.013	-0.033	0.018	0.546	0.992	0.967	1.018	0.891
IL16	MI	9	0.003	0.022	-0.040	0.047	0.878	1.003	0.961	1.048	0.971
IL16	HF	9	-0.001	0.015	-0.030	0.028	0.960	0.999	0.971	1.028	0.999
IL16	AF	9	0.005	0.014	-0.022	0.032	0.693	1.005	0.979	1.033	0.937
IL16	Stroke	9	-0.010	0.014	-0.038	0.017	0.468	0.990	0.963	1.017	0.838
IL17D	COPD	1	-0.002	0.009	-0.020	0.016	0.795	0.998	0.980	1.016	0.971

Exposure	Outcome	n	Estimate	StdError	CI95Lower	CI95Upper	p-value	OR	ORlow	ORhi	q-value
IL17D	Asthma	1	-0.011	0.027	-0.063	0.041	0.674	0.989	0.939	1.042	0.932
IL17D	CAD	1	-0.006	0.022	-0.049	0.036	0.766	0.994	0.952	1.037	0.965
IL17D	MI	1	-0.030	0.033	-0.095	0.035	0.369	0.971	0.910	1.036	0.770
IL17D	HF	1	-0.023	0.041	-0.103	0.057	0.569	0.977	0.902	1.058	0.896
IL17D	AF	1	0.007	0.032	-0.055	0.069	0.835	1.007	0.946	1.071	0.971
IL17D	Stroke	1	-0.051	0.032	-0.115	0.013	0.117	0.950	0.892	1.013	0.490
IL17RD	COPD	5	-0.034	0.010	-0.053	-0.014	0.001	0.967	0.948	0.986	0.020
IL17RD	Asthma	5	-0.010	0.017	-0.044	0.023	0.545	0.990	0.957	1.023	0.891
IL17RD	CAD	5	-0.006	0.014	-0.033	0.021	0.668	0.994	0.968	1.021	0.932
IL17RD	MI	5	0.002	0.013	-0.024	0.028	0.876	1.002	0.976	1.028	0.971
IL17RD	HF	5	0.007	0.014	-0.021	0.035	0.626	1.007	0.979	1.035	0.912
IL17RD	AF	5	-0.035	0.019	-0.072	0.002	0.063	0.966	0.931	1.002	0.349
IL17RD	Stroke	5	0.020	0.015	-0.010	0.050	0.184	1.021	0.990	1.052	0.604
IL18	COPD	6	0.034	0.008	0.019	0.049	0.000	1.035	1.019	1.051	0.001
IL18	Asthma	6	0.010	0.024	-0.036	0.057	0.660	1.010	0.965	1.058	0.928
IL18	CAD	6	-0.004	0.019	-0.041	0.033	0.833	0.996	0.960	1.033	0.971
IL18	MI	6	0.011	0.029	-0.046	0.067	0.711	1.011	0.955	1.069	0.943
IL18	HF	6	0.039	0.029	-0.019	0.097	0.183	1.040	0.982	1.102	0.603
IL18	AF	6	0.075	0.028	0.021	0.129	0.007	1.078	1.021	1.137	0.083
IL18	Stroke	5	-0.017	0.029	-0.074	0.039	0.552	0.983	0.929	1.040	0.893
IL1R1	COPD	5	0.006	0.024	-0.040	0.053	0.786	1.006	0.961	1.054	0.971
IL1R1	Asthma	5	0.186	0.158	-0.123	0.496	0.238	1.205	0.884	1.642	0.653
IL1R1	CAD	5	0.084	0.033	0.020	0.148	0.010	1.088	1.020	1.160	0.110
IL1R1	MI	5	0.123	0.057	0.012	0.234	0.029	1.131	1.012	1.264	0.224
IL1R1	HF	5	-0.058	0.041	-0.137	0.022	0.155	0.944	0.872	1.022	0.557
IL1R1	AF	5	-0.073	0.041	-0.153	0.007	0.074	0.930	0.858	1.007	0.382
IL1R1	Stroke	5	0.072	0.036	0.001	0.142	0.048	1.074	1.001	1.153	0.300
IL1R2	COPD	14	-0.018	0.009	-0.035	-0.002	0.032	0.982	0.965	0.998	0.233
IL1R2	Asthma	14	-0.114	0.044	-0.201	-0.027	0.010	0.892	0.818	0.973	0.110
IL1R2	CAD	12	-0.020	0.010	-0.039	-0.001	0.043	0.980	0.962	0.999	0.284
IL1R2	MI	11	-0.023	0.018	-0.058	0.011	0.184	0.977	0.944	1.011	0.603
IL1R2	HF	11	0.044	0.014	0.017	0.071	0.002	1.045	1.017	1.074	0.029
IL1R2	AF	12	-0.010	0.012	-0.033	0.013	0.401	0.990	0.967	1.013	0.800
IL1R2	Stroke	11	0.016	0.014	-0.011	0.043	0.246	1.016	0.989	1.044	0.659

Exposure	Outcome	n	Estimate	StdError	CI95Lower	CI95Upper	p-value	OR	ORlow	ORhi	q-value
IL1RAP	COPD	32	-0.002	0.002	-0.005	0.001	0.134	0.998	0.995	1.001	0.521
IL1RAP	Asthma	31	-0.010	0.005	-0.021	0.000	0.050	0.990	0.980	1.000	0.307
IL1RAP	CAD	32	0.006	0.004	-0.001	0.014	0.108	1.006	0.999	1.014	0.475
IL1RAP	MI	30	0.005	0.006	-0.007	0.016	0.417	1.005	0.993	1.017	0.810
IL1RAP	HF	30	-0.013	0.006	-0.025	0.000	0.043	0.987	0.975	1.000	0.285
IL1RAP	AF	32	0.003	0.006	-0.009	0.014	0.641	1.003	0.991	1.014	0.921
IL1RAP	Stroke	29	-0.001	0.007	-0.015	0.013	0.938	0.999	0.986	1.014	0.995
IL1RL1	COPD	21	-0.023	0.005	-0.032	-0.013	0.000	0.978	0.968	0.987	0.000
IL1RL1	Asthma	21	-0.123	0.036	-0.194	-0.052	0.001	0.884	0.824	0.949	0.016
IL1RL1	CAD	21	0.013	0.007	-0.001	0.028	0.079	1.013	0.999	1.028	0.395
IL1RL1	MI	21	0.014	0.009	-0.003	0.031	0.110	1.014	0.997	1.032	0.475
IL1RL1	HF	21	0.001	0.009	-0.016	0.018	0.910	1.001	0.984	1.018	0.984
IL1RL1	AF	21	-0.008	0.009	-0.024	0.009	0.367	0.992	0.976	1.009	0.769
IL1RL1	Stroke	20	0.005	0.008	-0.010	0.021	0.507	1.005	0.990	1.021	0.870
IL1RL2	COPD	15	-0.026	0.009	-0.043	-0.008	0.004	0.975	0.957	0.992	0.062
IL1RL2	Asthma	15	-0.070	0.043	-0.154	0.014	0.104	0.932	0.857	1.014	0.462
IL1RL2	CAD	15	-0.048	0.014	-0.076	-0.020	0.001	0.953	0.927	0.980	0.017
IL1RL2	MI	15	-0.040	0.020	-0.079	-0.002	0.041	0.961	0.924	0.998	0.276
IL1RL2	HF	15	0.005	0.017	-0.030	0.039	0.794	1.005	0.971	1.039	0.971
IL1RL2	AF	16	-0.004	0.015	-0.033	0.026	0.800	0.996	0.967	1.026	0.971
IL1RL2	Stroke	15	-0.001	0.015	-0.032	0.029	0.933	0.999	0.969	1.029	0.993
IL1RN	COPD	13	-0.028	0.009	-0.046	-0.011	0.001	0.972	0.955	0.989	0.026
IL1RN	Asthma	14	-0.055	0.023	-0.100	-0.009	0.019	0.947	0.905	0.991	0.171
IL1RN	CAD	14	0.083	0.019	0.045	0.121	0.000	1.087	1.046	1.129	0.001
IL1RN	MI	14	0.194	0.031	0.133	0.255	0.000	1.214	1.142	1.291	0.000
IL1RN	HF	14	-0.002	0.031	-0.063	0.059	0.956	0.998	0.939	1.061	0.999
IL1RN	AF	14	0.013	0.025	-0.036	0.061	0.616	1.013	0.964	1.063	0.912
IL1RN	Stroke	10	-0.023	0.053	-0.126	0.080	0.662	0.977	0.881	1.084	0.928
IL23R	COPD	2	-0.005	0.007	-0.020	0.010	0.501	0.995	0.981	1.010	0.867
IL23R	Asthma	2	-0.004	0.022	-0.046	0.038	0.840	0.996	0.955	1.039	0.971
IL23R	CAD	2	-0.006	0.018	-0.042	0.029	0.725	0.994	0.959	1.030	0.947
IL23R	MI	2	-0.031	0.028	-0.085	0.023	0.260	0.969	0.918	1.023	0.677
IL23R	HF	2	0.020	0.028	-0.036	0.075	0.489	1.020	0.965	1.078	0.855
IL23R	AF	2	0.014	0.024	-0.033	0.061	0.559	1.014	0.967	1.063	0.896

Exposure	Outcome	n	Estimate	StdError	CI95Lower	CI95Upper	p-value	OR	ORlow	ORhi	q-value
IL23R	Stroke	2	-0.010	0.025	-0.060	0.039	0.681	0.990	0.942	1.040	0.932
IL27RA	COPD	7	0.002	0.003	-0.003	0.007	0.465	1.002	0.997	1.007	0.838
IL27RA	Asthma	7	0.002	0.008	-0.013	0.017	0.793	1.002	0.987	1.018	0.971
IL27RA	CAD	7	-0.004	0.007	-0.018	0.010	0.568	0.996	0.982	1.010	0.896
IL27RA	MI	7	-0.015	0.010	-0.035	0.005	0.133	0.985	0.965	1.005	0.521
IL27RA	HF	7	-0.020	0.013	-0.045	0.006	0.129	0.981	0.956	1.006	0.518
IL27RA	AF	7	-0.013	0.011	-0.034	0.007	0.207	0.987	0.967	1.007	0.615
IL27RA	Stroke	6	-0.014	0.010	-0.033	0.005	0.154	0.986	0.968	1.005	0.554
IL2RA	COPD	3	-0.015	0.011	-0.037	0.007	0.189	0.985	0.964	1.007	0.605
IL2RA	Asthma	3	-0.031	0.033	-0.095	0.033	0.343	0.970	0.910	1.033	0.752
IL2RA	CAD	3	-0.004	0.027	-0.057	0.049	0.891	0.996	0.945	1.051	0.976
IL2RA	MI	3	-0.018	0.041	-0.099	0.063	0.668	0.982	0.906	1.065	0.932
IL2RA	HF	3	-0.083	0.043	-0.167	0.002	0.054	0.921	0.846	1.002	0.321
IL2RA	AF	3	0.023	0.036	-0.047	0.094	0.517	1.024	0.954	1.099	0.872
IL2RA	Stroke	3	-0.017	0.039	-0.093	0.060	0.666	0.983	0.911	1.061	0.930
IL2RB	COPD	1	-0.032	0.021	-0.074	0.010	0.133	0.968	0.928	1.010	0.521
IL2RB	Asthma	1	-0.285	0.061	-0.405	-0.165	0.000	0.752	0.667	0.848	0.000
IL2RB	CAD	1	0.069	0.050	-0.029	0.167	0.169	1.071	0.971	1.182	0.580
IL2RB	MI	1	0.016	0.077	-0.134	0.166	0.833	1.016	0.874	1.181	0.971
IL2RB	HF	1	-0.080	0.080	-0.237	0.077	0.317	0.923	0.789	1.080	0.731
IL2RB	AF	1	0.100	0.068	-0.033	0.233	0.140	1.106	0.968	1.263	0.531
IL2RB	Stroke	1	-0.059	0.073	-0.202	0.084	0.420	0.943	0.817	1.088	0.812
IL6R	COPD	25	0.000	0.003	-0.006	0.006	0.990	1.000	0.994	1.006	1.000
IL6R	Asthma	25	0.029	0.010	0.010	0.048	0.002	1.029	1.010	1.049	0.040
IL6R	CAD	25	-0.043	0.006	-0.054	-0.032	0.000	0.958	0.947	0.968	0.000
IL6R	MI	24	-0.038	0.007	-0.052	-0.024	0.000	0.963	0.949	0.976	0.000
IL6R	HF	24	-0.011	0.010	-0.030	0.007	0.235	0.989	0.971	1.007	0.649
IL6R	AF	25	-0.044	0.008	-0.059	-0.030	0.000	0.957	0.943	0.971	0.000
IL6R	Stroke	23	-0.023	0.006	-0.035	-0.011	0.000	0.977	0.965	0.989	0.006
IL6ST	COPD	13	0.014	0.006	0.003	0.025	0.013	1.014	1.003	1.025	0.135
IL6ST	Asthma	13	0.054	0.015	0.025	0.082	0.000	1.055	1.025	1.086	0.007
IL6ST	CAD	13	0.023	0.017	-0.010	0.056	0.165	1.023	0.990	1.057	0.576
IL6ST	MI	12	0.049	0.019	0.011	0.087	0.011	1.050	1.011	1.091	0.121
IL6ST	HF	12	0.015	0.020	-0.024	0.054	0.458	1.015	0.976	1.055	0.836

Exposure	Outcome	n	Estimate	StdError	CI95Lower	CI95Upper	p-value	OR	ORlow	ORhi	q-value
IL6ST	AF	13	0.052	0.017	0.019	0.085	0.002	1.053	1.019	1.089	0.035
IL6ST	Stroke	11	-0.010	0.018	-0.045	0.025	0.574	0.990	0.956	1.025	0.896
IL7R	COPD	7	0.025	0.008	0.010	0.041	0.001	1.026	1.010	1.042	0.023
IL7R	Asthma	7	0.191	0.054	0.086	0.297	0.000	1.211	1.090	1.346	0.010
IL7R	CAD	7	0.006	0.019	-0.030	0.043	0.738	1.006	0.970	1.044	0.953
IL7R	MI	7	0.024	0.028	-0.032	0.079	0.403	1.024	0.969	1.082	0.800
IL7R	HF	7	-0.030	0.030	-0.088	0.028	0.315	0.971	0.916	1.029	0.731
IL7R	AF	7	-0.057	0.031	-0.117	0.003	0.064	0.945	0.890	1.003	0.352
IL7R	Stroke	7	-0.001	0.029	-0.058	0.057	0.979	0.999	0.943	1.059	1.000
MBL2	COPD	18	0.000	0.002	-0.003	0.004	0.762	1.000	0.997	1.004	0.965
MBL2	Asthma	18	-0.003	0.004	-0.012	0.005	0.426	0.997	0.988	1.005	0.815
MBL2	CAD	18	-0.004	0.004	-0.011	0.003	0.300	0.996	0.989	1.003	0.723
MBL2	MI	17	0.000	0.006	-0.011	0.011	0.991	1.000	0.989	1.011	1.000
MBL2	HF	18	-0.009	0.009	-0.026	0.008	0.296	0.991	0.974	1.008	0.718
MBL2	AF	19	-0.011	0.005	-0.021	-0.002	0.023	0.989	0.979	0.998	0.195
MBL2	Stroke	16	-0.004	0.007	-0.019	0.010	0.556	0.996	0.982	1.010	0.896
MIF	COPD	1	-0.030	0.014	-0.057	-0.003	0.029	0.970	0.944	0.997	0.224
MIF	Asthma	1	-0.050	0.040	-0.127	0.028	0.206	0.951	0.880	1.028	0.615
MIF	CAD	1	0.140	0.032	0.077	0.204	0.000	1.151	1.080	1.226	0.001
MIF	MI	1	0.090	0.049	-0.006	0.186	0.067	1.094	0.994	1.204	0.359
MIF	HF	1	0.124	0.052	0.023	0.225	0.016	1.132	1.023	1.253	0.159
MIF	AF	1	-0.020	0.044	-0.106	0.066	0.648	0.980	0.899	1.068	0.924
MIF	Stroke	1	-0.003	0.047	-0.096	0.089	0.945	0.997	0.909	1.093	0.996
PGF	COPD	2	-0.016	0.016	-0.046	0.015	0.323	0.985	0.955	1.015	0.733
PGF	Asthma	2	0.043	0.045	-0.046	0.132	0.346	1.044	0.955	1.141	0.755
PGF	CAD	2	-0.284	0.038	-0.357	-0.210	0.000	0.753	0.699	0.810	0.000
PGF	MI	2	-0.272	0.057	-0.385	-0.159	0.000	0.762	0.681	0.853	0.000
PGF	HF	2	0.019	0.059	-0.097	0.135	0.746	1.019	0.908	1.145	0.956
PGF	AF	2	0.028	0.050	-0.071	0.127	0.580	1.028	0.932	1.135	0.896
PGF	Stroke	2	-0.055	0.054	-0.162	0.052	0.312	0.946	0.851	1.053	0.729
SAA1	COPD	12	0.008	0.004	0.000	0.015	0.051	1.008	1.000	1.015	0.310
SAA1	Asthma	12	0.009	0.015	-0.020	0.037	0.555	1.009	0.980	1.038	0.896
SAA1	CAD	12	0.020	0.009	0.002	0.037	0.030	1.020	1.002	1.038	0.225
SAA1	MI	12	0.032	0.019	-0.006	0.070	0.097	1.032	0.994	1.072	0.448

Exposure	Outcome	n	Estimate	StdError	CI95Lower	CI95Upper	p-value	OR	ORlow	ORhi	q-value
SAA1	HF	12	0.018	0.014	-0.010	0.046	0.205	1.018	0.990	1.047	0.615
SAA1	AF	12	0.004	0.012	-0.020	0.027	0.752	1.004	0.980	1.028	0.958
SAA1	Stroke	11	0.014	0.013	-0.011	0.039	0.257	1.015	0.990	1.040	0.675
SAA2	COPD	12	0.007	0.004	0.000	0.015	0.059	1.007	1.000	1.015	0.333
SAA2	Asthma	12	0.010	0.016	-0.022	0.041	0.549	1.010	0.978	1.042	0.892
SAA2	CAD	12	0.028	0.009	0.009	0.046	0.003	1.028	1.009	1.047	0.053
SAA2	MI	10	0.044	0.019	0.007	0.080	0.019	1.045	1.007	1.083	0.171
SAA2	HF	10	0.015	0.016	-0.016	0.045	0.350	1.015	0.984	1.047	0.755
SAA2	AF	10	0.000	0.015	-0.030	0.029	0.984	1.000	0.971	1.030	1.000
SAA2	Stroke	8	0.004	0.014	-0.024	0.032	0.791	1.004	0.976	1.033	0.971
SERPINE1	COPD	4	-0.010	0.015	-0.040	0.019	0.495	0.990	0.961	1.019	0.860
SERPINE1	Asthma	4	-0.061	0.043	-0.146	0.024	0.158	0.941	0.864	1.024	0.562
SERPINE1	CAD	4	0.038	0.037	-0.035	0.110	0.305	1.039	0.966	1.117	0.727
SERPINE1	MI	4	0.017	0.056	-0.093	0.126	0.768	1.017	0.911	1.135	0.965
SERPINE1	HF	4	-0.067	0.063	-0.191	0.058	0.293	0.936	0.826	1.059	0.718
SERPINE1	AF	4	0.132	0.059	0.016	0.248	0.026	1.141	1.016	1.281	0.208
SERPINE1	Stroke	4	0.140	0.057	0.029	0.251	0.013	1.151	1.030	1.286	0.136
TNFSF10	COPD	4	0.010	0.017	-0.023	0.043	0.562	1.010	0.977	1.044	0.896
TNFSF10	Asthma	4	0.055	0.034	-0.011	0.121	0.100	1.057	0.990	1.129	0.451
TNFSF10	CAD	4	0.037	0.027	-0.015	0.090	0.165	1.038	0.985	1.095	0.576
TNFSF10	MI	4	0.060	0.042	-0.022	0.143	0.152	1.062	0.978	1.154	0.550
TNFSF10	HF	4	-0.015	0.047	-0.108	0.078	0.749	0.985	0.897	1.081	0.956
TNFSF10	AF	4	0.009	0.058	-0.104	0.122	0.874	1.009	0.901	1.130	0.971
TNFSF10	Stroke	4	0.010	0.055	-0.098	0.118	0.851	1.010	0.907	1.126	0.971
VEGFA	COPD	18	0.011	0.005	0.001	0.021	0.024	1.011	1.001	1.021	0.198
VEGFA	Asthma	18	-0.009	0.013	-0.034	0.017	0.504	0.991	0.967	1.017	0.869
VEGFA	CAD	18	-0.003	0.011	-0.025	0.020	0.821	0.997	0.975	1.020	0.971
VEGFA	MI	17	-0.011	0.015	-0.041	0.018	0.444	0.989	0.960	1.018	0.824
VEGFA	HF	18	0.029	0.017	-0.004	0.063	0.087	1.030	0.996	1.065	0.418
VEGFA	AF	18	0.009	0.015	-0.021	0.039	0.556	1.009	0.979	1.040	0.896
VEGFA	Stroke	18	0.016	0.016	-0.015	0.048	0.315	1.016	0.985	1.049	0.731
VEGFC	COPD	1	-0.025	0.016	-0.055	0.006	0.110	0.975	0.946	1.006	0.475
VEGFC	Asthma	1	0.007	0.044	-0.080	0.094	0.878	1.007	0.923	1.099	0.971
VEGFC	CAD	1	0.011	0.039	-0.065	0.087	0.773	1.011	0.937	1.091	0.965

Exposure	Outcome	n	Estimate	StdError	CI95Lower	CI95Upper	p-value	OR	ORlow	ORhi	q-value
VEGFC	MI	1	-0.016	0.061	-0.135	0.103	0.794	0.984	0.874	1.109	0.971
VEGFC	HF	1	-0.042	0.063	-0.166	0.082	0.509	0.959	0.847	1.086	0.870
VEGFC	AF	1	0.010	0.053	-0.093	0.114	0.845	1.010	0.911	1.121	0.971
VEGFC	Stroke	1	0.081	0.058	-0.034	0.195	0.167	1.084	0.967	1.215	0.576

Blue highlight denotes suggestive evidence based on FDR-corrected p-value (q-value).

Orange highlight denotes strong evidence based on FDR-corrected p-value (q-value).

CI95Lower: 95% lower CI for the estimate; Ci95Upper: 95% upper CI for the estimate.

ORlow: 95% lower CI for the OR; ORhi: 95% upper CI for the OR.

AF: atrial fibrillation, CAD: coronary artery disease, COPD: chronic obstructive pulmonary diseases, HF: heart failure, IS: ischemia stroke, MI: myocardial infarction, n: number of SNPs used as instrument variables in each method, SNPs single nucleotide polymorphism, CI confidence intervals, OR odds ratio.

Supplement Table 8. Cis-MR IVW and sensitivity analyses results for inflammatory markers that associated with both chronic respiratory diseases and cardiovascular diseases.

Exposure	Outcome	n	Method	Estimate	StdError	CI95Lower	CI95Upper	p-value	OR	ORlow	ORhi	q-value
IL6R	COPD	25	IVW	0.000	0.003	-0.006	0.006	0.990	1.000	0.994	1.006	1.000
		25	Weighted median	0.003	0.003	-0.002	0.008	0.265	1.003	0.998	1.008	0.685
		25	MR-Egger	-0.001	0.006	-0.013	0.011	0.866	0.999	0.987	1.011	0.971
		22	MR-PRESSO	0.003	0.002	-0.001	0.008	0.174	1.003	0.999	1.008	0.593
		25	Contamination mixture	0.003	0.000	0.003	0.003	0.085	1.003	1.003	1.003	0.410
	Asthma	25	IVW	0.029	0.010	0.010	0.048	0.002	1.029	1.010	1.049	0.040
		25	Weighted median	0.039	0.009	0.023	0.056	0.000	1.040	1.023	1.058	0.000
		25	MR-Egger	0.017	0.019	-0.020	0.055	0.364	1.017	0.980	1.056	0.767
		23	MR-PRESSO	0.032	0.008	0.016	0.049	0.001	1.033	1.016	1.050	0.020
		25	Contamination mixture	0.054	0.003	-0.006	0.004	0.098	1.055	0.994	1.004	0.450
		25	Contamination mixture	0.054	0.010	0.024	0.064	0.098	1.055	1.024	1.066	0.450
	CAD	25	IVW	-0.043	0.006	-0.054	-0.032	0.000	0.958	0.947	0.968	0.000
		25	Weighted median	-0.045	0.006	-0.057	-0.034	0.000	0.956	0.945	0.967	0.000
		25	MR-Egger	-0.052	0.011	-0.074	-0.030	0.000	0.950	0.929	0.971	0.000
		25	MR-PRESSO	-0.043	0.006	-0.054	-0.032	0.000	0.958	0.947	0.968	0.000
		25	Contamination mixture	-0.050	0.005	-0.060	-0.040	0.000	0.951	0.942	0.961	0.000
	MI	24	IVW	-0.038	0.007	-0.052	-0.024	0.000	0.963	0.949	0.976	0.000
		24	Weighted median	-0.046	0.009	-0.063	-0.030	0.000	0.955	0.939	0.971	0.000
		24	MR-Egger	-0.047	0.014	-0.076	-0.019	0.001	0.954	0.927	0.981	0.022
		24	MR-PRESSO	-0.038	0.007	-0.052	-0.024	0.000	0.963	0.949	0.976	0.001
		24	Contamination mixture	-0.046	0.005	-0.056	-0.036	0.000	0.955	0.946	0.965	0.000
	HF	24	IVW	-0.011	0.010	-0.030	0.007	0.235	0.989	0.971	1.007	0.649
		24	Weighted median	-0.014	0.009	-0.032	0.005	0.149	0.987	0.969	1.005	0.548
		24	MR-Egger	-0.029	0.019	-0.067	0.009	0.136	0.971	0.935	1.009	0.527
		23	MR-PRESSO	-0.010	0.009	-0.027	0.007	0.245	0.990	0.973	1.007	0.658
		24	Contamination mixture	-0.016	0.005	-0.026	-0.006	0.025	0.984	0.974	0.994	0.203
	AF	25	IVW	-0.044	0.008	-0.059	-0.030	0.000	0.957	0.943	0.971	0.000
		25	Weighted median	-0.047	0.008	-0.062	-0.032	0.000	0.954	0.940	0.969	0.000
		25	MR-Egger	-0.068	0.015	-0.097	-0.039	0.000	0.934	0.908	0.962	0.000
		24	MR-PRESSO	-0.041	0.007	-0.055	-0.028	0.000	0.960	0.947	0.972	0.000
		25	Contamination mixture	-0.052	0.005	-0.062	-0.042	0.000	0.949	0.940	0.959	0.010
	Stroke	23	IVW	-0.023	0.006	-0.035	-0.011	0.000	0.977	0.965	0.989	0.006
		23	Weighted median	-0.021	0.009	-0.038	-0.003	0.020	0.980	0.963	0.997	0.175
		23	MR-Egger	-0.016	0.013	-0.043	0.010	0.225	0.984	0.958	1.010	0.638

Exposure	Outcome	n	Method	Estimate	StdError	CI95Lower	CI95Upper	p-value	OR	ORlow	ORhi	q-value
		23	MR-PRESSO	-0.023	0.006	-0.035	-0.011	0.001	0.977	0.965	0.989	0.023
		23	Contamination mixture	-0.017	0.005	-0.027	-0.007	0.045	0.983	0.973	0.993	0.287
IL1RN	COPD	13	IVW	-0.028	0.009	-0.046	-0.011	0.001	0.972	0.955	0.989	0.026
		13	Weighted median	-0.032	0.009	-0.050	-0.015	0.000	0.968	0.951	0.985	0.009
		13	MR-Egger	-0.053	0.019	-0.090	-0.016	0.005	0.948	0.914	0.984	0.072
		13	MR-PRESSO	-0.028	0.009	-0.046	-0.011	0.008	0.972	0.955	0.989	0.090
		13	Contamination mixture	-0.028	0.008	-0.048	-0.018	0.027	0.972	0.953	0.982	0.210
	Asthma	14	IVW	-0.055	0.023	-0.100	-0.009	0.019	0.947	0.905	0.991	0.171
		14	Weighted median	-0.061	0.025	-0.111	-0.011	0.017	0.941	0.895	0.989	0.163
		14	MR-Egger	-0.044	0.055	-0.152	0.064	0.421	0.957	0.859	1.066	0.812
		14	MR-PRESSO	-0.055	0.023	-0.100	-0.009	0.035	0.947	0.905	0.991	0.247
		14	Contamination mixture	-0.097	0.038	-0.177	-0.027	0.009	0.907	0.838	0.973	0.105
	CAD	14	IVW	0.083	0.019	0.045	0.121	0.000	1.087	1.046	1.129	0.001
		14	Weighted median	0.082	0.023	0.037	0.127	0.000	1.085	1.038	1.135	0.010
		14	MR-Egger	0.074	0.045	-0.014	0.162	0.100	1.077	0.986	1.176	0.451
		14	MR-PRESSO	0.083	0.019	0.045	0.121	0.001	1.087	1.046	1.129	0.020
		14	Contamination mixture	0.111	0.036	0.061	0.201	0.001	1.117	1.063	1.222	0.016
	MI	14	IVW	0.194	0.031	0.133	0.255	0.000	1.214	1.142	1.291	0.000
		14	Weighted median	0.190	0.033	0.125	0.255	0.000	1.209	1.133	1.291	0.000
		14	MR-Egger	0.177	0.073	0.034	0.320	0.015	1.194	1.035	1.377	0.152
		14	MR-PRESSO	0.194	0.031	0.133	0.255	0.000	1.214	1.142	1.291	0.001
		14	Contamination mixture	0.188	0.033	0.148	0.278	0.000	1.207	1.160	1.321	0.001
	HF	14	IVW	-0.002	0.031	-0.063	0.059	0.956	0.998	0.939	1.061	0.999
		14	Weighted median	0.043	0.043	-0.041	0.127	0.310	1.044	0.960	1.136	0.727
		14	MR-Egger	0.062	0.067	-0.070	0.194	0.356	1.064	0.933	1.214	0.765
		14	MR-PRESSO	-0.002	0.031	-0.063	0.059	0.957	0.998	0.939	1.061	0.999
		14	Contamination mixture	0.060	0.041	-0.040	0.120	0.360	1.062	0.961	1.127	0.767
	AF	14	IVW	0.013	0.025	-0.036	0.061	0.616	1.013	0.964	1.063	0.912
		14	Weighted median	-0.005	0.033	-0.069	0.060	0.881	0.995	0.933	1.061	0.973
		14	MR-Egger	-0.007	0.054	-0.113	0.099	0.893	0.993	0.893	1.104	0.976
		14	MR-PRESSO	0.013	0.020	-0.027	0.052	0.547	1.013	0.973	1.054	0.891
		14	Contamination mixture	0.015	0.036	-0.045	0.095	0.591	1.015	0.956	1.099	0.899
	Stroke	10	IVW	-0.023	0.053	-0.126	0.080	0.662	0.977	0.881	1.084	0.928
		10	Weighted median	-0.029	0.058	-0.142	0.084	0.619	0.972	0.868	1.088	0.912
		10	MR-Egger	0.032	0.125	-0.213	0.278	0.797	1.033	0.808	1.321	0.971
		10	MR-PRESSO	-0.023	0.053	-0.126	0.080	0.673	0.977	0.881	1.084	0.932
		10	Contamination mixture	0.045	0.153	-0.355	0.245	0.348	1.046	0.701	1.277	0.755
CXCL6	COPD	12	IVW	-0.014	0.004	-0.021	-0.007	0.000	0.986	0.979	0.994	0.006

Exposure	Outcome	n	Method	Estimate	StdError	CI95Lower	CI95Upper	p-value	OR	ORlow	ORhi	q-value
		12	Weighted median	-0.010	0.004	-0.018	-0.001	0.033	0.990	0.982	0.999	0.233
		12	MR-Egger	-0.002	0.009	-0.020	0.016	0.841	0.998	0.980	1.016	0.971
		12	MR-PRESSO	-0.014	0.004	-0.021	-0.007	0.003	0.986	0.979	0.994	0.051
		12	Contamination mixture	-0.025	0.008	-0.035	-0.005	0.019	0.975	0.965	0.995	0.171
	Asthma	12	IVW	-0.003	0.014	-0.030	0.025	0.845	0.997	0.970	1.025	0.971
		12	Weighted median	0.011	0.013	-0.015	0.036	0.415	1.011	0.985	1.037	0.808
		12	MR-Egger	0.055	0.033	-0.011	0.120	0.101	1.056	0.989	1.128	0.455
		12	MR-PRESSO	-0.003	0.014	-0.030	0.025	0.849	0.997	0.970	1.025	0.971
		12	Contamination mixture	-0.073	0.020	-0.113	-0.033	0.056	0.930	0.893	0.968	0.321
		12	Contamination mixture	-0.073	0.013	0.007	0.057	0.056	0.930	1.007	1.059	0.321
	CAD	12	IVW	0.012	0.010	-0.008	0.032	0.238	1.012	0.992	1.032	0.653
		12	Weighted median	0.019	0.011	-0.003	0.041	0.095	1.019	0.997	1.041	0.445
		12	MR-Egger	0.004	0.027	-0.050	0.057	0.895	1.004	0.951	1.059	0.978
		12	MR-PRESSO	0.012	0.010	-0.008	0.032	0.263	1.012	0.992	1.032	0.684
		12	Contamination mixture	0.028	0.015	0.008	0.068	0.020	1.028	1.008	1.070	0.173
	MI	12	IVW	0.031	0.013	0.006	0.055	0.014	1.031	1.006	1.057	0.140
		12	Weighted median	0.041	0.017	0.009	0.074	0.013	1.042	1.009	1.077	0.134
		12	MR-Egger	0.036	0.033	-0.028	0.099	0.273	1.036	0.972	1.105	0.695
		12	MR-PRESSO	0.031	0.011	0.008	0.053	0.021	1.031	1.008	1.055	0.179
		12	Contamination mixture	0.046	0.013	0.026	0.076	0.017	1.047	1.026	1.079	0.163
	HF	12	IVW	0.034	0.013	0.008	0.059	0.010	1.034	1.008	1.061	0.110
		12	Weighted median	0.032	0.016	-0.001	0.064	0.055	1.032	0.999	1.066	0.321
		12	MR-Egger	0.021	0.034	-0.046	0.087	0.544	1.021	0.955	1.091	0.891
		12	MR-PRESSO	0.034	0.009	0.017	0.051	0.003	1.034	1.017	1.052	0.041
		12	Contamination mixture	0.040	0.015	0.010	0.070	0.010	1.041	1.010	1.073	0.110
	AF	12	IVW	0.041	0.013	0.016	0.065	0.001	1.042	1.016	1.068	0.022
		12	Weighted median	0.039	0.014	0.011	0.067	0.006	1.040	1.011	1.069	0.077
		12	MR-Egger	0.022	0.033	-0.044	0.088	0.510	1.022	0.957	1.091	0.870
		12	MR-PRESSO	0.041	0.013	0.016	0.065	0.008	1.042	1.016	1.068	0.090
		12	Contamination mixture	0.044	0.010	0.024	0.064	0.005	1.045	1.024	1.066	0.068
	Stroke	12	IVW	0.005	0.012	-0.018	0.028	0.681	1.005	0.982	1.029	0.932
		12	Weighted median	0.010	0.015	-0.019	0.040	0.482	1.011	0.981	1.040	0.850
		12	MR-Egger	0.022	0.031	-0.038	0.083	0.469	1.023	0.962	1.087	0.838
		12	MR-PRESSO	0.005	0.008	-0.012	0.022	0.576	1.005	0.988	1.022	0.896
		12	Contamination mixture	0.013	0.020	-0.047	0.033	0.361	1.013	0.954	1.033	0.767
IL1RL2	COPD	15	IVW	-0.026	0.009	-0.043	-0.008	0.004	0.975	0.957	0.992	0.062
		15	Weighted median	-0.033	0.008	-0.049	-0.018	0.000	0.967	0.953	0.982	0.001
		15	MR-Egger	-0.003	0.019	-0.041	0.035	0.876	0.997	0.960	1.035	0.971

Exposure	Outcome	n	Method	Estimate	StdError	CI95Lower	CI95Upper	p-value	OR	ORlow	ORhi	q-value
		12	MR-PRESSO	-0.036	0.006	-0.049	-0.024	0.000	0.964	0.953	0.976	0.004
		15	Contamination mixture	-0.044	0.005	-0.054	-0.034	0.003	0.957	0.948	0.967	0.041
	Asthma	15	IVW	-0.070	0.043	-0.154	0.014	0.104	0.932	0.857	1.014	0.462
		15	Weighted median	-0.063	0.031	-0.123	-0.002	0.043	0.939	0.884	0.998	0.284
		15	MR-Egger	-0.113	0.098	-0.305	0.079	0.248	0.893	0.737	1.082	0.662
		9	MR-PRESSO	-0.072	0.035	-0.140	-0.003	0.076	0.931	0.869	0.997	0.386
		15	Contamination mixture	-0.021	0.015	-0.241	-0.181	0.295	0.979	0.786	0.835	0.718
		15	Contamination mixture	-0.021	0.071	-0.141	0.139	0.295	0.979	0.869	1.149	0.718
	CAD	15	IVW	-0.048	0.014	-0.076	-0.020	0.001	0.953	0.927	0.980	0.017
		15	Weighted median	-0.053	0.015	-0.083	-0.023	0.000	0.948	0.920	0.977	0.012
		15	MR-Egger	-0.069	0.032	-0.133	-0.006	0.032	0.933	0.876	0.994	0.233
		15	MR-PRESSO	-0.048	0.014	-0.076	-0.020	0.005	0.953	0.927	0.980	0.066
		15	Contamination mixture	-0.046	0.010	-0.066	-0.026	0.005	0.955	0.936	0.974	0.068
	MI	15	IVW	-0.040	0.020	-0.079	-0.002	0.041	0.961	0.924	0.998	0.276
		15	Weighted median	-0.046	0.023	-0.091	-0.001	0.047	0.955	0.913	0.999	0.298
		15	MR-Egger	-0.066	0.045	-0.155	0.022	0.142	0.936	0.856	1.022	0.535
		15	MR-PRESSO	-0.040	0.020	-0.079	-0.002	0.060	0.961	0.924	0.998	0.337
		15	Contamination mixture	-0.062	0.015	-0.092	-0.032	0.005	0.940	0.912	0.968	0.068
	HF	15	IVW	0.005	0.017	-0.030	0.039	0.794	1.005	0.971	1.039	0.971
		15	Weighted median	0.006	0.023	-0.040	0.052	0.802	1.006	0.961	1.053	0.971
		15	MR-Egger	-0.041	0.038	-0.116	0.033	0.280	0.960	0.891	1.034	0.703
		15	MR-PRESSO	0.005	0.015	-0.024	0.033	0.762	1.005	0.976	1.034	0.965
		15	Contamination mixture	0.001	0.026	-0.049	0.051	0.860	1.001	0.953	1.053	0.971
	AF	16	IVW	-0.004	0.015	-0.033	0.026	0.800	0.996	0.967	1.026	0.971
		16	Weighted median	-0.011	0.022	-0.054	0.033	0.625	0.989	0.947	1.033	0.912
		16	MR-Egger	-0.007	0.034	-0.073	0.059	0.832	0.993	0.929	1.061	0.971
		16	MR-PRESSO	-0.004	0.014	-0.032	0.024	0.796	0.996	0.968	1.025	0.971
		16	Contamination mixture	-0.038	0.031	-0.098	0.022	0.212	0.963	0.907	1.022	0.623
	Stroke	15	IVW	-0.001	0.015	-0.032	0.029	0.933	0.999	0.969	1.029	0.993
		15	Weighted median	-0.002	0.021	-0.042	0.039	0.941	0.998	0.959	1.040	0.995
		15	MR-Egger	-0.020	0.035	-0.087	0.048	0.567	0.980	0.916	1.049	0.896
		15	MR-PRESSO	-0.001	0.015	-0.031	0.029	0.934	0.999	0.969	1.029	0.993
		15	Contamination mixture	0.005	0.018	-0.035	0.035	1.000	1.005	0.966	1.036	1.000
HP	COPD	13	IVW	-0.006	0.002	-0.010	-0.002	0.003	0.994	0.990	0.998	0.048
		13	Weighted median	-0.004	0.003	-0.009	0.001	0.112	0.996	0.991	1.001	0.482
		13	MR-Egger	-0.004	0.006	-0.016	0.008	0.522	0.996	0.985	1.008	0.877
		13	MR-PRESSO	-0.006	0.002	-0.010	-0.002	0.012	0.994	0.990	0.998	0.126
		13	Contamination mixture	-0.010	0.003	-0.010	0.000	0.572	0.990	0.990	1.000	0.896

Exposure	Outcome	n	Method	Estimate	StdError	CI95Lower	CI95Upper	p-value	OR	ORlow	ORhi	q-value
	Asthma	13	IVW	-0.017	0.006	-0.028	-0.005	0.005	0.984	0.972	0.995	0.068
		13	Weighted median	-0.021	0.007	-0.034	-0.008	0.001	0.979	0.966	0.992	0.023
		13	MR-Egger	-0.043	0.014	-0.071	-0.014	0.003	0.958	0.931	0.986	0.049
		13	MR-PRESSO	-0.017	0.006	-0.028	-0.005	0.016	0.984	0.972	0.995	0.155
		13	Contamination mixture	-0.025	0.003	-0.025	-0.015	0.006	0.976	0.976	0.986	0.080
	CAD	12	IVW	-0.020	0.008	-0.035	-0.004	0.016	0.981	0.965	0.996	0.159
		12	Weighted median	-0.022	0.006	-0.034	-0.009	0.001	0.979	0.966	0.991	0.016
		12	MR-Egger	-0.037	0.023	-0.082	0.008	0.109	0.964	0.922	1.008	0.475
		9	MR-PRESSO	-0.020	0.007	-0.033	-0.006	0.021	0.980	0.967	0.994	0.176
		12	Contamination mixture	-0.019	0.008	-0.029	0.001	0.069	0.982	0.972	1.001	0.365
	MI	12	IVW	-0.019	0.013	-0.043	0.006	0.134	0.981	0.957	1.006	0.521
		12	Weighted median	-0.013	0.009	-0.031	0.004	0.140	0.987	0.970	1.004	0.532
		12	MR-Egger	-0.045	0.035	-0.115	0.024	0.201	0.956	0.892	1.024	0.615
		8	MR-PRESSO	-0.012	0.006	-0.024	0.000	0.088	0.988	0.977	1.000	0.422
		12	Contamination mixture	-0.007	0.005	-0.017	0.003	0.192	0.993	0.983	1.003	0.605
	HF	12	IVW	0.004	0.007	-0.009	0.017	0.517	1.004	0.991	1.017	0.872
		12	Weighted median	-0.003	0.009	-0.020	0.014	0.701	0.997	0.980	1.014	0.942
		12	MR-Egger	-0.005	0.019	-0.042	0.032	0.794	0.995	0.959	1.032	0.971
		12	MR-PRESSO	0.004	0.007	-0.009	0.017	0.531	1.004	0.991	1.017	0.883
		12	Contamination mixture	0.001	0.008	-0.019	0.011	0.828	1.001	0.981	1.011	0.971
	AF	12	IVW	0.002	0.006	-0.009	0.013	0.713	1.002	0.991	1.013	0.943
		12	Weighted median	0.005	0.007	-0.009	0.020	0.456	1.005	0.991	1.020	0.835
		12	MR-Egger	0.006	0.017	-0.027	0.038	0.734	1.006	0.973	1.039	0.951
		12	MR-PRESSO	0.002	0.006	-0.009	0.013	0.720	1.002	0.991	1.013	0.945
		12	Contamination mixture	0.002	0.005	-0.008	0.012	0.581	1.002	0.992	1.012	0.896
	Stroke	12	IVW	-0.013	0.006	-0.025	-0.001	0.031	0.987	0.975	0.999	0.231
		12	Weighted median	-0.017	0.008	-0.033	-0.001	0.033	0.983	0.967	0.999	0.234
		12	MR-Egger	-0.021	0.017	-0.055	0.013	0.219	0.979	0.946	1.013	0.628
		12	MR-PRESSO	-0.013	0.006	-0.025	-0.001	0.054	0.987	0.975	0.999	0.321
		12	Contamination mixture	-0.030	0.013	-0.040	0.010	0.205	0.971	0.961	1.010	0.615
IL1R2	COPD	14	IVW	-0.018	0.009	-0.035	-0.002	0.032	0.982	0.965	0.998	0.233
		14	Weighted median	-0.014	0.005	-0.023	-0.005	0.002	0.986	0.977	0.995	0.040
		14	MR-Egger	0.003	0.017	-0.031	0.036	0.871	1.003	0.970	1.037	0.971
		11	MR-PRESSO	-0.020	0.006	-0.031	-0.009	0.005	0.980	0.970	0.991	0.067
		14	Contamination mixture	-0.020	0.010	-0.050	-0.010	0.000	0.980	0.951	0.990	0.011
	Asthma	14	IVW	-0.114	0.044	-0.201	-0.027	0.010	0.892	0.818	0.973	0.110
		14	Weighted median	-0.061	0.014	-0.089	-0.034	0.000	0.941	0.915	0.966	0.001
		14	MR-Egger	-0.017	0.089	-0.190	0.157	0.850	0.983	0.827	1.170	0.971

Exposure	Outcome	n	Method	Estimate	StdError	CI95Lower	CI95Upper	p-value	OR	ORlow	ORhi	q-value
		7	MR-PRESSO	-0.055	0.025	-0.104	-0.006	0.070	0.946	0.901	0.994	0.370
		14	Contamination mixture	-0.047	0.010	-0.077	-0.037	0.004	0.954	0.926	0.964	0.056
	CAD	12	IVW	-0.020	0.010	-0.039	-0.001	0.043	0.980	0.962	0.999	0.284
		12	Weighted median	-0.018	0.011	-0.039	0.004	0.104	0.982	0.962	1.004	0.462
		12	MR-Egger	-0.020	0.021	-0.060	0.021	0.343	0.980	0.941	1.021	0.752
		12	MR-PRESSO	-0.020	0.010	-0.039	-0.001	0.067	0.980	0.962	0.999	0.359
		12	Contamination mixture	-0.029	0.008	-0.039	-0.009	0.024	0.971	0.962	0.991	0.196
	MI	11	IVW	-0.023	0.018	-0.058	0.011	0.184	0.977	0.944	1.011	0.603
		11	Weighted median	-0.017	0.017	-0.051	0.016	0.315	0.983	0.951	1.016	0.731
		11	MR-Egger	0.040	0.030	-0.020	0.099	0.190	1.041	0.980	1.104	0.605
		11	MR-PRESSO	-0.023	0.018	-0.058	0.011	0.213	0.977	0.944	1.011	0.623
		11	Contamination mixture	-0.085	0.020	-0.125	-0.045	0.024	0.918	0.882	0.956	0.198
	HF	11	IVW	0.044	0.014	0.017	0.071	0.002	1.045	1.017	1.074	0.029
		11	Weighted median	0.034	0.017	0.001	0.067	0.046	1.034	1.001	1.069	0.293
		11	MR-Egger	0.037	0.029	-0.020	0.094	0.202	1.038	0.980	1.099	0.615
		11	MR-PRESSO	0.044	0.011	0.023	0.065	0.002	1.045	1.023	1.067	0.040
		11	Contamination mixture	0.047	0.028	0.017	0.127	0.011	1.049	1.018	1.136	0.118
	AF	12	IVW	-0.010	0.012	-0.033	0.013	0.401	0.990	0.967	1.013	0.800
		12	Weighted median	-0.007	0.014	-0.036	0.021	0.619	0.993	0.965	1.021	0.912
		12	MR-Egger	0.010	0.025	-0.039	0.059	0.686	1.010	0.962	1.061	0.933
		12	MR-PRESSO	-0.010	0.010	-0.030	0.010	0.341	0.990	0.971	1.010	0.752
		12	Contamination mixture	-0.004	0.010	-0.024	0.016	0.695	0.996	0.977	1.017	0.938
	Stroke	11	IVW	0.016	0.014	-0.011	0.043	0.246	1.016	0.989	1.044	0.659
		11	Weighted median	0.014	0.015	-0.015	0.043	0.351	1.014	0.985	1.044	0.756
		11	MR-Egger	-0.010	0.029	-0.067	0.047	0.734	0.990	0.935	1.048	0.951
		11	MR-PRESSO	0.016	0.014	-0.011	0.043	0.273	1.016	0.989	1.044	0.695
		11	Contamination mixture	0.009	0.013	-0.021	0.029	0.568	1.010	0.980	1.030	0.896
IL6ST	COPD	13	IVW	0.014	0.006	0.003	0.025	0.013	1.014	1.003	1.025	0.135
		13	Weighted median	0.013	0.007	0.000	0.026	0.059	1.013	1.000	1.026	0.334
		13	MR-Egger	0.015	0.011	-0.007	0.037	0.181	1.015	0.993	1.038	0.599
		13	MR-PRESSO	0.014	0.006	0.003	0.025	0.029	1.014	1.003	1.025	0.221
		13	Contamination mixture	0.010	0.003	0.010	0.020	0.044	1.010	1.010	1.020	0.286
	Asthma	13	IVW	0.054	0.015	0.025	0.082	0.000	1.055	1.025	1.086	0.007
		13	Weighted median	0.046	0.019	0.008	0.084	0.019	1.047	1.008	1.087	0.171
		13	MR-Egger	0.033	0.028	-0.021	0.088	0.232	1.034	0.979	1.092	0.645
		13	MR-PRESSO	0.054	0.014	0.026	0.081	0.002	1.055	1.027	1.085	0.040
		13	Contamination mixture	0.060	0.026	0.030	0.130	0.010	1.062	1.030	1.139	0.112
	CAD	13	IVW	0.023	0.017	-0.010	0.056	0.165	1.023	0.990	1.057	0.576

Exposure	Outcome	n	Method	Estimate	StdError	CI95Lower	CI95Upper	p-value	OR	ORlow	ORhi	q-value
		13	Weighted median	-0.008	0.017	-0.041	0.025	0.652	0.992	0.960	1.026	0.926
		13	MR-Egger	-0.027	0.027	-0.081	0.026	0.316	0.973	0.923	1.026	0.731
		13	MR-PRESSO	0.023	0.017	-0.010	0.056	0.191	1.023	0.990	1.057	0.605
		13	Contamination mixture	0.078	0.043	-0.012	0.158	0.130	1.081	0.988	1.171	0.518
	MI	12	IVW	0.049	0.019	0.011	0.087	0.011	1.050	1.011	1.091	0.121
		12	Weighted median	0.019	0.025	-0.030	0.068	0.451	1.019	0.970	1.071	0.832
		12	MR-Egger	-0.018	0.036	-0.088	0.053	0.622	0.982	0.916	1.054	0.912
		12	MR-PRESSO	0.049	0.019	0.012	0.086	0.026	1.050	1.012	1.090	0.207
		12	Contamination mixture	0.104	0.048	0.014	0.204	0.031	1.110	1.014	1.227	0.231
	HF	12	IVW	0.015	0.020	-0.024	0.054	0.458	1.015	0.976	1.055	0.836
		12	Weighted median	0.011	0.025	-0.038	0.061	0.656	1.011	0.962	1.063	0.927
		12	MR-Egger	-0.006	0.037	-0.078	0.066	0.871	0.994	0.925	1.069	0.971
		12	MR-PRESSO	0.015	0.019	-0.022	0.051	0.448	1.015	0.978	1.053	0.828
		12	Contamination mixture	0.031	0.033	-0.029	0.101	0.301	1.031	0.971	1.106	0.724
	AF	13	IVW	0.052	0.017	0.019	0.085	0.002	1.053	1.019	1.089	0.035
		13	Weighted median	0.046	0.021	0.004	0.088	0.032	1.047	1.004	1.092	0.233
		13	MR-Egger	0.035	0.031	-0.026	0.096	0.265	1.035	0.974	1.100	0.685
		13	MR-PRESSO	0.052	0.013	0.027	0.077	0.001	1.053	1.028	1.080	0.026
		13	Contamination mixture	0.044	0.018	0.014	0.084	0.009	1.045	1.014	1.087	0.101
	Stroke	11	IVW	-0.010	0.018	-0.045	0.025	0.574	0.990	0.956	1.025	0.896
		11	Weighted median	-0.005	0.022	-0.047	0.038	0.835	0.996	0.954	1.039	0.971
		11	MR-Egger	-0.006	0.033	-0.070	0.059	0.858	0.994	0.932	1.060	0.971
		11	MR-PRESSO	-0.010	0.010	-0.030	0.010	0.343	0.990	0.971	1.010	0.752
		11	Contamination mixture	-0.003	0.020	-0.053	0.027	0.769	0.997	0.948	1.027	0.965
IL18	COPD	6	IVW	0.034	0.008	0.019	0.049	0.000	1.035	1.019	1.051	0.001
		6	Weighted median	0.031	0.009	0.013	0.049	0.001	1.031	1.013	1.050	0.019
		6	MR-Egger	0.018	0.021	-0.022	0.059	0.371	1.019	0.978	1.061	0.772
		6	MR-PRESSO	0.034	0.008	0.019	0.049	0.007	1.035	1.019	1.051	0.084
	Asthma	6	IVW	0.010	0.024	-0.036	0.057	0.660	1.010	0.965	1.058	0.928
		6	Weighted median	0.027	0.026	-0.025	0.078	0.306	1.027	0.976	1.081	0.727
		6	MR-Egger	0.078	0.057	-0.034	0.191	0.174	1.081	0.966	1.210	0.592
		6	MR-PRESSO	0.010	0.024	-0.036	0.057	0.678	1.010	0.965	1.058	0.932
	CAD	6	IVW	-0.004	0.019	-0.041	0.033	0.833	0.996	0.960	1.033	0.971
		6	Weighted median	0.004	0.022	-0.039	0.048	0.849	1.004	0.961	1.049	0.971
		6	MR-Egger	0.018	0.049	-0.079	0.115	0.720	1.018	0.924	1.121	0.945
		6	MR-PRESSO	-0.004	0.015	-0.032	0.025	0.797	0.996	0.968	1.025	0.971
	MI	6	IVW	0.011	0.029	-0.046	0.067	0.711	1.011	0.955	1.069	0.943
		6	Weighted median	0.015	0.034	-0.052	0.081	0.661	1.015	0.950	1.085	0.928

Exposure	Outcome	n	Method	Estimate	StdError	CI95Lower	CI95Upper	p-value	OR	ORlow	ORhi	q-value
		6	MR-Egger	0.000	0.075	-0.147	0.146	0.998	1.000	0.864	1.158	1.000
		6	MR-PRESSO	0.011	0.014	-0.017	0.038	0.477	1.011	0.984	1.039	0.847
	HF	6	IVW	0.039	0.029	-0.019	0.097	0.183	1.040	0.982	1.102	0.603
		6	Weighted median	0.047	0.034	-0.019	0.114	0.165	1.049	0.981	1.121	0.576
		6	MR-Egger	0.075	0.080	-0.081	0.231	0.344	1.078	0.923	1.260	0.752
		6	MR-PRESSO	0.039	0.018	0.003	0.075	0.084	1.040	1.003	1.078	0.409
	AF	6	IVW	0.075	0.028	0.021	0.129	0.007	1.078	1.021	1.137	0.083
		6	Weighted median	0.072	0.030	0.013	0.131	0.017	1.075	1.013	1.140	0.163
		6	MR-Egger	0.064	0.083	-0.099	0.227	0.442	1.066	0.906	1.255	0.824
		6	MR-PRESSO	0.075	0.028	0.021	0.129	0.043	1.078	1.021	1.137	0.284
	Stroke	5	IVW	-0.017	0.029	-0.074	0.039	0.552	0.983	0.929	1.040	0.893
		5	Weighted median	-0.017	0.033	-0.081	0.047	0.599	0.983	0.922	1.048	0.902
		5	MR-Egger	-0.059	0.091	-0.237	0.119	0.516	0.943	0.789	1.126	0.872
		5	MR-PRESSO	-0.017	0.029	-0.074	0.039	0.584	0.983	0.929	1.040	0.899
CX3CL1	COPD	8	IVW	-0.012	0.006	-0.025	0.000	0.058	0.988	0.976	1.000	0.333
		8	Weighted median	-0.008	0.008	-0.024	0.009	0.364	0.992	0.976	1.009	0.767
		8	MR-Egger	-0.005	0.022	-0.047	0.038	0.834	0.995	0.954	1.039	0.971
		8	MR-PRESSO	-0.012	0.006	-0.025	0.000	0.098	0.988	0.976	1.000	0.450
		8	Contamination mixture	-0.034	0.015	-0.054	0.006	0.199	0.966	0.947	1.006	0.614
	Asthma	8	IVW	-0.059	0.023	-0.104	-0.014	0.011	0.943	0.901	0.987	0.118
		8	Weighted median	-0.050	0.025	-0.098	-0.002	0.043	0.951	0.907	0.998	0.284
		8	MR-Egger	-0.001	0.075	-0.149	0.147	0.988	0.999	0.862	1.158	1.000
		8	MR-PRESSO	-0.059	0.023	-0.104	-0.014	0.038	0.943	0.901	0.987	0.264
		8	Contamination mixture	-0.120	0.054	-0.210	0.000	0.054	0.887	0.810	1.000	0.321
	CAD	8	IVW	0.078	0.015	0.048	0.107	0.000	1.081	1.049	1.113	0.000
		8	Weighted median	0.062	0.019	0.024	0.099	0.001	1.064	1.024	1.104	0.025
		8	MR-Egger	0.084	0.048	-0.011	0.179	0.084	1.087	0.989	1.195	0.409
		8	MR-PRESSO	0.078	0.012	0.055	0.100	0.000	1.081	1.057	1.105	0.008
		8	Contamination mixture	0.080	0.015	0.050	0.110	0.000	1.083	1.051	1.116	0.005
	MI	8	IVW	0.113	0.023	0.068	0.158	0.000	1.120	1.071	1.172	0.000
		8	Weighted median	0.100	0.029	0.043	0.157	0.001	1.105	1.044	1.170	0.014
		8	MR-Egger	0.086	0.074	-0.059	0.230	0.244	1.090	0.943	1.259	0.658
		8	MR-PRESSO	0.113	0.014	0.086	0.141	0.000	1.120	1.090	1.151	0.003
		8	Contamination mixture	0.119	0.026	0.069	0.169	0.000	1.127	1.072	1.184	0.004
	HF	8	IVW	0.032	0.027	-0.021	0.085	0.242	1.032	0.979	1.088	0.657
		8	Weighted median	0.015	0.033	-0.049	0.080	0.646	1.015	0.952	1.083	0.924
		8	MR-Egger	0.112	0.087	-0.057	0.282	0.195	1.119	0.944	1.326	0.611
		8	MR-PRESSO	0.032	0.027	-0.021	0.085	0.280	1.032	0.979	1.088	0.704

Exposure	Outcome	n	Method	Estimate	StdError	CI95Lower	CI95Upper	p-value	OR	ORlow	ORhi	q-value
		8	Contamination mixture	0.001	0.071	-0.079	0.201	0.931	1.001	0.924	1.223	0.993
	AF	8	IVW	0.020	0.023	-0.025	0.065	0.386	1.020	0.975	1.067	0.786
		8	Weighted median	0.021	0.027	-0.033	0.075	0.442	1.021	0.968	1.078	0.824
		8	MR-Egger	0.105	0.071	-0.033	0.243	0.137	1.111	0.967	1.276	0.528
		8	MR-PRESSO	0.020	0.023	-0.025	0.065	0.415	1.020	0.975	1.067	0.808
		8	Contamination mixture	0.010	0.069	-0.130	0.140	0.767	1.010	0.878	1.150	0.965
	Stroke	8	IVW	0.054	0.022	0.010	0.097	0.015	1.055	1.010	1.102	0.152
		8	Weighted median	0.043	0.028	-0.012	0.098	0.125	1.044	0.988	1.103	0.512
		8	MR-Egger	-0.001	0.071	-0.140	0.138	0.989	0.999	0.870	1.148	1.000
		8	MR-PRESSO	0.054	0.014	0.027	0.081	0.006	1.055	1.027	1.084	0.077
		8	Contamination mixture	0.057	0.038	0.017	0.167	0.029	1.058	1.017	1.181	0.221
VEGFA	COPD	18	IVW	0.011	0.005	0.001	0.021	0.024	1.011	1.001	1.021	0.198
		18	Weighted median	0.008	0.005	-0.002	0.018	0.115	1.008	0.998	1.019	0.485
		18	MR-Egger	0.005	0.009	-0.011	0.022	0.527	1.005	0.989	1.022	0.880
		18	MR-PRESSO	0.011	0.005	0.001	0.021	0.038	1.011	1.001	1.021	0.261
		18	Contamination mixture	0.014	0.003	0.004	0.014	0.043	1.014	1.004	1.014	0.284
	Asthma	18	IVW	-0.009	0.013	-0.034	0.017	0.504	0.991	0.967	1.017	0.869
		18	Weighted median	0.004	0.015	-0.026	0.033	0.812	1.004	0.974	1.034	0.971
		18	MR-Egger	0.008	0.023	-0.036	0.052	0.719	1.008	0.964	1.054	0.945
		18	MR-PRESSO	-0.009	0.013	-0.034	0.017	0.513	0.991	0.967	1.017	0.871
		18	Contamination mixture	-0.005	0.023	-0.075	0.015	1.000	0.995	0.928	1.015	1.000
	CAD	18	IVW	-0.003	0.011	-0.025	0.020	0.821	0.997	0.975	1.020	0.971
		18	Weighted median	-0.007	0.012	-0.031	0.018	0.594	0.993	0.969	1.018	0.900
		18	MR-Egger	0.004	0.020	-0.035	0.044	0.827	1.004	0.966	1.045	0.971
		18	MR-PRESSO	-0.003	0.011	-0.025	0.020	0.824	0.997	0.975	1.020	0.971
		18	Contamination mixture	-0.012	0.008	-0.022	0.008	0.495	0.988	0.978	1.008	0.860
	MI	17	IVW	-0.011	0.015	-0.041	0.018	0.444	0.989	0.960	1.018	0.824
		17	Weighted median	-0.022	0.019	-0.059	0.015	0.236	0.978	0.943	1.015	0.650
		17	MR-Egger	-0.031	0.025	-0.080	0.019	0.227	0.970	0.923	1.019	0.638
		17	MR-PRESSO	-0.011	0.013	-0.037	0.014	0.394	0.989	0.964	1.014	0.796
		17	Contamination mixture	-0.030	0.015	-0.060	0.000	0.152	0.971	0.942	1.000	0.550
	HF	18	IVW	0.029	0.017	-0.004	0.063	0.087	1.030	0.996	1.065	0.418
		18	Weighted median	0.009	0.020	-0.030	0.048	0.652	1.009	0.970	1.050	0.926
		18	MR-Egger	-0.029	0.026	-0.080	0.023	0.275	0.972	0.923	1.023	0.697
		18	MR-PRESSO	0.029	0.017	-0.004	0.063	0.105	1.030	0.996	1.065	0.467
		18	Contamination mixture	0.138	0.056	-0.012	0.208	0.108	1.148	0.988	1.231	0.475
	AF	18	IVW	0.009	0.015	-0.021	0.039	0.556	1.009	0.979	1.040	0.896
		18	Weighted median	0.004	0.017	-0.029	0.038	0.794	1.004	0.971	1.039	0.971

Exposure	Outcome	n	Method	Estimate	StdError	CI95Lower	CI95Upper	p-value	OR	ORlow	ORhi	q-value
		18	MR-Egger	0.021	0.027	-0.033	0.074	0.451	1.021	0.968	1.077	0.832
		18	MR-PRESSO	0.009	0.015	-0.021	0.039	0.564	1.009	0.979	1.040	0.896
		18	Contamination mixture	0.010	0.023	-0.020	0.070	0.584	1.010	0.980	1.072	0.899
	Stroke	18	IVW	0.016	0.016	-0.015	0.048	0.315	1.016	0.985	1.049	0.731
		18	Weighted median	-0.014	0.022	-0.058	0.030	0.544	0.986	0.944	1.031	0.891
		18	MR-Egger	0.002	0.029	-0.055	0.060	0.937	1.002	0.946	1.061	0.995
		18	MR-PRESSO	0.016	0.016	-0.015	0.048	0.329	1.016	0.985	1.049	0.737
		18	Contamination mixture	0.001	0.046	-0.049	0.131	0.922	1.001	0.952	1.140	0.988
CRP	COPD	5	IVW	0.024	0.008	0.008	0.041	0.004	1.025	1.008	1.042	0.055
		5	Weighted median	0.022	0.010	0.003	0.042	0.026	1.022	1.003	1.043	0.206
		5	MR-Egger	-0.007	0.026	-0.058	0.044	0.782	0.993	0.944	1.044	0.971
		5	MR-PRESSO	0.024	0.007	0.011	0.037	0.021	1.025	1.011	1.038	0.179
	Asthma	5	IVW	0.001	0.024	-0.046	0.048	0.974	1.001	0.955	1.049	1.000
		5	Weighted median	-0.011	0.028	-0.066	0.044	0.690	0.989	0.936	1.045	0.935
		5	MR-Egger	-0.118	0.074	-0.263	0.026	0.109	0.888	0.769	1.027	0.475
		5	MR-PRESSO	0.001	0.022	-0.043	0.044	0.973	1.001	0.958	1.045	1.000
	CAD	5	IVW	-0.012	0.023	-0.058	0.033	0.597	0.988	0.944	1.034	0.901
		5	Weighted median	-0.019	0.024	-0.066	0.029	0.440	0.981	0.936	1.029	0.824
		5	MR-Egger	-0.148	0.064	-0.274	-0.023	0.020	0.862	0.760	0.977	0.175
		5	MR-PRESSO	-0.012	0.023	-0.058	0.033	0.625	0.988	0.944	1.034	0.912
	MI	5	IVW	0.009	0.031	-0.051	0.069	0.769	1.009	0.950	1.071	0.965
		5	Weighted median	-0.005	0.034	-0.072	0.062	0.883	0.995	0.930	1.064	0.974
		5	MR-Egger	-0.129	0.095	-0.315	0.058	0.177	0.879	0.730	1.060	0.594
		5	MR-PRESSO	0.009	0.031	-0.051	0.069	0.783	1.009	0.950	1.071	0.971
	HF	5	IVW	0.005	0.032	-0.057	0.068	0.868	1.005	0.944	1.070	0.971
		5	Weighted median	0.004	0.037	-0.069	0.078	0.906	1.004	0.933	1.081	0.981
		5	MR-Egger	0.125	0.111	-0.092	0.343	0.258	1.134	0.912	1.409	0.675
		5	MR-PRESSO	0.005	0.022	-0.037	0.048	0.818	1.005	0.964	1.049	0.971
	AF	5	IVW	0.027	0.035	-0.042	0.095	0.445	1.027	0.959	1.100	0.824
		5	Weighted median	0.049	0.031	-0.012	0.111	0.114	1.051	0.988	1.117	0.485
		5	MR-Egger	0.125	0.124	-0.118	0.369	0.314	1.133	0.888	1.446	0.731
		5	MR-PRESSO	0.027	0.035	-0.042	0.095	0.487	1.027	0.959	1.100	0.855
	Stroke	4	IVW	-0.013	0.037	-0.086	0.060	0.727	0.987	0.918	1.062	0.948
		4	Weighted median	0.012	0.036	-0.058	0.082	0.738	1.012	0.943	1.086	0.953
		4	MR-Egger	-0.127	0.148	-0.417	0.164	0.393	0.881	0.659	1.178	0.794
CCL17	COPD	3	IVW	-0.008	0.007	-0.021	0.005	0.217	0.992	0.979	1.005	0.627
		3	Weighted median	-0.008	0.007	-0.022	0.006	0.268	0.992	0.978	1.006	0.689
		3	MR-Egger	-0.017	0.023	-0.063	0.028	0.455	0.983	0.939	1.029	0.835

Exposure	Outcome	n	Method	Estimate	StdError	CI95Lower	CI95Upper	p-value	OR	ORlow	ORhi	q-value
	Asthma	3	IVW	-0.054	0.019	-0.091	-0.017	0.004	0.947	0.913	0.983	0.062
		3	Weighted median	-0.050	0.021	-0.091	-0.008	0.020	0.951	0.913	0.992	0.173
		3	MR-Egger	-0.003	0.065	-0.131	0.125	0.958	0.997	0.877	1.133	0.999
	CAD	3	IVW	0.042	0.015	0.012	0.072	0.007	1.043	1.012	1.075	0.081
		3	Weighted median	0.043	0.018	0.008	0.078	0.016	1.044	1.008	1.081	0.155
		3	MR-Egger	0.034	0.064	-0.091	0.159	0.598	1.034	0.913	1.172	0.902
	MI	3	IVW	0.056	0.023	0.010	0.102	0.017	1.057	1.010	1.107	0.163
		3	Weighted median	0.057	0.028	0.002	0.112	0.042	1.059	1.002	1.119	0.279
		3	MR-Egger	0.028	0.141	-0.248	0.304	0.843	1.028	0.781	1.355	0.971
	HF	3	IVW	0.016	0.025	-0.033	0.065	0.524	1.016	0.967	1.068	0.879
		3	Weighted median	0.012	0.028	-0.042	0.066	0.661	1.012	0.959	1.068	0.928
		3	MR-Egger	-0.022	0.087	-0.193	0.150	0.805	0.979	0.825	1.162	0.971
	AF	3	IVW	0.023	0.021	-0.018	0.064	0.281	1.023	0.982	1.066	0.704
		3	Weighted median	0.022	0.023	-0.024	0.068	0.344	1.022	0.977	1.070	0.753
		3	MR-Egger	-0.036	0.072	-0.178	0.106	0.623	0.965	0.837	1.112	0.912
	Stroke	3	IVW	0.030	0.023	-0.015	0.076	0.191	1.031	0.985	1.079	0.605
		3	Weighted median	0.045	0.026	-0.006	0.097	0.083	1.047	0.994	1.102	0.409
		3	MR-Egger	0.096	0.095	-0.090	0.282	0.310	1.101	0.914	1.326	0.727
CCL8	COPD	5	IVW	-0.005	0.002	-0.009	-0.001	0.016	0.995	0.991	0.999	0.157
		5	Weighted median	-0.003	0.002	-0.007	0.001	0.140	0.997	0.993	1.001	0.531
		5	MR-Egger	-0.003	0.004	-0.010	0.005	0.536	0.997	0.990	1.005	0.888
		5	MR-PRESSO	-0.005	0.002	-0.009	-0.001	0.074	0.995	0.991	0.999	0.380
	Asthma	5	IVW	0.001	0.005	-0.009	0.012	0.826	1.001	0.991	1.012	0.971
		5	Weighted median	0.002	0.006	-0.010	0.014	0.723	1.002	0.990	1.014	0.946
		5	MR-Egger	-0.001	0.010	-0.021	0.019	0.921	0.999	0.979	1.019	0.988
		5	MR-PRESSO	0.001	0.002	-0.004	0.006	0.649	1.001	0.996	1.006	0.924
	CAD	5	IVW	0.002	0.008	-0.014	0.019	0.782	1.002	0.986	1.019	0.971
		5	Weighted median	0.003	0.005	-0.008	0.013	0.619	1.003	0.992	1.013	0.912
		5	MR-Egger	0.016	0.016	-0.015	0.048	0.309	1.016	0.985	1.049	0.727
		5	MR-PRESSO	0.002	0.008	-0.014	0.019	0.796	1.002	0.986	1.019	0.971
	MI	5	IVW	-0.002	0.006	-0.014	0.011	0.802	0.998	0.986	1.011	0.971
	MI	5	Weighted median	-0.001	0.007	-0.015	0.013	0.905	0.999	0.985	1.013	0.981
	MI	5	MR-Egger	0.012	0.013	-0.012	0.037	0.327	1.012	0.988	1.037	0.734
	MI	5	MR-PRESSO	-0.002	0.006	-0.013	0.009	0.787	0.998	0.988	1.009	0.971
	HF	5	IVW	0.011	0.007	-0.003	0.025	0.122	1.011	0.997	1.025	0.504
	HF	5	Weighted median	0.013	0.008	-0.003	0.028	0.108	1.013	0.997	1.029	0.475
	HF	5	MR-Egger	0.026	0.014	0.000	0.053	0.054	1.026	1.000	1.054	0.321

Exposure	Outcome	n	Method	Estimate	StdError	CI95Lower	CI95Upper	p-value	OR	ORlow	ORhi	q-value
	HF	5	MR-PRESSO	0.011	0.007	-0.003	0.024	0.188	1.011	0.997	1.025	0.605
	AF	5	IVW	0.009	0.006	-0.003	0.021	0.139	1.009	0.997	1.021	0.529
	AF	5	Weighted median	0.010	0.007	-0.003	0.023	0.138	1.010	0.997	1.023	0.528
	AF	5	MR-Egger	0.013	0.011	-0.009	0.036	0.249	1.013	0.991	1.036	0.664
	AF	5	MR-PRESSO	0.009	0.002	0.004	0.013	0.020	1.009	1.004	1.014	0.174
		5	IVW	0.009	0.007	-0.004	0.022	0.190	1.009	0.996	1.022	0.605
		5	Weighted median	0.005	0.007	-0.009	0.019	0.464	1.005	0.991	1.019	0.838
		5	MR-Egger	0.004	0.015	-0.024	0.033	0.776	1.004	0.976	1.033	0.965
		5	MR-PRESSO	0.009	0.007	-0.004	0.022	0.260	1.009	0.996	1.022	0.677
CCL22	COPD	3	IVW	0.017	0.008	0.001	0.032	0.032	1.017	1.001	1.033	0.233
		3	Weighted median	0.004	0.011	-0.017	0.025	0.683	1.004	0.984	1.026	0.932
		3	MR-Egger	0.040	0.076	-0.110	0.190	0.598	1.041	0.896	1.210	0.902
	Asthma	3	IVW	0.052	0.022	0.009	0.095	0.018	1.053	1.009	1.100	0.167
		3	Weighted median	0.043	0.034	-0.023	0.109	0.198	1.044	0.978	1.116	0.614
		3	MR-Egger	0.036	0.247	-0.448	0.519	0.885	1.036	0.639	1.681	0.974
	CAD	3	IVW	-0.031	0.018	-0.067	0.004	0.080	0.969	0.936	1.004	0.400
		3	Weighted median	-0.062	0.025	-0.111	-0.013	0.012	0.940	0.895	0.987	0.133
		3	MR-Egger	0.072	0.158	-0.237	0.381	0.647	1.075	0.789	1.464	0.924
	MI	3	IVW	-0.040	0.026	-0.092	0.012	0.128	0.961	0.912	1.012	0.516
		3	Weighted median	-0.093	0.037	-0.167	-0.020	0.012	0.911	0.847	0.980	0.132
		3	MR-Egger	0.154	0.277	-0.389	0.696	0.579	1.166	0.678	2.005	0.896
	HF	3	IVW	-0.012	0.029	-0.069	0.046	0.689	0.988	0.933	1.047	0.935
		3	Weighted median	-0.002	0.041	-0.082	0.079	0.969	0.998	0.922	1.082	1.000
		3	MR-Egger	-0.001	0.244	-0.480	0.478	0.997	0.999	0.619	1.613	1.000
	AF	3	IVW	-0.057	0.025	-0.106	-0.009	0.019	0.944	0.900	0.991	0.171
		3	Weighted median	-0.046	0.031	-0.106	0.014	0.130	0.955	0.899	1.014	0.518
		3	MR-Egger	-0.077	0.083	-0.239	0.085	0.351	0.926	0.787	1.089	0.757
	Stroke	3	IVW	-0.005	0.027	-0.059	0.048	0.851	0.995	0.943	1.050	0.971
		3	Weighted median	-0.005	0.032	-0.068	0.057	0.866	0.995	0.935	1.058	0.971
		3	MR-Egger	0.004	0.092	-0.176	0.185	0.965	1.004	0.838	1.203	1.000
IL1R1	COPD	5	IVW	0.006	0.024	-0.040	0.053	0.786	1.006	0.961	1.054	0.971
		5	Weighted median	0.000	0.015	-0.029	0.030	0.982	1.000	0.971	1.030	1.000
		5	MR-Egger	-0.028	0.131	-0.283	0.228	0.833	0.973	0.753	1.256	0.971
		3	MR-PRESSO	-0.008	0.012	-0.032	0.016	0.580	0.992	0.968	1.016	0.896
	Asthma	5	IVW	0.186	0.158	-0.123	0.496	0.238	1.205	0.884	1.642	0.653
		5	Weighted median	0.367	0.050	0.268	0.465	0.000	1.443	1.308	1.592	0.000
		5	MR-Egger	0.867	0.779	-0.660	2.393	0.266	2.379	0.517	10.948	0.686

Exposure	Outcome	n	Method	Estimate	StdError	CI95Lower	CI95Upper	p-value	OR	ORlow	ORhi	q-value
		1	MR-PRESSO	0.281	NA	NA	NA	NA	1.324	NA	NA	NA
	CAD	5	IVW	0.084	0.033	0.020	0.148	0.010	1.088	1.020	1.160	0.110
		5	Weighted median	0.058	0.034	-0.008	0.124	0.084	1.060	0.992	1.132	0.409
		5	MR-Egger	-0.127	0.133	-0.387	0.133	0.339	0.881	0.679	1.142	0.750
		5	MR-PRESSO	0.084	0.033	0.020	0.148	0.061	1.088	1.020	1.160	0.342
	MI	5	IVW	0.123	0.057	0.012	0.234	0.029	1.131	1.012	1.264	0.224
		5	Weighted median	0.110	0.051	0.010	0.210	0.031	1.117	1.010	1.234	0.230
		5	MR-Egger	0.015	0.310	-0.592	0.622	0.962	1.015	0.553	1.862	0.999
		5	MR-PRESSO	0.123	0.057	0.012	0.234	0.095	1.131	1.012	1.264	0.445
	HF	5	IVW	-0.058	0.041	-0.137	0.022	0.155	0.944	0.872	1.022	0.557
		5	Weighted median	-0.069	0.048	-0.162	0.025	0.149	0.933	0.850	1.025	0.548
		5	MR-Egger	0.069	0.197	-0.317	0.455	0.726	1.071	0.728	1.576	0.948
		5	MR-PRESSO	-0.058	0.025	-0.106	-0.010	0.078	0.944	0.900	0.990	0.395
	AF	5	IVW	-0.073	0.041	-0.153	0.007	0.074	0.930	0.858	1.007	0.382
		5	Weighted median	-0.068	0.045	-0.156	0.020	0.127	0.934	0.855	1.020	0.516
		5	MR-Egger	-0.056	0.228	-0.503	0.390	0.805	0.945	0.605	1.477	0.971
		5	MR-PRESSO	-0.073	0.041	-0.153	0.007	0.149	0.930	0.858	1.007	0.548
	Stroke	5	IVW	0.072	0.036	0.001	0.142	0.048	1.074	1.001	1.153	0.300
		5	Weighted median	0.079	0.045	-0.009	0.168	0.080	1.082	0.991	1.183	0.399
		5	MR-Egger	0.248	0.179	-0.103	0.599	0.166	1.282	0.902	1.821	0.576
		5	MR-PRESSO	0.072	0.031	0.010	0.133	0.086	1.074	1.010	1.143	0.413

Blue highlight denotes suggestive evidence based on FDR-corrected p-value (q-value). Orange highlight denotes strong evidence based on FDR-corrected p-value (q-value).

CI95Lower: 95% lower CI for the estimate; Ci95Upper: 95% upper CI for the estimate. ORlow: 95% lower CI for the OR; ORhi: 95% upper CI for the OR. AF: atrial fibrillation, CAD: coronary artery disease, COPD: chronic obstructive pulmonary diseases, HF: heart failure, IS: ischemia stroke, MI: myocardial infarction, n: number of SNPs used as instrument variables in each method, SNPs single nucleotide polymorphism, CI confidence intervals, OR odds ratio.
