# Supplementary material for: Understanding the bidirectional relationship between chronic respiratory disease and cardiovascular disease using genetic evidence
Source: Thorax. 2025 Nov 15;81(5):e222908. doi: 10.1136/thorax-2024-222908 (PMC13151528; doi:10.1136/thorax-2024-222908)
Supplement: online supplemental file 2 [file thorax-81-5-s002.pptx]

## Slide 1
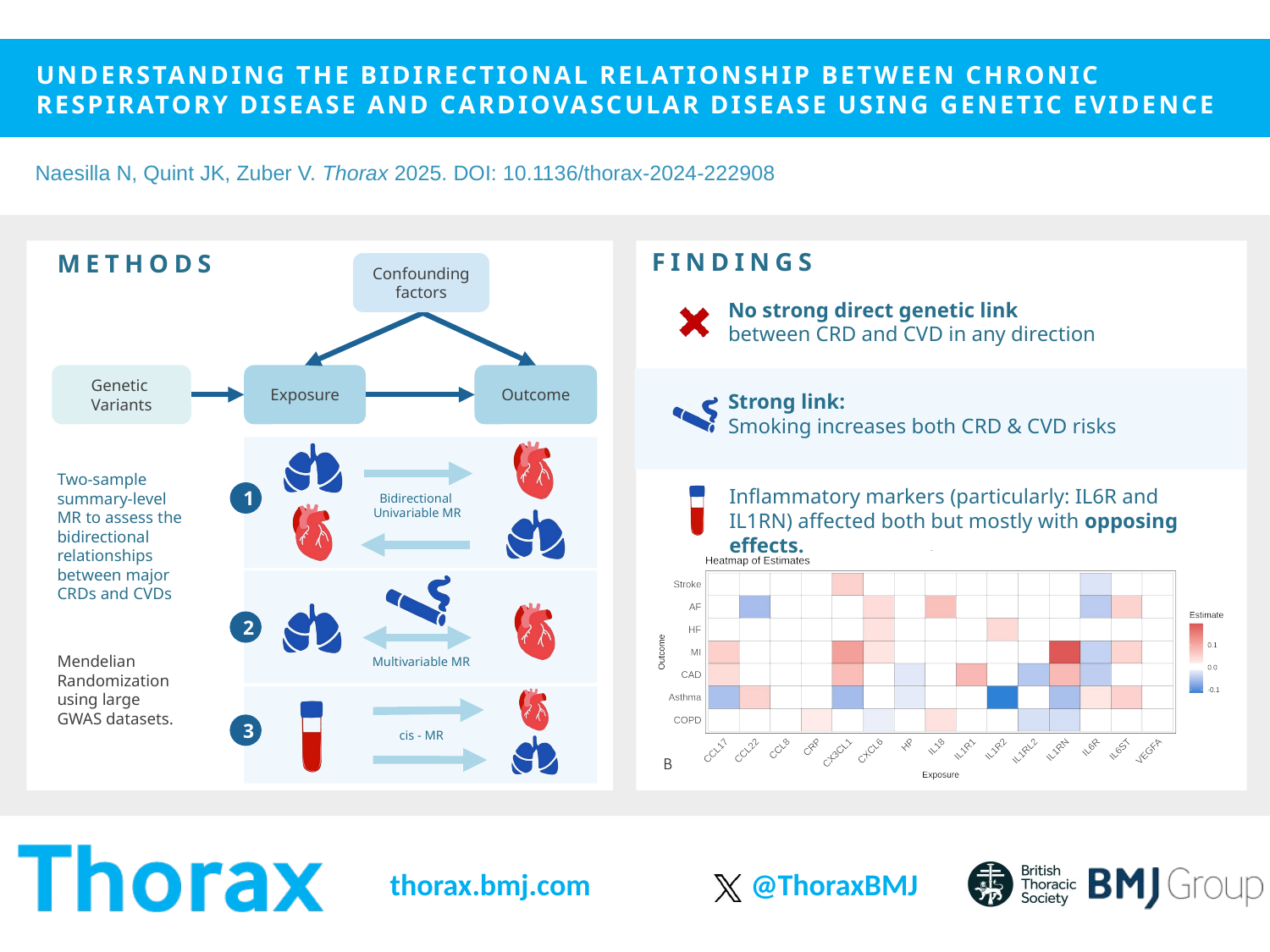

UNDERSTANDING THE BIDIRECTIONAL RELATIONSHIP BETWEEN CHRONIC RESPIRATORY DISEASE AND CARDIOVASCULAR DISEASE USING GENETIC EVIDENCE
Naesilla N, Quint JK, Zuber V. Thorax 2025. DOI: 10.1136/thorax-2024-222908
FINDINGS
METHODS
Confounding factors
Outcome
Exposure
Genetic
Variants
Manuscript Title
No strong direct genetic link
between CRD and CVD in any direction
Strong link:
Smoking increases both CRD & CVD risks
Two-sample summary-level MR to assess the bidirectional relationships between major CRDs and CVDs
Inflammatory markers (particularly: IL6R and IL1RN) affected both but mostly with opposing effects.
1
Bidirectional
Univariable MR
2
Mendelian Randomization using large GWAS datasets.
Multivariable MR
© Author(s) (or their employer(s) 2019. Re-use permitted under CC BY. Published by BMJ.
3
cis - MR
thorax.bmj.com @ThoraxBMJ
